# Global patterns and health impact of unintentional injuries among children and adolescents, 1990–2021

**DOI:** 10.3389/fpubh.2025.1626739

**Published:** 2025-09-24

**Authors:** Wei Wei, Wei Lyu, Yuchen Zhang

**Affiliations:** ^1^Zhengzhou College of Finance and Economics, Zhengzhou, China; ^2^Faculty of Medicine and Health, Al-Farabi Kazakh National University, Almaty, Kazakhstan; ^3^Henan University of Technology, Zhengzhou, China

**Keywords:** unintentional injuries, children and adolescents, incidence, mortality, DALYs - disability-adjusted life years, BAPC prediction model

## Abstract

**Summary:**

Unintentional injuries significantly threaten the health of children and adolescents globally. Annually, injuries result in approximately five million deaths worldwide, with around 12% occurring in children. This study investigates global trends in incidence, mortality, and disability-adjusted life years (DALYs) associated with unintentional injuries among children and adolescents from 1990 to 2021.

**Methods:**

We conducted an analysis of global unintentional injury incidence, mortality, and DALYs among individuals aged 0–19 years using data from the Global Burden of Disease (GBD) database. Incidence, mortality, and DALY rates per 100,000 population were calculated, accompanied by 95% uncertainty intervals (UI). Data from 204 countries and territories were stratified by age, sex, and geographic region. Trend analyses utilized Joinpoint regression modeling to calculate annual percentage changes (APC) and log-transformed linear regression to estimate the estimated annual percentage change (EAPC). Projections of injury burden through 2035 were generated using Bayesian age-period-cohort (BAPC) modeling.

**Findings:**

Globally, in 2021, unintentional injuries in children and adolescents resulted in 157,808,979 cases (95% UI: 138,280,367–179,472,942), 305,596 deaths (95% UI: 240,004–365,146), and 28,410,167 DALYs (95% UI: 23,100,433–33,513,238). Among the five socio-demographic index (SDI) regions, the low-middle SDI region exhibited the most significant decrease in injury incidence. Regionally, from 1990 to 2021, the Caribbean experienced the largest relative increase in injury incidence (EAPC 0.25, 95% CI: −0.60 to 1.11). At the country level, India reported the highest absolute number of cases (24,115,574; 95% UI: 21,137,414–27,296,091). BAPC modeling predicts a continued reduction in the global burden of unintentional injuries up to 2035.

**Interpretation:**

Global trends demonstrate declining incidence, mortality, and DALYs associated with unintentional injuries among children and adolescents. Comprehensive epidemiological insights are critical for enhancing targeted prevention strategies and control measures to mitigate injury burden in this vulnerable population.

## Introduction

Unintentional injuries are a major threat to child and adolescent health worldwide (1). Each year, injuries claim about 5 million lives of all ages, and roughly 12% of these deaths occur in children (2). Unintentional causes – such as road traffic crashes, drowning, burns, falls, and poisonings – account for the vast majority of injury deaths in those under 20 years old (3). In total, over 900,000 children and adolescents under the age of 18 die annually from unintentional injuries making injuries one of the leading causes of death and disability in this young population (4). As childhood infectious disease mortality has declined in recent decades, injuries now represent an increasingly prominent cause of preventable loss of life and health. Thousands of children who survive early childhood illnesses are later killed or disabled by injuries and injuries (both unintentional and intentional) constitute about one-third of all deaths among adolescents globally (5, 6). These figures underscore the urgent public health significance of child and adolescent injuries. The burden of unintentional injuries is unevenly distributed, with low- and middle-income countries (LMICs) bearing a disproportionate share. The majority of child injury deaths occur in LMIC settings, where safety regulations are limited and access to prompt trauma care is often inadequate (7). For example, an estimated 1,900 children and adolescents die every day from injuries, and most of these fatalities are in low-SDI (Socio-demographic Index) regions (8). By contrast, high-income countries have achieved substantial reductions in child injury mortality through improved safety legislation (e.g., road safety laws, pool fencing, smoke alarms) and strengthened health systems. Nevertheless, even in high-SDI settings, injuries remain a leading cause of pediatric mortality (9), indicating that no country is immune to this challenge. Major causes of unintentional injury deaths vary by context – road traffic injuries and drowning are among the top killers globally, while falls contribute heavily to non-fatal injury burden – but in all regions, these causes are largely preventable.

In light of this significant yet preventable burden, there is a strong rationale for a comprehensive global trend analysis of child and adolescent injuries. Understanding long-term trends and disparities in injury incidence and outcomes is crucial for guiding prevention efforts and policy. Previous analyses have highlighted the heavy toll of transport and other unintentional injuries among youth (10), but an updated global assessment is needed to evaluate progress over the past three decades and to inform future strategies. Using the latest Global Burden of Disease (GBD) 2021 data, which includes comprehensive estimates for 204 countries and territories up to the year 2021, we can quantify trends in injury incidence, mortality, and disability-adjusted life years (DALYs) with consistent methods. Such an approach allows us to monitor whether interventions and socioeconomic changes since 1990 have reduced the burden, and to identify persistent gaps.

## Methods

### Overview and methodological details

The GBD Study 2021 provides a comprehensive assessment of health loss associated with 369 diseases, injuries, and disabilities, alongside 88 risk factors, across 204 countries and territories, employing the most recent epidemiological data available. The GBD database utilizes advanced statistical methodologies to address missing data and adjust for potential confounding factors, as extensively detailed in previous GBD literature (11, 12). The GBD framework facilitates comparative evaluations of incidence, mortality, and DALYs at national, regional, and global levels. In the GBD analysis, disease burden is quantified using three key metrics: mortality, incidence, and DALYs. DALYs represent the sum of years of life lost (YLL) due to premature death and years lived with disability (YLD), calculated as defined in Equations 1 and 2:


(1) $$\begin{array}{l} \mathrm{YLL}=\text{number of deaths}\times \text{standard life}\;\\ \text{expectancy}\;\mathrm{at}\;\mathrm{age}\;\text{of death} \end{array}$$

(2) YLD=prevalence×disability weight

Disability weights are determined through expert consensus and range from 0 (perfect health) to 1 (death). This comprehensive approach ensures a scientifically robust understanding of the global burden of diseases and injuries (13).

This study extracted and analyzed data from the GBD 2021 database^1^ on unintentional injury cases, incidence, mortality, and DALYs among children and adolescents aged 0–19 years, covering the period from 1990 to 2021 for 204 countries and territories. Participants were categorized into five age groups: <1 year, 1–4 years, 5–9 years, 10–14 years, and 15–19 years. Data on case counts, incidence rates, mortality rates, and DALYs were summarized globally, regionally, and nationally. GBD data do not include racial or ethnic classifications. The study adhered to the Guidelines for Accurate and Transparent Health Estimates Reporting (GATHER) (14).

### SDI

SDI is a composite indicator derived from fertility rates, education levels, and per-capita income, reflecting the developmental status of countries or regions on a scale from 0 to 1, where higher values indicate greater socio-economic development (15). SDI has been previously associated with variations in disease incidence and mortality (16). Countries and geographic regions were grouped into five SDI categories (low, low-middle, middle, high-middle, and high) to examine the relationship between unintentional injuries in children and adolescents and socioeconomic development.

### Data quality, uncertainty, and potential biases in GBD estimates

The GBD 2021 estimates integrate heterogeneous sources (vital registration, verbal autopsy, hospital/ED records, surveys, and police/transport registries) and standardized modeling to address missingness and misclassification. However, data availability and quality vary substantially by country and over time, particularly in low- and middle-income settings where injury surveillance and civil registration systems are incomplete. Under-registration of deaths, cause-of-death misclassification (e.g., undetermined intent, transport-related coding inconsistencies), and sparse non-fatal data may introduce bias. GBD applies redistribution algorithms, covariate-informed ensemble models, and uncertainty propagation to mitigate these issues, but residual error remains. Therefore, all point estimates should be interpreted with their 95% uncertainty intervals (UIs), and cross-country comparisons, especially in data-poor contexts, warrant caution. We followed GATHER reporting standards.

### Projections

The Bayesian age–period–cohort (BAPC) framework assumes smooth age, period, and cohort effects with random-walk priors, effectively extrapolating recent log-linear period trends while borrowing strength across age and cohort. Projections implicitly maintain recent structural relationships unless new information enters the system; they do not endogenously incorporate step-changes from disruptive policies, technologies, or shocks.

### Statistical analysis

Incidence, mortality, and DALY rates per 100,000 population were calculated with corresponding 95% UIs, as provided by the GBD database (17). Annual percentage changes (APCs) and their 95% confidence intervals (CIs) were computed using Joinpoint regression models to evaluate internal trends within distinct time intervals (18). This method offers detailed insights into annual fluctuations, providing a granular view of year-to-year variations. Average annual percentage changes (EAPCs) and their CIs were calculated using log-transformed linear regression models to assess temporal trends in incidence, mortality, and DALYs from 1990 to 2021 (19, 20). The EAPC metric is particularly valuable for examining long-term trends independent of short-term variations, with values and their 95% CI lower bounds greater than zero indicating upward trends, and values with upper bounds less than zero indicating downward trends. Curve fitting was performed to explore associations between disease burden indicators and SDI. All analyses were conducted using R software version 4.4.2, with statistical significance set at *p* < 0.05.

## Result

### Global burden trends

#### Incidence

**Figure 1:**
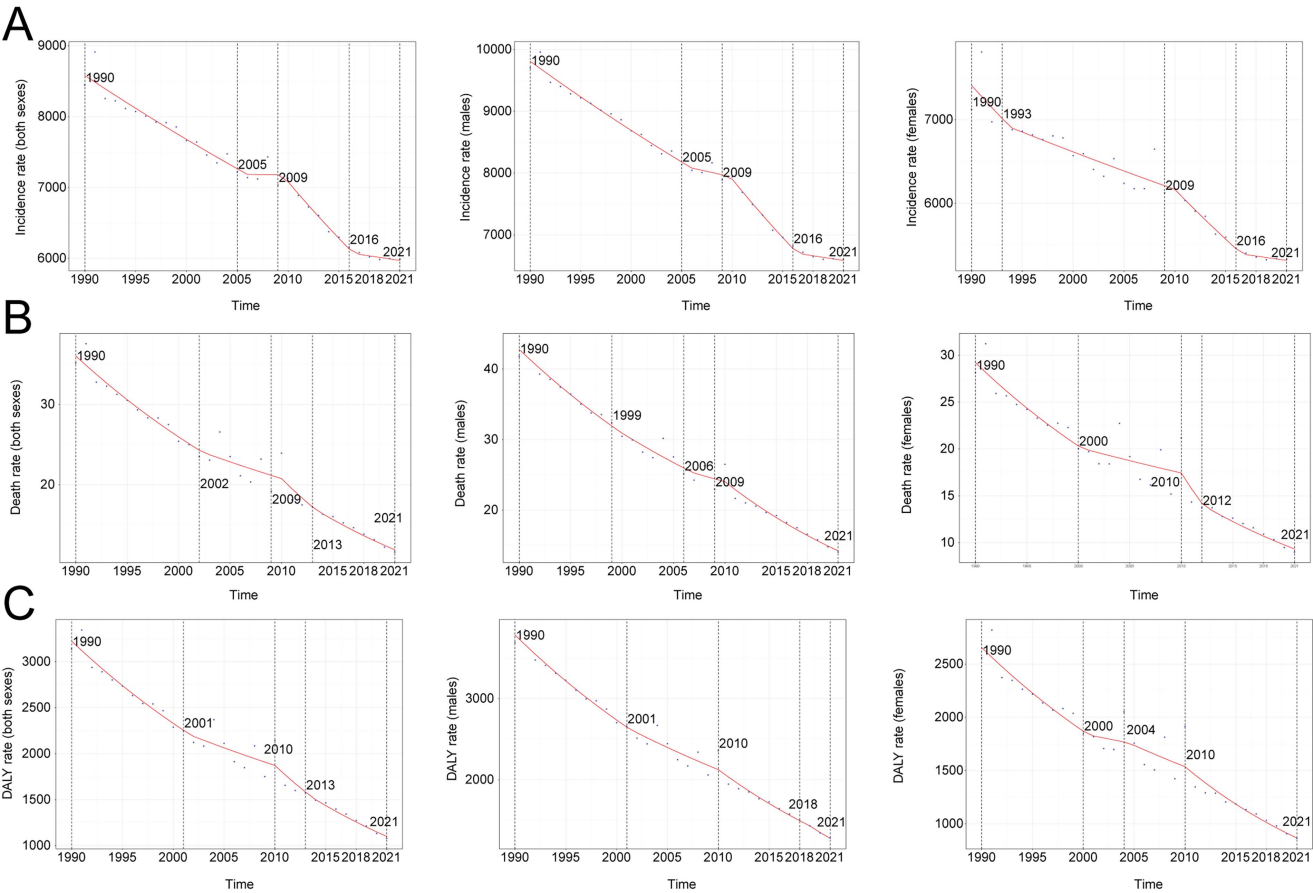
Annual percent change (APC) and trends in global unintentional injuries incidence, mortality, and disability-adjusted life years (DALYs) from 1990 to 2021. **(A)** Incidence rate. **(B)** Mortality rate. **(C)** DALYs rate.

**Figure 2:**
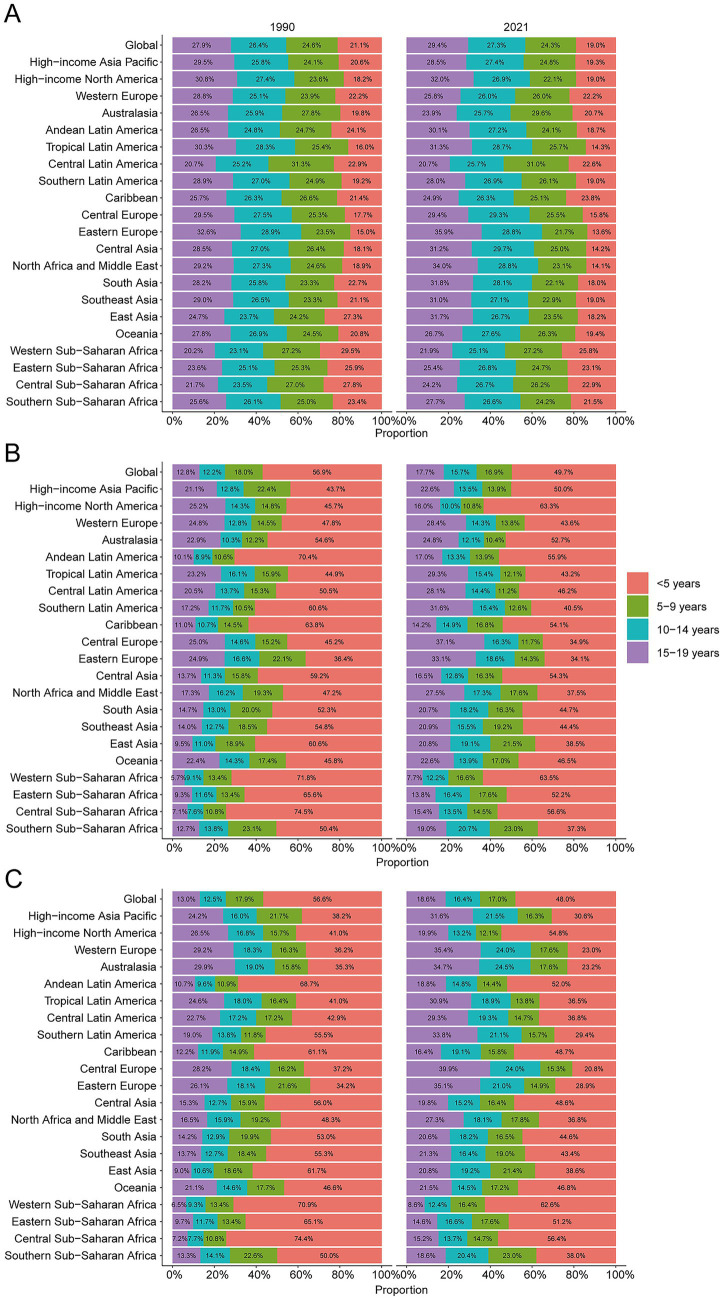
Age-specific percentages of unintentional injury incidence, mortality, and disability-adjusted life years (DALYs) in 2021. **(A)** Incidence. **(B)** Deaths. **(C)** DALYs.

A comprehensive assessment indicates a sustained decline in the global incidence of unintentional injuries among children and adolescents (0–19 years). The steepest APC occurred in 2009–2016 (−2.36, 95% CI –2.93 to −1.79). Sex-stratified analyses show comparable nadirs for boys (APC –2.52, 95% CI –2.86 to −2.18) and girls (APC –2.01, 95% CI –2.91 to −1.10) (Figure 1A). Incident cases declined from 190,707,644 (95% UI 167,142,118–216,018,713) in 1990 to 157,808,979 (95% UI 138,280,367–179,472,942) in 2021, a 17.3% decrease. The incidence rate fell by 29.1%, from 8,443.7 (95% UI 7,400.3–9,564.4) to 5,987.0 per 100,000 population (95% UI 5,246.2–6,808.9), with an EAPC of −1.21 (95% CI –1.29 to −1.12) (Table 1). Adolescents aged 15–19 years bore the highest burden in 2021 (29.4% of cases; 7,056.3 per 100,000, 95% UI 5,929.2–8,297.6), whereas children <5 years consistently exhibited the lowest rates (19.0% of cases; 4,564.1 per 100,000, 95% UI 3,983.4–5,232.0) (Figure 2A). Across all ages, boys outnumbered girls, with the greatest sex gap in the 15–19 years group (Supplementary Figure S1).

**Table 1:** Incidence of unintentional injuries between 1990 and 2021 at the global and regional level.

Location	1990 (95%UI)		2021 (95%UI)		1990–2021 (95%UI)		
	Incident cases	Incidence rate	Incident cases	Incidence rate	Cases change ^b^	Rate change ^b^	EAPC ^a^
Global	190707644.53 (167142117.97, 216018713.36)	8443.72 (7400.34, 9564.38)	157808979.36 (138280367.34, 179472942.39)	5987.04 (5246.16, 6808.94)	−17.25 (−18.59, −15.93)	−29.09 (−30.24, −27.96)	−1.21 (−1.29, −1.12)
High SDI	38329624.46 (32816479.05, 44470359.96)	15251.33 (13057.65, 17694.72)	27069223.37 (23253895.53, 31704713.72)	11631.53 (9992.10, 13623.38)	−29.38 (−30.96, −27.64)	−23.73 (−25.44, −21.86)	−0.92 (−1.05, −0.80)
High-middle SDI	41139811.83 (35622194.81, 47113170.66)	11113.89 (9623.31, 12727.59)	25950004.51 (22293294.30, 29912861.39)	8554.12 (7348.73, 9860.44)	−36.92 (−38.19, −35.29)	−23.03 (−24.58, −21.04)	−0.96 (−1.01, −0.90)
Middle SDI	53145867.34 (46624182.20, 60078752.73)	6951.23 (6098.22, 7858.02)	39935484.44 (34680074.43, 45677532.93)	5330.48 (4629.00, 6096.91)	−24.86 (−26.72, −23.27)	−23.32 (−25.22, −21.70)	−0.81 (−0.97, −0.65)
Low-middle SDI	40637882.85 (36008251.50, 45657646.13)	6875.85 (6092.52, 7725.18)	37480798.73 (33151986.18, 42152204.68)	4903.38 (4337.06, 5514.51)	−7.77 (−10.30, −5.07)	−28.69 (−30.65, −26.60)	−1.35 (−1.56, −1.13)
Low SDI	17206270.06 (15404308.70, 19159202.07)	6154.41 (5509.88, 6852.94)	27201505.19 (24485684.70, 30557429.36)	4656.15 (4191.28, 5230.59)	58.09 (54.07, 62.24)	−24.34 (−26.27, −22.36)	−0.96 (−1.10, −0.83)
Regions							
Andean Latin America	1691842.11 (1520015.48, 1895904.11)	8925.06 (8018.61, 10001.56)	1801047.40 (1580550.80, 2062227.25)	7607.84 (6676.44, 8711.10)	6.45 (2.74, 10.86)	−14.76 (−17.73, −11.23)	−0.53 (−0.59, −0.47)
Australasia	2336994.62 (1921417.42, 2770673.45)	37253.89 (30629.20, 44167.14)	2501831.05 (2040360.69, 2965729.21)	33172.94 (27054.09, 39323.98)	7.05 (2.65, 11.28)	−10.95 (−14.62, −7.44)	−0.35 (−0.45, −0.24)
Caribbean	1350616.67 (1179998.88, 1538600.24)	8945.08 (7815.09, 10190.09)	1495616.95 (1303438.61, 1710423.89)	9799.25 (8540.10, 11206.66)	10.74 (7.55, 14.32)	9.55 (6.40, 13.09)	0.25 (−0.60, 1.11)
Central Asia	4185927.86 (3649911.37, 4741019.54)	13255.05 (11557.71, 15012.79)	3436156.68 (2958413.19, 3944135.51)	9924.02 (8544.24, 11391.13)	−17.91 (−20.33, −15.72)	−25.13 (−27.33, −23.13)	−1.04 (−1.13, −0.95)
Central Europe	9426193.05 (7847880.99, 11068587.74)	24004.54 (19985.24, 28187.02)	4375515.74 (3550168.87, 5187054.72)	18573.99 (15070.41, 22018.96)	−53.58 (−55.09, −52.10)	−22.62 (−25.13, −20.15)	−0.84 (−0.96, −0.72)
Central Latin America	13517534.71 (11567718.98, 15561866.87)	16358.86 (13999.20, 18832.90)	9735769.63 (8220264.42, 11391961.09)	11415.57 (9638.58, 13357.52)	−27.98 (−30.02, −26.18)	−30.22 (−32.20, −28.48)	−0.44 (−0.85, −0.02)
Central Sub-Saharan Africa	1572992.35 (1409852.63, 1778930.51)	5076.10 (4549.65, 5740.67)	2908183.83 (2584761.07, 3286586.68)	3953.44 (3513.78, 4467.85)	84.88 (77.97, 91.93)	−22.12 (−25.03, −19.15)	−0.83 (−0.95, −0.71)
East Asia	19952267.60 (17102806.60, 22952540.44)	4336.13 (3716.87, 4988.17)	12964316.28 (10976368.30, 15047324.76)	3758.30 (3182.00, 4362.16)	−35.02 (−38.05, −32.24)	−13.33 (−17.37, −9.62)	−1.11 (−1.59, −0.63)
Eastern Europe	13240453.47 (11325980.59, 15350717.45)	19680.98 (16835.25, 22817.73)	5836168.79 (5000698.00, 6779993.45)	12643.46 (10833.49, 14688.15)	−55.92 (−57.41, −54.57)	−35.76 (−37.93, −33.79)	−1.93 (−2.16, −1.69)
Eastern Sub-Saharan Africa	6569407.25 (5892094.18, 7417473.75)	5923.86 (5313.10, 6688.59)	10354221.15 (9299366.60, 11765590.10)	4549.61 (4086.11, 5169.76)	57.61 (54.01, 61.46)	−23.20 (−24.95, −21.33)	−0.90 (−0.98, −0.82)
High-income Asia Pacific	8247648.03 (7076334.20, 9564410.63)	16388.21 (14060.79, 19004.64)	3842849.86 (3250510.21, 4536931.35)	12481.24 (10557.37, 14735.55)	−53.41 (−54.85, −52.07)	−23.84 (−26.20, −21.65)	−0.98 (−1.08, −0.88)
High-income North America	10439895.75 (8903497.04, 12172112.82)	12773.67 (10893.82, 14893.11)	7023817.01 (5990098.67, 8212891.71)	7842.76 (6688.51, 9170.47)	−32.72 (−35.44, −29.11)	−38.60 (−41.08, −35.30)	−1.81 (−2.50, −1.11)
North Africa and Middle East	14204928.20 (12687000.63, 15893445.91)	8035.72 (7177.03, 8990.92)	13766506.21 (12170460.00, 15551646.27)	5821.14 (5146.25, 6575.98)	−3.09 (−6.57, 0.35)	−27.56 (−30.17, −24.99)	−1.02 (−1.14, −0.89)
Oceania	110761.50 (97399.60, 125869.92)	3290.22 (2893.30, 3739.02)	214691.91 (191760.96, 239866.18)	3361.72 (3002.65, 3755.90)	93.83 (84.13, 103.57)	2.17 (−2.94, 7.31)	−0.44 (−1.31, 0.44)
South Asia	35214442.53 (30912843.01, 39984328.33)	6492.00 (5698.97, 7371.36)	31156417.35 (27333106.75, 35065862.03)	4558.40 (3999.02, 5130.38)	−11.52 (−15.36, −7.70)	−29.78 (−32.83, −26.75)	−1.44 (−1.65, −1.24)
Southeast Asia	12009304.75 (10641416.47, 13535563.15)	5461.23 (4839.18, 6155.29)	9540644.56 (8437599.46, 10772040.97)	4161.29 (3680.18, 4698.38)	−20.56 (−22.44, −18.79)	−23.80 (−25.61, −22.11)	−0.72 (−1.12, −0.33)
Southern Latin America	4454196.71 (3784459.27, 5191060.98)	22984.29 (19528.35, 26786.62)	4303935.83 (3628944.49, 5001335.45)	22060.56 (18600.78, 25635.20)	−3.37 (−7.00, 1.04)	−4.02 (−7.63, 0.37)	0.06 (−0.20, 0.32)
Southern Sub-Saharan Africa	1298536.80 (1145106.14, 1489401.04)	4907.42 (4327.58, 5628.73)	1171842.81 (1040664.13, 1339553.27)	3748.16 (3328.58, 4284.59)	−9.76 (−11.81, −7.36)	−23.62 (−25.36, −21.59)	−0.92 (−1.09, −0.75)
Tropical Latin America	7639558.66 (6541174.82, 8873940.90)	11029.99 (9444.14, 12812.19)	5056700.67 (4355234.52, 5894155.65)	7594.07 (6540.62, 8851.74)	−33.81 (−36.32, −31.44)	−31.15 (−33.77, −28.69)	−1.49 (−1.80, −1.18)
Western Europe	17226594.95 (14845494.93, 20080597.69)	17516.39 (15095.23, 20418.40)	14050820.18 (11866361.19, 16688221.15)	15320.79 (12938.89, 18196.57)	−18.44 (−21.28, −15.45)	−12.53 (−15.59, −9.34)	−0.38 (−0.48, −0.29)
Western Sub-Saharan Africa	6017546.96 (5361432.21, 6770990.87)	5598.02 (4987.65, 6298.93)	12271925.46 (10984644.30, 13757475.63)	4569.25 (4089.96, 5122.37)	103.94 (98.66, 109.82)	−18.38 (−20.49, −16.02)	−0.69 (−0.77, −0.60)

EAPC, estimated annual percentage change; SDI, Sociodemographic Index; UI, uncertainty interval. ^a^EAPC is expressed as 95% confidence interval. ^b^Change shows the percentage change.

#### Mortality

Mirroring incidence, mortality declined globally. The lowest APC was recorded in 2009–2013 (−6.03, 95% CI –10.79 to −1.01). For boys, the nadir extended from 2009 to 2021 (APC –4.65, 95% CI –5.29 to −4.00), whereas girls showed the sharpest drop in 2010–2012 (APC –9.74, 95% CI –28.55 to 14.01) (Figure 1B). Deaths decreased from 793,639 (95% UI 708,525–883,500) in 1990 to 305,596 (95% UI 240,004–365,146) in 2021 (−61.5%). The mortality rate fell by 67.0%, from 35.1 (95% UI 31.4–39.1) to 11.6 per 100,000 (95% UI 9.1–13.9), yielding an EAPC of −3.34 (95% CI –3.60 to −3.08) (Table 2). Children <5 years accounted for 49.7% of deaths in 2021 (23.1 per 100,000, 95% UI 16.4–29.7); adolescents 15–19 years sustained the lowest mortality (8.23 per 100,000, 95% UI 7.08–9.08) (Figure 2B). Male mortality exceeded female mortality across nearly all ages, especially in late adolescence (Supplementary Figure S1).

**Table 2:** Mortality of unintentional injuries between 1990 and 2021 at the global and regional level.

Location	1990 (95%UI)		2021 (95%UI)		1990–2021 (95%UI)		
	Death cases	Death rate	Death cases	Death rate	Cases change ^b^	Rate change ^b^	EAPC ^a^
Global	793638.99 (708524.74, 883499.50)	35.14 (31.37, 39.12)	305595.85 (240003.52, 365145.51)	11.59 (9.11, 13.85)	−61.49 (−68.75, −53.47)	−67.01 (−73.23, −60.13)	−3.34 (−3.60, −3.08)
High SDI	21364.49 (20519.25, 22271.97)	26.71 (24.16, 29.66)	6568.83 (6110.66, 6956.52)	5.64 (5.10, 6.22)	−69.25 (−70.96, −67.48)	−66.80 (−68.64, −64.88)	−3.26 (−3.46, −3.06)
High-middle SDI	98862.25 (89435.89, 109773.25)	8.50 (8.16, 8.86)	17113.09 (15466.11, 18854.40)	2.82 (2.63, 2.99)	−82.69 (−84.85, −80.48)	−78.88 (−81.51, −76.19)	−5.07 (−5.27, −4.86)
Middle SDI	304570.94 (277478.19, 335957.79)	38.57 (33.03, 43.95)	65394.88 (56551.19, 74353.79)	12.63 (10.12, 15.07)	−78.53 (−81.51, −75.36)	−78.09 (−81.13, −74.86)	−4.48 (−4.82, −4.13)
Low-middle SDI	227950.02 (195244.45, 259772.68)	50.22 (40.42, 59.47)	96547.42 (77383.30, 115181.36)	20.48 (14.45, 26.24)	−57.65 (−65.48, −47.12)	−67.25 (−73.31, −59.11)	−3.56 (−3.94, −3.18)
Low SDI	140401.39 (112997.53, 166268.00)	39.84 (36.29, 43.94)	119674.07 (84389.02, 153277.89)	8.73 (7.55, 9.92)	−14.76 (−37.38, 14.87)	−59.21 (−70.03, −45.03)	−2.65 (−3.11, −2.20)
Regions							
Andean Latin America	9452.94 (7682.38, 10561.82)	49.87 (40.53, 55.72)	2809.59 (2252.72, 3533.49)	11.87 (9.52, 14.93)	−70.28 (−76.65, −62.18)	−76.20 (−81.30, −69.72)	−4.59 (−4.85, −4.34)
Australasia	364.73 (350.93, 379.37)	5.81 (5.59, 6.05)	149.79 (134.87, 166.92)	1.99 (1.79, 2.21)	−58.93 (−63.53, −54.05)	−65.84 (−69.66, −61.78)	−2.72 (−3.03, −2.41)
Caribbean	5310.26 (4432.74, 6220.57)	35.17 (29.36, 41.20)	4222.65 (3550.98, 4930.27)	27.67 (23.27, 32.30)	−20.48 (−32.69, −5.96)	−21.33 (−33.41, −6.96)	−0.91 (−3.48, 1.73)
Central Asia	10937.07 (10104.62, 11835.93)	34.63 (32.00, 37.48)	4197.87 (3590.21, 4985.76)	12.12 (10.37, 14.40)	−61.62 (−67.41, −54.43)	−64.99 (−70.27, −58.44)	−3.53 (−3.67, −3.39)
Central Europe	5132.46 (4944.90, 5284.82)	13.07 (12.59, 13.46)	630.41 (562.95, 684.29)	2.68 (2.39, 2.90)	−87.72 (−89.06, −86.49)	−79.53 (−81.76, −77.48)	−5.01 (−5.16, −4.85)
Central Latin America	17532.38 (16563.88, 18703.92)	21.22 (20.05, 22.64)	5632.78 (4660.47, 6839.60)	6.60 (5.46, 8.02)	−67.87 (−73.67, −61.03)	−68.87 (−74.49, −62.25)	−3.64 (−4.29, −2.99)
Central Sub-Saharan Africa	18387.20 (14599.52, 22407.25)	59.34 (47.11, 72.31)	12877.67 (8702.88, 17642.78)	17.51 (11.83, 23.98)	−29.96 (−48.75, −1.78)	−70.50 (−78.41, −58.62)	−3.53 (−3.86, −3.20)
East Asia	228948.67 (200209.79, 263024.81)	49.76 (43.51, 57.16)	30915.28 (26845.07, 35545.06)	8.96 (7.78, 10.30)	−86.50 (−88.83, −83.96)	−81.99 (−85.10, −78.60)	−5.33 (−5.59, −5.07)
Eastern Europe	15209.92 (14715.97, 15708.74)	22.61 (21.87, 23.35)	2850.64 (2687.76, 2978.44)	6.18 (5.82, 6.45)	−81.26 (−82.29, −80.36)	−72.68 (−74.20, −71.37)	−4.55 (−5.05, −4.05)
Eastern Sub-Saharan Africa	47963.80 (38479.58, 56688.19)	43.25 (34.70, 51.12)	35159.57 (25283.79, 48424.94)	15.45 (11.11, 21.28)	−26.70 (−45.46, 3.69)	−64.28 (−73.42, −49.47)	−3.12 (−3.21, −3.03)
High-income Asia Pacific	4155.17 (3795.33, 4472.47)	8.26 (7.54, 8.89)	530.38 (494.55, 575.14)	1.72 (1.61, 1.87)	−87.24 (−88.40, −85.53)	−79.14 (−81.04, −76.35)	−4.93 (−5.70, −4.16)
High-income North America	6182.80 (6095.99, 6270.90)	7.56 (7.46, 7.67)	3480.33 (3188.18, 3795.23)	3.89 (3.56, 4.24)	−43.71 (−48.55, −38.81)	−48.63 (−53.05, −44.16)	−1.77 (−1.98, −1.56)
North Africa and Middle East	79885.58 (70269.17, 89660.21)	45.19 (39.75, 50.72)	22381.52 (18416.73, 26442.06)	9.46 (7.79, 11.18)	−71.98 (−76.90, −66.13)	−79.06 (−82.74, −74.68)	−3.81 (−4.15, −3.47)
Oceania	748.26 (569.40, 947.47)	22.23 (16.91, 28.15)	1261.92 (965.89, 1626.24)	19.76 (15.12, 25.46)	68.65 (33.26, 113.39)	−11.10 (−29.76, 12.48)	−0.56 (−1.25, 0.15)
South Asia	204998.92 (169954.98, 240694.65)	37.79 (31.33, 44.37)	81664.70 (64260.37, 97984.85)	11.95 (9.40, 14.34)	−60.16 (−68.00, −49.12)	−68.39 (−74.60, −59.62)	−3.74 (−4.07, −3.41)
Southeast Asia	66520.70 (57045.56, 76147.60)	30.25 (25.94, 34.63)	25309.46 (21495.02, 29416.82)	11.04 (9.38, 12.83)	−61.95 (−68.00, −54.30)	−63.51 (−69.31, −56.17)	−2.98 (−4.00, −1.96)
Southern Latin America	4045.98 (3920.91, 4173.87)	20.88 (20.23, 21.54)	900.66 (809.33, 991.08)	4.62 (4.15, 5.08)	−77.74 (−80.01, −75.38)	−77.89 (−80.14, −75.54)	−4.57 (−4.78, −4.35)
Southern Sub-Saharan Africa	5409.29 (4562.21, 6179.40)	20.44 (17.24, 23.35)	4558.74 (3783.80, 5443.48)	14.58 (12.10, 17.41)	−15.72 (−29.90, 0.40)	−28.67 (−40.67, −15.03)	−0.56 (−0.86, −0.26)
Tropical Latin America	11163.06 (10208.99, 12092.74)	16.12 (14.74, 17.46)	3814.03 (3264.73, 4361.13)	5.73 (4.90, 6.55)	−65.83 (−70.89, −59.93)	−64.46 (−69.72, −58.32)	−2.82 (−3.05, −2.59)
Western Europe	5124.71 (5032.04, 5218.56)	5.21 (5.12, 5.31)	1393.90 (1298.38, 1488.55)	1.52 (1.42, 1.62)	−72.80 (−74.74, −70.95)	−70.83 (−72.92, −68.85)	−3.67 (−3.89, −3.44)
Western Sub-Saharan Africa	46165.10 (37400.82, 54427.46)	42.95 (34.79, 50.63)	60853.95 (36219.24, 78158.31)	22.66 (13.49, 29.10)	31.82 (−17.98, 78.54)	−47.24 (−67.17, −28.54)	−1.76 (−1.97, −1.55)

EAPC, estimated annual percentage change; SDI, Sociodemographic Index; UI, uncertainty interval. ^a^EAPC is expressed as 95% confidence interval. ^b^Change shows the percentage change.

#### DALYs

DALYs also fell. The sharpest APC was recorded in 2010–2013 (−5.43, 95% CI –12.34 to 2.04) (Figure 1C). DALYs decreased from 70,883,769 (95% UI 63,582,671–78,251,186) in 1990 to 28,410,167 (95% UI 23,100,433–33,513,238) in 2021 (−59.9%). The DALY rate dropped by 65.7%, from 3,138.4 (95% UI 2,815.2–3,464.6) to 1,077.8 per 100,000 (95% UI 876.4–1,271.4), with an EAPC of −3.23 (95% CI –3.48 to −2.99) (Supplementary Table S1). Children <5 years contributed 48% of DALYs in 2021 (2,072.0 per 100,000, 95% UI 1,481.1–2,651.7); adolescents 15–19 years showed the lowest rates (803.9 per 100,000, 95% UI 709.6–908.1) (Figure 2C). Boys accrued more DALYs than girls across all ages (Supplementary Figure S1).

### Regional trends by SDI

**Figure 3:**
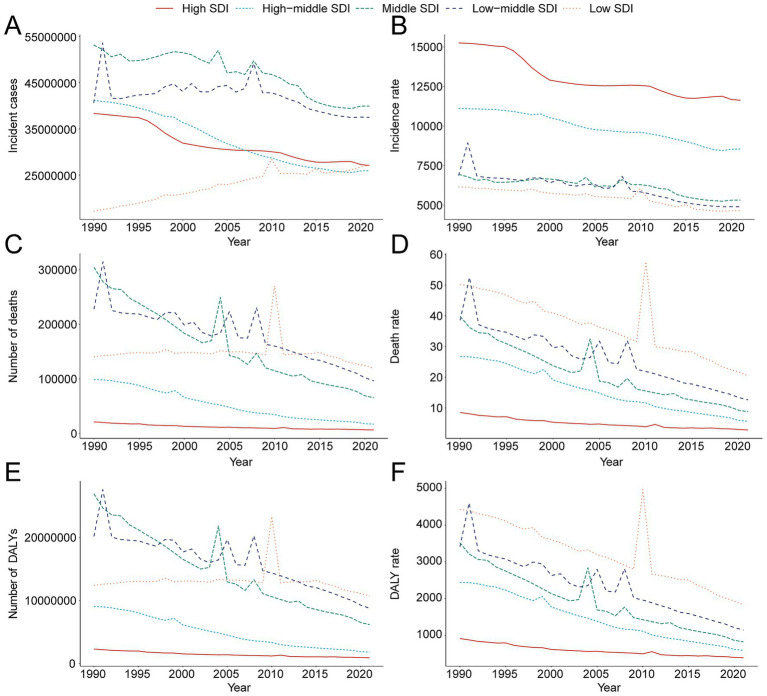
Epidemiologic trends of incidence, death, and disability-adjusted Life-Years (DALYs) rates and cases in 5 sociodemographic index (SDI) regions of unintentional injury from 1990 to 2021. **(A)** Trends in incident cases. **(B)** Trends in incidence rate. **(C)** Trends in death cases. **(D)** Trends in death rate. **(E)** Trends in DALY cases. **(F)** Trends in DALY rate.

Between 1990 and 2021, incidence, mortality, and DALY rates fell across all SDI strata. In 2021, middle-SDI regions recorded the largest absolute numbers of cases (39,935,484.44; 95% UI 34,680,074.43–45,677,532.93), deaths (119,674.07; 95% UI 84,389.02–153,277.89), and DALYs (10,712,770.20; 95% UI 7,729,199.47–13,508,499.90) (Tables 1, 2, Supplementary Table S1). The greatest decline in incidence occurred in low-middle-SDI regions; high-middle-SDI regions showed the steepest mortality reduction, and middle-SDI regions the sharpest DALY decline (Figures 3A–F).

### Geographical patterns

#### Incidence

**Figure 4:**
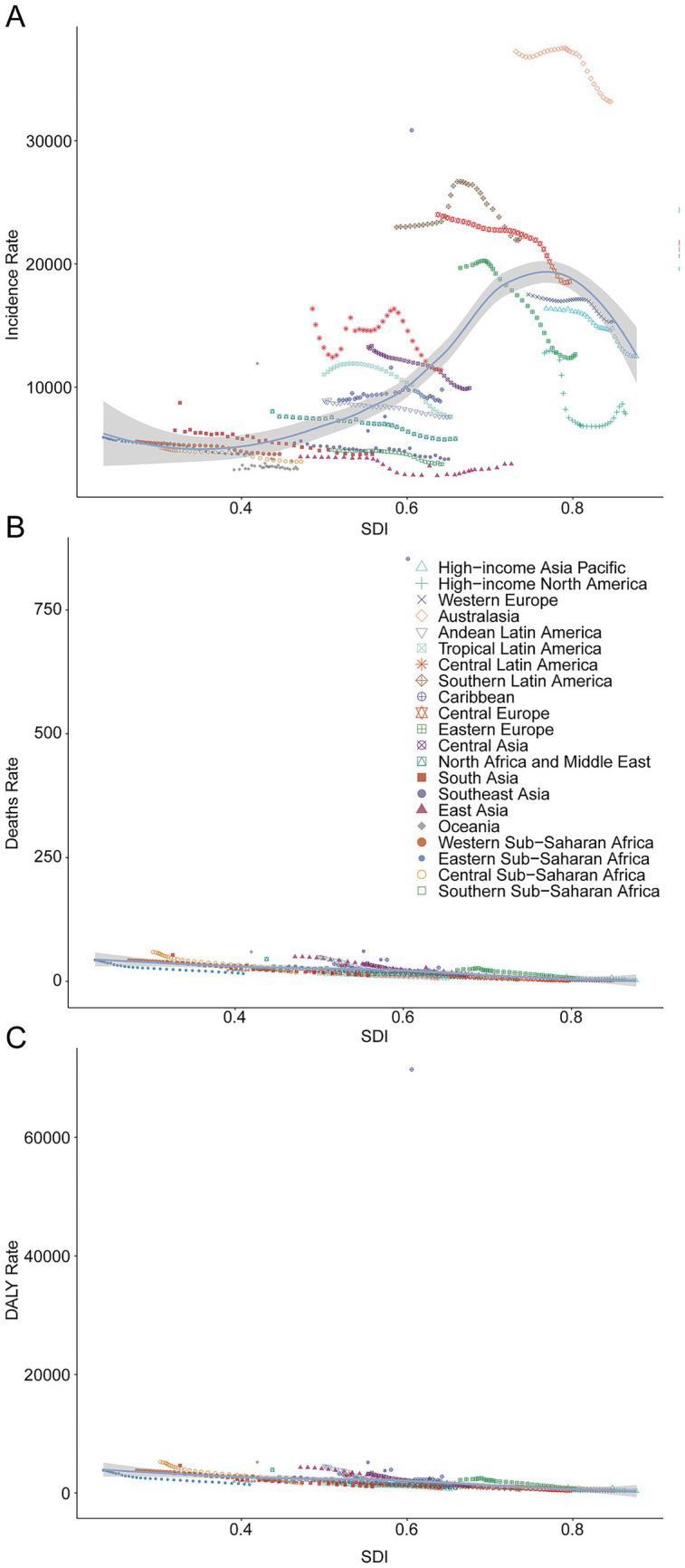
Incidence, death, and disability-adjusted life-years (DALYs) rates for unintentional injury from 1990 to 2021. **(A)** Incidence rate. **(B)** Death rate. **(C)** DALYs rate.

In 2021, South Asia reported the most cases (31,156,417.35; 95% UI 27,333,106.75–35,065,862.03), whereas Oceania had the fewest (214,691.91; 95% UI 191,760.96–239,866.18). Incidence rates peaked in Australasia at 33,172.94 per 100,000 (95% UI 27,054.09–39,323.98) and were lowest in Oceania at 3,361.72 per 100,000 (95% UI 3,002.65–3,755.90). From 1990 to 2021, the Caribbean registered the largest relative increase (EAPC 0.25, 95% CI –0.60 to 1.11), while Eastern Europe recorded the greatest decline (EAPC –1.93, 95% CI –2.16 to −1.69) (Table 1). Twelve of 21 regions exceeded the 2021 global mean incidence (5,987.04; 95% UI 5,246.16–6,808.94) (Figure 4A).

#### Mortality

South Asia led in deaths (81,664.70; 95% UI 64,260.37–97,984.85). The Caribbean exhibited the highest mortality rate (27.67 per 100,000, 95% UI 23.27–32.30). East Asia posted the steepest decline (EAPC –5.33, 95% CI –5.59 to −5.07), whereas Oceania declined least (EAPC –0.56, 95% CI –1.25 to 0.15) (Figure 4B).

#### DALYs

South Asia accrued the most DALYs (7,322,999.41; 95% UI 5,922,433.11–8,728,555.14). Conversely, the Caribbean had the highest DALY rate (2,761.53 per 100,000, 95% UI 2,365.79–3,226.20). East Asia achieved the greatest DALY decline (EAPC –5.26, 95% CI –5.50 to −5.02), whereas the Caribbean changed least (EAPC –0.34, 95% CI –2.78 to 2.16) (Figure 4C).

### National trends

#### Incidence

**Figure 5:**
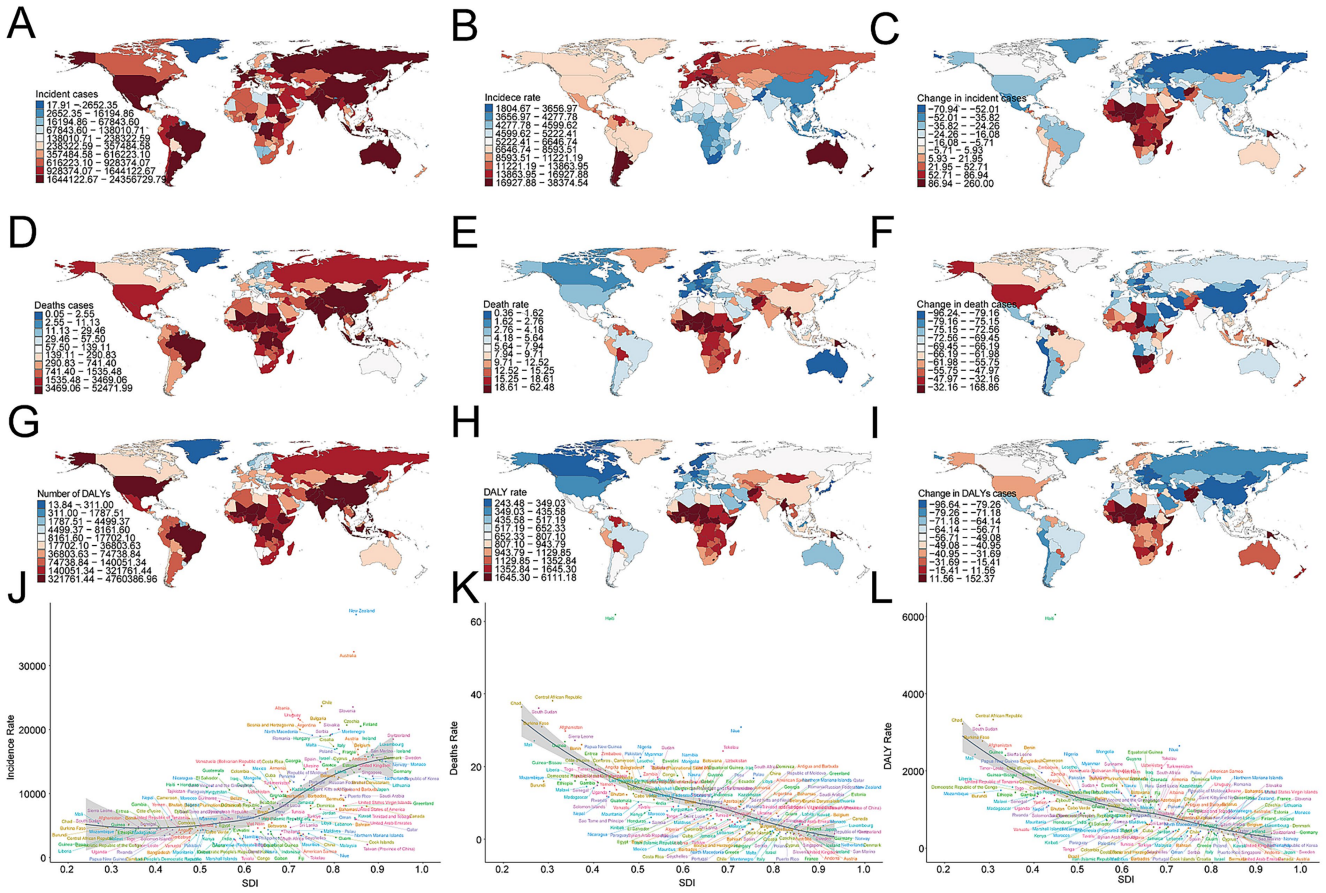
Incidence, death, and disability-adjusted life-years (DALYs) of unintentional injury across 204 countries and territories. **(A)** Incident cases. **(B)** Incidence rate. **(C)** Change in incident cases. **(D)** Death cases. **(E)** Death rate. **(F)** Change in death cases. **(G)** DALY cases. **(H)** DALY rate. **(I)** Change in DALY cases. **(J)** Trends in incidence rate. **(K)** Trends in death rate. **(L)** Trends in DALY rate.

In 2021, India recorded the largest number of childhood and adolescent unintentional-injury cases (24,115,574.05; 95% UI 21,137,413.69–27,296,090.99), reflecting an 18.22% decline since 1990 (95% UI –22.19% to −14.31%). New Zealand posted the highest incidence rate globally (37,994.60 per 100,000; 95% UI 32,454.34–43,392.47). The steepest reduction occurred in the Russian Federation (EAPC –2.26; 95% CI –2.54 to −1.97), whereas Chile exhibited the greatest increase (EAPC 0.91; 95% CI 0.80–1.02) (Supplementary Table S2; Figures 5A–C). The 2021 global incidence rate was 5,987.04 per 100,000 (95% UI 5,246.16–6,808.94), with 109 countries exceeding and 95 falling below this benchmark (Figure 5J).

#### Mortality

2India also accounted for the highest number of deaths (51,952.47; 95% UI 38,458.49–65,677.49), a 60.99% decrease from 1990 (95% UI –70.14% to −48.12%). Haiti registered the greatest mortality rate (61.86 per 100,000; 95% UI 50.86–73.32). The Republic of Korea achieved the sharpest decline (EAPC –7.21; 95% CI –7.59 to −6.83), while Zimbabwe showed the largest rise (EAPC 2.41; 95% CI 1.79–3.03) (Supplementary Table S3; Figures 5D–F). The global mortality rate was 11.59 per 100,000 (95% UI 9.11–13.85), with 68 countries above and 136 below the mean (Figure 5K).

#### DALYs

India bore the heaviest disability burden, accruing 4,713,254.42 DALYs (95% UI 3,642,013.51–5,937,671.10), down 59.76% since 1990 (95% UI –68.43% to −47.11%). Haiti experienced the highest DALY rate (6,050.67 per 100,000; 95% UI 5,017.71–7,162.70). Equatorial Guinea realized the steepest decrease (EAPC –6.17; 95% CI –6.61 to −5.72), whereas Zimbabwe posted the greatest increase (EAPC 2.19; 95% CI 1.61–2.77) (Supplementary Table S4; Figures 5G–I). The global DALY rate was 1,077.84 per 100,000 (95% CI 876.40–1,271.44); 69 countries exceeded this value, and 135 remained below it (Figure 5L).

### Factors influencing EAPCs

**Figure 6:**
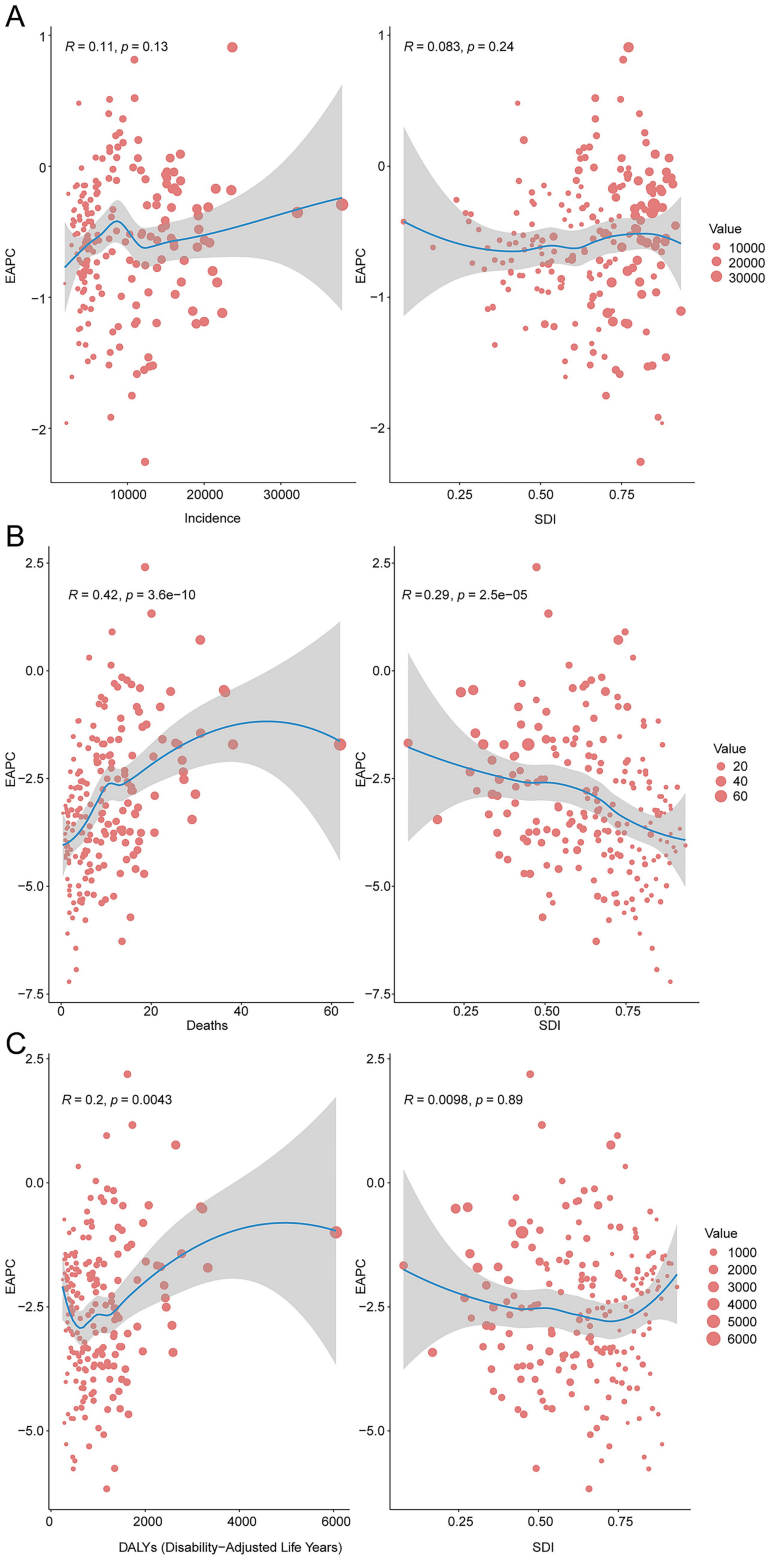
The correlation between EAPC and unintentional injury incidence rate, death rate, and DALY rate in 2021. **(A)**, Incidence rate. **(B)**, Mortality rate. **(C)**, DALY rate.

Across 204 countries and territories, the EAPC in incidence displayed no significant linear relation with baseline case numbers (*R* = 0.11, *p* = 0.13) or with the Socio-demographic Index (SDI; *R* = −0.083, *p* = 0.24) (Figure 6A). By contrast, the EAPC in mortality correlated positively with baseline deaths (*R* = 0.42, *p* = 3.6 × 10^−10^) and inversely with SDI (*R* = −0.29, *p* = 2.5 × 10^−5^) (Figure 6B). For DALYs, a modest positive correlation emerged with baseline DALYs (*R* = 0.20, *p* = 0.0043). In contrast, the association with SDI was negligible (*R* = 0.0098, *p* = 0.89) (Figure 6C).

### Attributable risk factors

**Figure 7:**
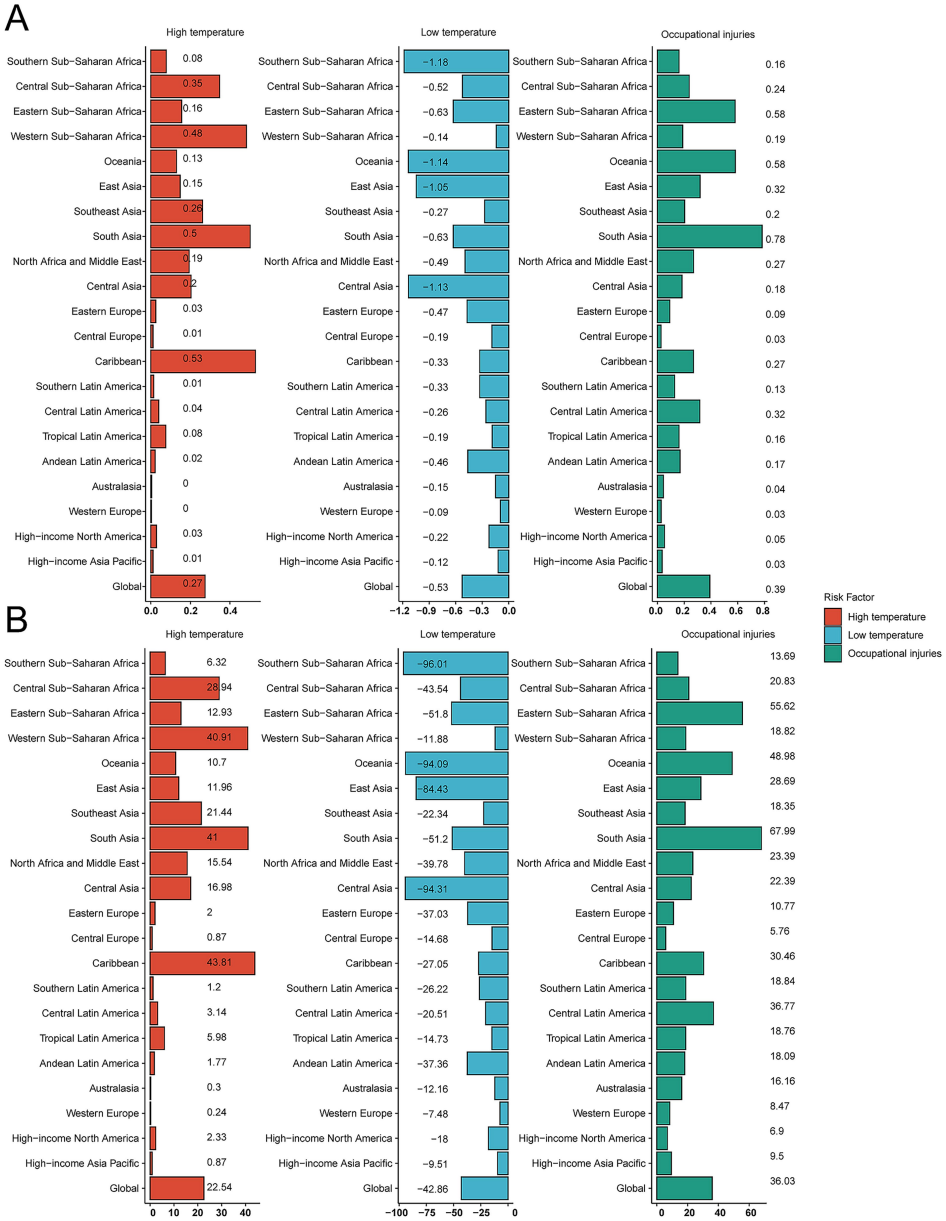
Proportion of unintentional injury deaths and disability-adjusted life-years (DALYs) attributable to risk factors. **(A)** Death rate. **(B)** DALY rate.

The GBD framework identified three principal risk factors for unintentional injuries in children and adolescents: occupational injuries, low temperature, and high temperature. Occupational injuries accounted for a global mortality rate of 0.39 per 100,000 population (95% UI 0.34–0.44). High temperature contributed 22.54 DALYs per 100,000 globally (95% UI 10.10–32.27); the Caribbean recorded the highest regional burden at 43.81 DALYs per 100,000 (95% UI 26.31–61.99) (Figures 7A,B).

### Projections to 2035

**Figure 8:**
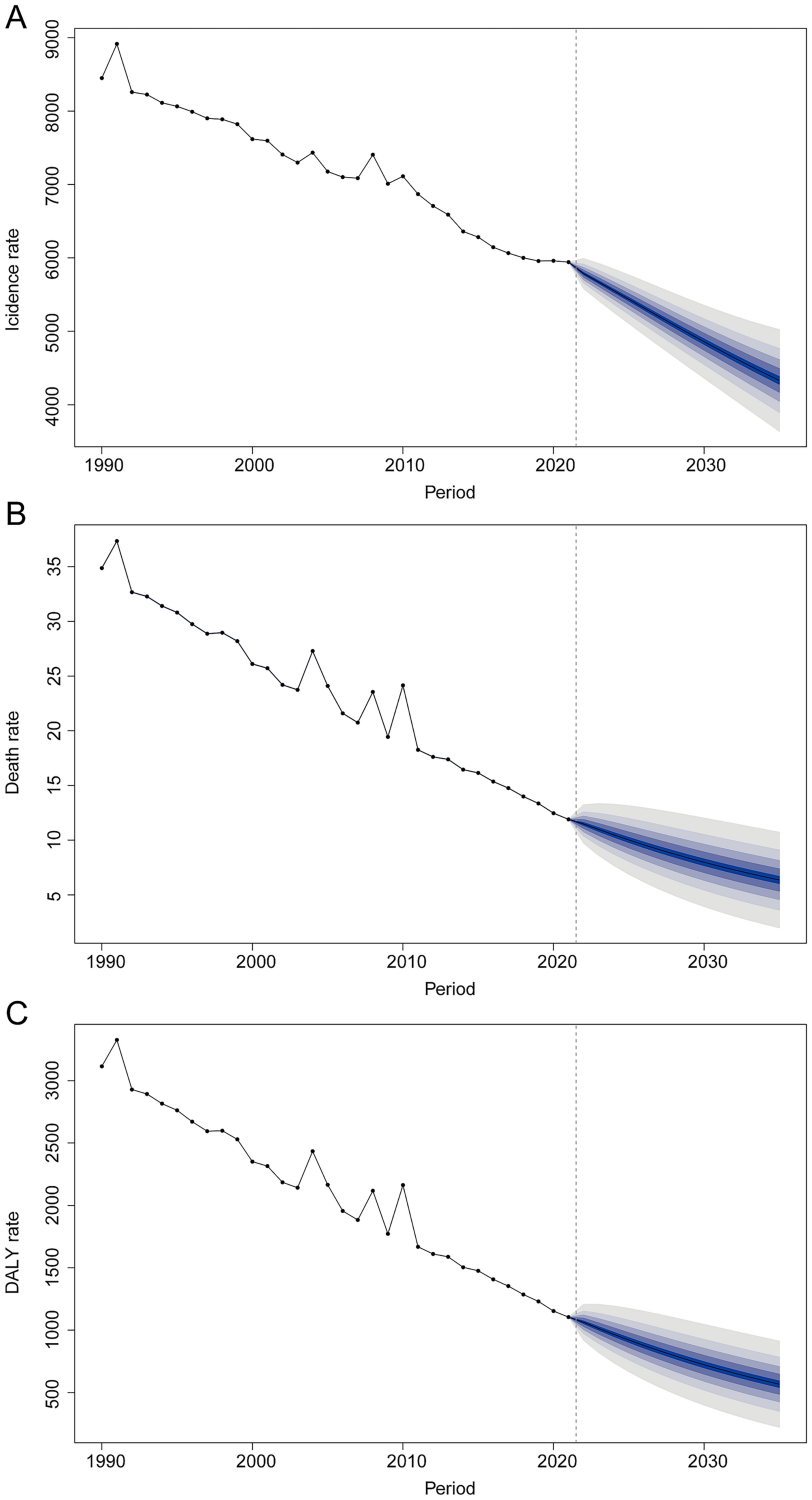
Bayesian age–period–cohort (BAPC) projections of unintentional injury burden to 2035. **(A)** Incidence rate; **(B)** mortality rate; **(C)** DALY rate.

BAPC modeling suggests a continued decline in burden through 2035, with incidence, mortality, and DALY rates projected at 4,331 (95% CI 3,505.71–5,156.29), 6.36 (95% CI 1.15–11.57) and 566.43 per 100,000 population (95% CI 154.60–978.26), respectively (Figures 8A–C).

## Discussion

In this study, we found that the global burden of unintentional injuries in children and adolescents has declined substantially over the past three decades. From 1990 to 2021, Incidence, mortality, and DALY rates for unintentional injuries all showed downward trends worldwide. Notably, the decline in mortality outpaced the decline in incidence, suggesting improvements in trauma care and injury survival. By 2019, the global age-standardized mortality rate for injuries under 20 years old had fallen to roughly 23 per 100,000 (10), and our 2021 estimates indicate further reductions. This progress reflects significant global efforts in injury prevention and health system response. However, the gains have been uneven. Males consistently experienced higher injury rates than females, and while both sexes saw mortality reductions, the gap between boys and girls persists. We also observed distinct age patterns: adolescents 15–19 years had the highest incidence of injuries, yet children under 5 years suffered the highest rates of injury mortality and long-term disability. This implies that young children, though less frequently injured, are especially vulnerable to fatal or life-altering outcomes when injuries occur, likely due to frailty and supervision gaps (21). Meanwhile, older adolescents engage in more high-risk activities, driving up injury incidence (22). These sex and age disparities in injury burden have remained remarkably consistent over time, highlighting the need for tailored interventions (for example, adolescent-targeted safety programs and enhanced protection for preschool-age children).

Our analysis underscores that global improvements mask stark regional and socioeconomic inequalities. Children in low-SDI regions continue to face a much higher risk of injury death and disability than those in high-SDI regions (1). This pattern suggests that children in poorer regions are not inherently injured more often, but their injuries are far more likely to be fatal or disabling – a reflection of underlying disparities in safety infrastructure and medical care. High-SDI settings tend to have extensive injury prevention measures (e.g., safer roads, fire safety, poison control) and robust emergency care systems that improve survival, whereas many low-SDI countries lack these life-saving advantages (23–25). The result is a preventable inequity: a child in a low-income country is several times more likely to die from the same injury than a child in a high-income country. This inequality is also evident on a regional level (26). For example, in 2017, the Eastern Mediterranean Region had an injury mortality rate of 43.2 per 100,000 – well above the global average of 27.2 – largely due to a combination of road traffic crashes and other hazards in contexts of conflict and limited resources (8). Sub-Saharan Africa and South Asia similarly remain regions of high concern, contributing a large share of global child injury deaths each year. By contrast, high-income regions (e.g., Western Europe, North America) have among the lowest injury rates and showed the steepest declines over the study period, owing to long-standing investments in injury prevention. These findings highlight that despite overall progress, a persistent “injury gap” remains between higher- and lower-SDI parts of the world. Children in certain countries, notably those in parts of sub-Saharan Africa, South Asia, and conflict-affected Middle Eastern nations, still bear an unacceptably high burden of unintentional injuries. Tackling this disparity should be a global priority.

Beyond macroeconomic development captured by SDI, socio-cultural contexts shape exposure, supervision, and care-seeking. Gender norms often lead to earlier engagement of boys in risk-prone activities (traffic, heights, open water), higher sensation-seeking, and greater tolerance of risk, aligning with the persistent male excess we observed across ages. Family structure and caregiver availability influence supervision quality; single-caregiver or skip-generation households and caregiver labor demands (e.g., agriculture, informal work) may increase unsupervised time for younger children, elevating drowning and burn risks.

Educational attainment and safety literacy affect hazard recognition and protective behaviors (helmet use, safe storage of fuels/chemicals). Urbanization changes risk profiles: while dense traffic elevates RTI risk, urban areas may provide faster emergency response; conversely, rural settings often combine water exposure, solid-fuel use, and longer transport times to definitive care. Cultural practices (e.g., cooking at floor level; seasonal migration of caregivers) and child labor also contribute. Integrating injury prevention into school curricula, community childcare solutions, and context-specific behavior-change programs can address these drivers and complement system-level interventions.

Mechanism-specific patterns underscore the need for tailored prevention. For road injuries, proven measures include graduated driver licensing and helmet/seatbelt/child-restraint enforcement, speed management, safer road design, and post-crash care strengthening. Drowning prevention should prioritize barriers and supervised safe play near water, community-based swim and rescue training, and childcare options in settings where caregivers’ workload limits supervision. Falls prevention requires safer built environments (window guards, stair gates, playground surfacing) and sports safety programs for adolescents. Fire/burn prevention is linked to clean cooking transitions, safer stoves and fuels, and household fire-safety education. For poisoning, child-resistant packaging and safe storage are critical. Aligning investments with the dominant local mechanism mix can accelerate declines beyond those projected from recent trends alone.

Several factors likely explain the observed declines and differences in unintentional injury burden. Improvements in health systems and trauma care have played a key role in reducing mortality (27). Over 30 years, many countries expanded emergency medical services, trauma centers, and surgical care for injuries, leading to better outcomes for injured children. This is evidenced by the faster fall in death rates compared to incidence rates – injuries may not be dramatically less frequent in some settings, but children who are injured have a better chance of survival today than in 1990. Advanced trauma care and prompt treatment of severe injuries (such as hemorrhage control, ventilatory support, and infection management) have particularly benefited high- and middle-SDI regions (28). In low-SDI regions, however, such care is often less accessible, contributing to higher case fatality and DALY rates (29).

At the same time, safety regulations and preventive measures have helped curb injury incidence and severity, especially in higher-SDI countries (30). Over the past decades, many nations enacted and enforced laws mandating child safety restraints in cars, motorcycle helmets, seat-belt use, safe storage of toxic substances, and fencing of pools or wells (31). Engineering improvements (such as safer road design, traffic calming, and flame-retardant materials in homes) have reduced exposure to hazards. Public health campaigns have raised awareness about supervising children around water, using smoke alarms, and other injury prevention behaviors. Collectively, these interventions have led to marked declines in certain injury types, reflecting efforts in countries like China to implement community water safety programs. Road traffic injury mortality among children has also decreased in many regions due to better road safety legislation and vehicle safety standards, although the extent varies. High-income countries saw dramatic reductions in child passenger fatalities with the adoption of car seats and seat-belt laws, whereas some low-income countries, undergoing rapid motorization without equivalent safety measures, struggled to reduce road deaths (32). The net effect worldwide has been a gradual downward trend in transport-related deathspmc.ncbi.nlm.nih.gov. In short, where strong safety policies and environmental protections have been put in place, injury rates have fallen, demonstrating that unintentional injuries are largely preventable with known interventions.

Differences in environmental exposures and societal factors also contribute to regional trends. Children in poorer rural areas may be more exposed to hazards like open water (leading to drowning) or cooking fires (burn injuries) due to a lack of protective infrastructure (33). In contrast, urbanization and industrial development bring other risks (traffic, high-rise falls) but can also facilitate better emergency response. Cultural and behavioral factors (such as risk-taking tendencies among adolescent males or traditional cooking practices that raise burn risk) influence injury patterns as well. For instance, the slower decline in adolescent male injury mortality observed in our study is consistent with risk behaviors (speeding, alcohol use, etc.) that remain challenging to change. Many low-SDI countries have youthful populations engaged in labor or chores that expose them to injuries (e.g., farming, fetching water, caring for siblings around hazards), whereas high-SDI countries have largely mitigated such risks (34). Additionally, climate and environmental changes may be affecting injury trends: more frequent floods can increase drowning incidents, and extreme heat or wildfires can raise burn and heat-related injuries, potentially offsetting some gains in certain regions. These complex factors mean that while the overall trajectory is positive, some subpopulations and injury causes have not improved as quickly (35). Our projections should be interpreted as trend-continuation forecasts rather than scenario-based futures. They may understate accelerated gains that could follow scale-up of proven policies (e.g., universal child-restraint/helmet enforcement, speed management, clean cooking adoption, swimming instruction at population scale) or overstate progress if emerging countervailing forces intensify (rapid motorization without safety infrastructure, climate-related flood exposure, conflict). To enhance policy relevance, future work could incorporate exploratory scenarios that vary key levers, road-safety enforcement, emergency care capacity, water-safety programming, and climate or conflict shocks, and could triangulate BAPC estimates with alternative specifications (e.g., different priors, shorter calibration windows, ARIMA/baseline GAMs) as sensitivity analyses.

### Limitations

This study has several important limitations. First, our analyses rely on modeled GBD 2021 estimates that synthesize country-reported statistics with multiple data systems. In many LMICs, injury surveillance and death registration are incomplete or absent, which can bias levels and trends; for example, drowning and road injuries may be under-reported or coded as events of undetermined intent. Although GBD employs systematic bias-adjustments and uncertainty propagation, residual under-ascertainment and misclassification are likely. Consequently, between country differences, particularly where data are sparse, should be interpreted as indicative trends rather than precise measurements. Second, we focused on broad categories of unintentional injuries and did not disaggregate by specific injury mechanisms beyond the major groups (e.g., we report overall trends rather than separate analyses for drownings, burns, falls, etc.). While this provides a comprehensive overview, it may obscure important cause-specific patterns. Third, our projections of injury burden to 2035 are based on the assumption that recent trends will continue linearly. This does not account for potential accelerated improvements or unexpected setbacks.

## Conclusion

In conclusion, this comprehensive global assessment reveals significant progress in reducing unintentional injuries among children and adolescents since 1990, but also delineates the considerable work still required. Injury burden has fallen overall, yet remains a leading cause of death and disability for young people and is marked by deep inequities between and within regions.

## Data Availability

The datasets presented in this study can be found in online repositories. The names of the repository/repositories and accession number(s) can be found in the article/Supplementary material.

## Footnotes

^1^ https://vizhub.healthdata.org/gbd-results/

## Supplementary material

SUPPLEMENTARY FIGURE S1 Trends in incidence, mortality, and disability-adjusted life years (DALYs) of unintentional injury by age and sex. **(A)** Incidence cases and rate. **(B)** Death cases and rate. **(C)** DALY cases and rate.
