# Supplementary figures and images for: Global patterns and health impact of unintentional injuries among children and adolescents, 1990–2021
Source: Front Public Health. 2025 Sep 24;13:1626739. doi: 10.3389/fpubh.2025.1626739 (PMC12504300; doi:10.3389/fpubh.2025.1626739)

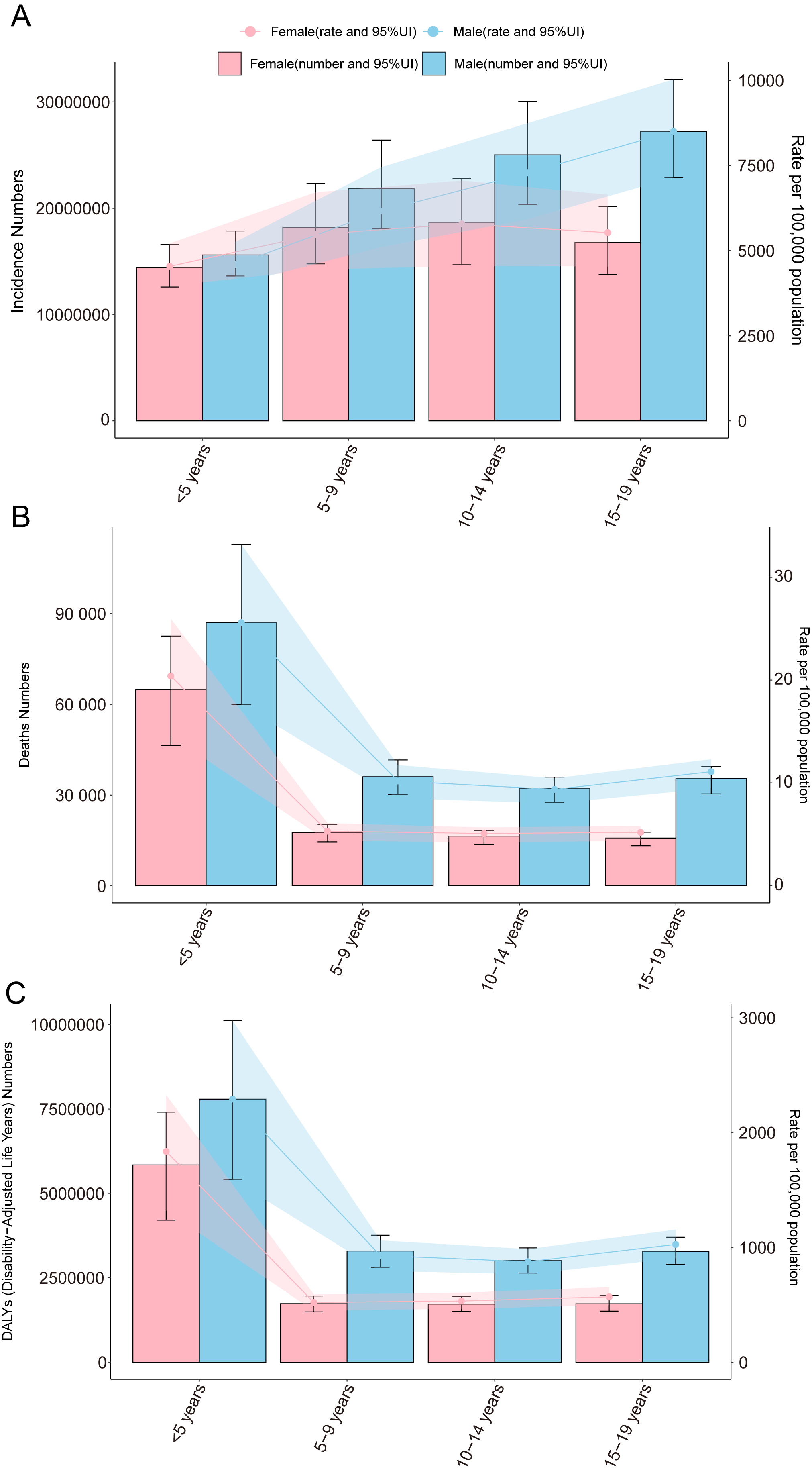

Supplement: SUPPLEMENTARY FIGURE S1 — Trends in incidence, mortality, and disability-adjusted life years (DALYs) of unintentional injury by age and sex. (A) Incidence cases and rate. (B) Death cases and rate. (C) DALY cases and rate. [file Image_1.TIF]
