# Supplementary material for: Global patterns and health impact of unintentional injuries among children and adolescents, 1990–2021
Source: Front Public Health. 2025 Sep 24;13:1626739. doi: 10.3389/fpubh.2025.1626739 (PMC12504300; doi:10.3389/fpubh.2025.1626739)
Supplement: Supplementary file 2 [file Table_1.DOCX]

Table S1: DALYs of unintentional injuries between 1990 and 2021 at the global and regional level.

|  | 1990 |  |  | 2021 |  |  | 1990-2021 |  |  |
| --- | --- | --- | --- | --- | --- | --- | --- | --- | --- |
| location | DALYs Cases | DALY Rate |  | DALYs Cases | DALY Rate |  | Cases change | Rate change | EAPC |
| Global | 70883768.80(63582671.03,78251185.62) | 3138.43(2815.17,3464.63) |  | 28410166.54(23100433.13,33513237.53) | 1077.84(876.40,1271.44) |  | -59.92(-67.11,-52.00) | -65.66(-71.82,-58.87) | -3.23(-3.48,-2.99) |
| High SDI | 2298540.64(2118140.60,2531226.12) | 914.59(842.81,1007.17) |  | 912258.53(799548.27,1073021.76) | 391.99(343.56,461.07) |  | -60.31(-63.04,-57.43) | -57.14(-60.09,-54.03) | -2.53(-2.70,-2.37) |
| High-middle SDI | 9044511.90(8190425.40,10043170.24) | 2443.37(2212.64,2713.15) |  | 1806204.03(1621729.08,2034709.74) | 595.39(534.58,670.72) |  | -80.03(-82.49,-77.42) | -75.63(-78.64,-72.45) | -4.65(-4.82,-4.49) |
| Middle SDI | 26932179.80(24602347.58,29686923.84) | 3522.60(3217.87,3882.91) |  | 6175564.61(5411332.89,7003093.15) | 824.30(722.29,934.75) |  | -77.07(-80.08,-73.97) | -76.60(-79.67,-73.43) | -4.29(-4.61,-3.97) |
| Low-middle SDI | 20180838.32(17340729.96,22928110.64) | 3414.56(2934.02,3879.39) |  | 8774061.55(7188394.95,10379481.05) | 1147.85(940.41,1357.88) |  | -56.52(-64.09,-46.69) | -66.38(-72.24,-58.78) | -3.49(-3.85,-3.13) |
| Low SDI | 12381317.37(10042804.05,14615292.86) | 4428.60(3592.15,5227.66) |  | 10712770.20(7729199.47,13508499.90) | 1833.73(1323.03,2312.28) |  | -13.48(-35.34,15.68) | -58.59(-69.05,-44.64) | -2.61(-3.04,-2.18) |
| Regions |  |  |  |  |  |  |  |  |  |
| Andean Latin America | 865253.33(705333.84,963784.70) | 4564.51(3720.88,5084.30) |  | 272963.87(225602.33,335987.49) | 1153.03(952.97,1419.25) |  | -68.45(-74.38,-60.42) | -74.74(-79.48,-68.31) | -4.43(-4.67,-4.19) |
| Australasia | 56586.71(47681.35,68492.21) | 902.05(760.09,1091.83) |  | 41556.20(32018.65,54653.50) | 551.01(424.55,724.68) |  | -26.56(-33.84,-19.81) | -38.92(-44.97,-33.29) | -1.27(-1.46,-1.09) |
| Caribbean | 493655.30(416772.00,569753.43) | 3269.46(2760.27,3773.46) |  | 421479.64(361080.77,492401.03) | 2761.53(2365.79,3226.20) |  | -14.62(-27.10,-0.19) | -15.54(-27.88,-1.26) | -0.34(-2.78,2.16) |
| Central Asia | 1029101.69(955671.35,1119556.59) | 3258.73(3026.20,3545.16) |  | 417487.31(363676.52,488366.73) | 1205.75(1050.34,1410.46) |  | -59.43(-65.11,-52.61) | -63.00(-68.18,-56.78) | -3.34(-3.45,-3.23) |
| Central Europe | 589071.73(535640.27,659738.15) | 1500.12(1364.05,1680.07) |  | 116881.80(96404.57,145648.41) | 496.16(409.24,618.28) |  | -80.16(-82.31,-77.74) | -66.93(-70.51,-62.90) | -3.58(-3.68,-3.48) |
| Central Latin America | 1891063.18(1751765.33,2053469.10) | 2288.56(2119.98,2485.10) |  | 687514.47(580253.95,813541.97) | 806.14(680.37,953.91) |  | -63.64(-68.37,-57.96) | -64.78(-69.35,-59.27) | -3.13(-3.68,-2.59) |
| Central Sub-Saharan Africa | 1622333.90(1299684.81,1969164.42) | 5235.33(4194.13,6354.56) |  | 1146454.29(789346.08,1563445.69) | 1558.51(1073.05,2125.38) |  | -29.33(-47.20,-1.72) | -70.23(-77.76,-58.60) | -3.51(-3.84,-3.19) |
| East Asia | 19875028.62(17400154.28,22808816.89) | 4319.34(3781.49,4956.93) |  | 2782077.96(2421627.28,3182245.11) | 806.51(702.02,922.52) |  | -86.00(-88.31,-83.44) | -81.33(-84.41,-77.91) | -5.26(-5.50,-5.02) |
| Eastern Europe | 1472008.29(1392289.47,1573885.91) | 2188.03(2069.54,2339.47) |  | 318641.50(289830.13,359135.94) | 690.30(627.89,778.03) |  | -78.35(-79.59,-77.04) | -68.45(-70.25,-66.53) | -4.13(-4.57,-3.69) |
| Eastern Sub-Saharan Africa | 4272772.22(3450175.45,5040135.06) | 3852.91(3111.14,4544.86) |  | 3191302.97(2360050.02,4352904.17) | 1402.25(1037.00,1912.65) |  | -25.31(-43.09,4.21) | -63.61(-72.27,-49.22) | -3.07(-3.16,-2.98) |
| High-income Asia Pacific | 455197.77(408796.68,510146.01) | 904.49(812.29,1013.67) |  | 94079.46(78353.98,116721.66) | 305.56(254.49,379.10) |  | -79.33(-81.95,-76.45) | -66.22(-70.49,-61.51) | -3.49(-4.02,-2.94) |
| High-income North America | 635248.28(595721.41,691174.97) | 777.25(728.89,845.68) |  | 375933.99(343681.15,418246.07) | 419.77(383.75,467.01) |  | -40.82(-45.12,-36.65) | -45.99(-49.91,-42.19) | -1.73(-2.00,-1.46) |
| North Africa and Middle East | 6939474.80(6129656.27,7784263.82) | 3925.66(3467.54,4403.56) |  | 2056205.74(1734252.05,2419240.80) | 869.46(733.32,1022.97) |  | -70.37(-74.96,-64.61) | -77.85(-81.28,-73.54) | -3.70(-4.02,-3.38) |
| Oceania | 65991.17(50675.38,82563.51) | 1960.30(1505.33,2452.58) |  | 112383.99(88342.76,145313.46) | 1759.74(1383.30,2275.37) |  | 70.30(35.49,113.70) | -10.23(-28.58,12.65) | -0.52(-1.22,0.17) |
| South Asia | 17981087.30(15050451.64,21013712.61) | 3314.92(2774.64,3874.01) |  | 7322999.41(5922433.11,8728555.14) | 1071.41(866.49,1277.05) |  | -59.27(-66.76,-48.41) | -67.68(-73.62,-59.06) | -3.69(-4.01,-3.38) |
| Southeast Asia | 5849651.16(5030256.29,6666367.52) | 2660.13(2287.51,3031.53) |  | 2321771.78(2020657.72,2659094.37) | 1012.67(881.34,1159.80) |  | -60.31(-66.13,-52.85) | -61.93(-67.51,-54.78) | -2.82(-3.79,-1.83) |
| Southern Latin America | 402614.70(382514.62,430456.99) | 2077.55(1973.83,2221.22) |  | 128354.42(111022.12,153871.07) | 657.90(569.06,788.69) |  | -68.12(-71.54,-63.74) | -68.33(-71.73,-63.98) | -3.48(-3.67,-3.29) |
| Southern Sub-Saharan Africa | 485380.96(417211.25,550904.19) | 1834.35(1576.72,2081.97) |  | 400177.78(334670.12,477108.11) | 1279.98(1070.45,1526.04) |  | -17.55(-30.87,-2.32) | -30.22(-41.49,-17.33) | -0.64(-0.93,-0.34) |
| Tropical Latin America | 1113896.78(1028302.86,1216651.39) | 1608.24(1484.66,1756.60) |  | 418553.38(366106.88,478317.86) | 628.58(549.81,718.33) |  | -62.42(-66.82,-57.30) | -60.92(-65.49,-55.59) | -2.70(-2.94,-2.46) |
| Western Europe | 673294.42(593926.12,788133.82) | 684.62(603.92,801.39) |  | 324953.82(259419.06,422588.23) | 354.32(282.87,460.78) |  | -51.74(-56.68,-46.34) | -48.25(-53.54,-42.46) | -1.89(-2.01,-1.77) |
| Western Sub-Saharan Africa | 4115056.49(3352020.36,4821328.80) | 3828.16(3118.33,4485.20) |  | 5458392.77(3347837.87,6960617.44) | 2032.34(1246.51,2591.67) |  | 32.64(-14.89,77.47) | -46.91(-65.94,-28.97) | -1.75(-1.96,-1.54) |
