# Supplementary material for: Global patterns and health impact of unintentional injuries among children and adolescents, 1990–2021
Source: Front Public Health. 2025 Sep 24;13:1626739. doi: 10.3389/fpubh.2025.1626739 (PMC12504300; doi:10.3389/fpubh.2025.1626739)
Supplement: Supplementary file 3 [file Table_2.DOCX]

Table S2. Incidence of Unintentional Injuries at the National Level

| location | 1990 | |  | 2021 | |  | 1990-2021 | | |
| --- | --- | --- | --- | --- | --- | --- | --- | --- | --- |
|  | Incident cases | Incidence rate |  | Incident cases | Incidence rate |  | Cases change | Rate Change | EAPC |
| Afghanistan | 389737.87(346959.36,433752.23) | 6993.37(6225.76,7783.16) |  | 928374.07(827494.55,1039147.41) | 5222.41(4654.93,5845.55) |  | 138.20(126.88,150.51) | -25.32(-28.88,-21.47) | -1.09(-1.31,-0.87) |
| Albania | 410167.20(346538.70,485169.18) | 28367.34(23966.76,33554.51) |  | 138010.71(116631.93,161092.98) | 22363.64(18899.36,26103.95) |  | -66.35(-68.52,-63.78) | -21.16(-26.24,-15.14) | -1.12(-1.35,-0.89) |
| Algeria | 970087.06(865729.81,1077110.28) | 7177.02(6404.95,7968.81) |  | 889771.31(773177.04,1019376.73) | 5409.33(4700.50,6197.26) |  | -8.28(-12.93,-3.17) | -24.63(-28.45,-20.43) | -1.01(-1.13,-0.89) |
| American Samoa | 911.31(788.10,1052.63) | 3808.78(3293.80,4399.40) |  | 660.56(566.92,758.04) | 3453.23(2963.72,3962.83) |  | -27.52(-31.05,-23.32) | -9.33(-13.75,-4.08) | -0.14(-1.30,1.02) |
| Andorra | 2256.73(1896.29,2692.40) | 16767.76(14089.67,20004.88) |  | 2274.00(1907.44,2737.68) | 15815.82(13266.37,19040.74) |  | 0.77(-3.29,5.07) | -5.68(-9.47,-1.65) | -0.17(-0.28,-0.07) |
| Angola | 318234.15(286260.70,357523.40) | 5519.13(4964.62,6200.52) |  | 721714.69(642333.65,815208.02) | 3864.45(3439.41,4365.07) |  | 126.79(116.79,136.66) | -29.98(-33.07,-26.93) | -1.24(-1.37,-1.11) |
| Antigua and Barbuda | 2440.96(2098.68,2803.48) | 10217.49(8784.75,11734.96) |  | 2652.35(2302.82,3106.13) | 11352.34(9856.31,13294.54) |  | 8.66(1.97,16.44) | 11.11(4.27,19.06) | 0.06(-0.36,0.49) |
| Argentina | 3148034.05(2667892.41,3673664.59) | 24165.80(20480.01,28200.79) |  | 2949034.67(2473558.56,3472589.51) | 21507.75(18040.03,25326.11) |  | -6.32(-10.37,-0.95) | -11.00(-14.84,-5.90) | -0.17(-0.55,0.21) |
| Armenia | 237248.14(208245.92,270788.92) | 17867.50(15683.30,20393.50) |  | 80649.04(68805.95,94029.77) | 10568.33(9016.40,12321.75) |  | -66.01(-68.64,-63.43) | -40.85(-45.43,-36.37) | -1.75(-1.83,-1.67) |
| Australia | 1894330.84(1552889.78,2251098.82) | 36626.19(30024.55,43524.16) |  | 2006580.12(1617035.67,2401816.87) | 32165.47(25921.07,38501.11) |  | 5.93(0.45,10.93) | -12.18(-16.72,-8.03) | -0.35(-0.43,-0.27) |
| Austria | 374950.04(322645.88,433387.85) | 20016.59(17224.35,23136.27) |  | 282107.28(237791.61,335115.60) | 16075.47(13550.20,19096.07) |  | -24.76(-29.72,-20.18) | -19.69(-24.98,-14.80) | -0.61(-0.69,-0.53) |
| Azerbaijan | 367977.06(316235.11,421776.55) | 11756.08(10103.03,13474.86) |  | 313381.98(265228.39,364014.04) | 10285.64(8705.17,11947.46) |  | -14.84(-18.37,-11.01) | -12.51(-16.14,-8.57) | -0.52(-0.59,-0.44) |
| Bahamas | 8213.81(6926.05,9649.10) | 7629.35(6433.23,8962.52) |  | 8696.05(7310.82,10247.52) | 7544.30(6342.54,8890.30) |  | 5.87(1.88,11.04) | -1.11(-4.84,3.71) | 0.40(-0.05,0.86) |
| Bahrain | 11781.98(10078.22,13662.68) | 5888.17(5036.70,6828.07) |  | 23619.70(20174.03,27626.70) | 5853.47(4999.56,6846.49) |  | 100.47(89.48,112.56) | -0.59(-6.04,5.41) | 0.07(-0.06,0.19) |
| Bangladesh | 2548695.50(2217595.68,2903219.44) | 4222.54(3673.99,4809.90) |  | 2581989.91(2235787.76,2943117.80) | 4233.53(3665.88,4825.65) |  | 1.31(-4.04,7.55) | 0.26(-5.03,6.44) | -0.80(-1.98,0.40) |
| Barbados | 6898.46(5809.13,8153.11) | 8124.99(6841.98,9602.72) |  | 5376.83(4519.42,6379.48) | 8100.01(6808.36,9610.48) |  | -22.06(-25.38,-18.64) | -0.31(-4.56,4.07) | -0.02(-0.19,0.14) |
| Belarus | 523737.29(446834.38,606305.17) | 16690.09(14239.40,19321.31) |  | 280572.08(236416.03,326038.03) | 13853.59(11673.33,16098.53) |  | -46.43(-48.81,-43.83) | -17.00(-20.69,-12.96) | -0.78(-0.98,-0.57) |
| Belgium | 454680.58(386378.24,534753.35) | 18395.35(15631.99,21634.91) |  | 429997.45(360435.91,521326.14) | 16900.76(14166.69,20490.37) |  | -5.43(-9.29,-0.65) | -8.12(-11.88,-3.48) | 0.09(-0.60,0.79) |
| Belize | 8988.89(7849.45,10315.09) | 8763.10(7652.29,10056.00) |  | 14476.72(12473.73,16776.13) | 8563.59(7378.74,9923.79) |  | 61.05(49.78,73.27) | -2.28(-9.11,5.14) | -0.11(-0.30,0.09) |
| Benin | 169823.67(151740.02,190443.09) | 5917.82(5287.66,6636.34) |  | 372390.20(336229.16,415233.99) | 4954.43(4473.33,5524.45) |  | 119.28(110.27,129.87) | -16.28(-19.72,-12.24) | -0.56(-0.63,-0.50) |
| Bermuda | 1337.77(1125.56,1588.44) | 8413.03(7078.48,9989.42) |  | 1030.63(859.15,1212.20) | 8964.19(7472.73,10543.46) |  | -22.96(-26.21,-19.33) | 6.55(2.05,11.58) | 0.26(0.11,0.40) |
| Bhutan | 19426.08(17074.63,21889.31) | 5703.25(5012.90,6426.43) |  | 15522.73(13926.62,17261.22) | 6087.93(5461.94,6769.76) |  | -20.09(-24.82,-15.32) | 6.74(0.42,13.12) | -0.20(-0.75,0.36) |
| Bolivia (Plurinational State of) | 290254.18(260991.89,322619.82) | 8667.26(7793.46,9633.72) |  | 327607.45(289229.08,375455.03) | 7205.25(6361.17,8257.59) |  | 12.87(7.68,18.68) | -16.87(-20.69,-12.59) | -0.68(-0.74,-0.63) |
| Bosnia and Herzegovina | 415266.78(351132.42,491343.87) | 28025.33(23697.06,33159.58) |  | 133079.07(107575.27,159953.66) | 20009.53(16174.83,24050.35) |  | -67.95(-70.74,-65.30) | -28.60(-34.80,-22.68) | -1.19(-1.38,-0.99) |
| Botswana | 36299.80(32305.13,40901.72) | 4902.25(4362.77,5523.73) |  | 42170.46(37814.40,47523.02) | 4598.71(4123.68,5182.41) |  | 16.17(11.51,20.77) | -6.19(-9.95,-2.48) | -0.29(-0.36,-0.23) |
| Brazil | 7440218.05(6371163.53,8650725.71) | 11072.97(9481.94,12874.52) |  | 4843748.36(4171078.86,5637861.64) | 7578.20(6525.79,8820.62) |  | -34.90(-37.42,-32.52) | -31.56(-34.22,-29.06) | -1.52(-1.83,-1.20) |
| Brunei Darussalam | 15682.60(13329.00,18234.71) | 13634.00(11587.85,15852.73) |  | 16194.86(13707.24,18846.38) | 12540.77(10614.44,14594.02) |  | 3.27(-0.47,7.42) | -8.02(-11.35,-4.32) | -0.30(-0.41,-0.18) |
| Bulgaria | 621665.89(532204.72,720303.00) | 26305.14(22519.69,30478.87) |  | 273149.56(227556.58,321516.26) | 21104.24(17581.61,24841.17) |  | -56.06(-58.31,-53.93) | -19.77(-23.88,-15.88) | -0.80(-0.87,-0.73) |
| Burkina Faso | 305773.21(270809.83,341524.09) | 5405.01(4786.98,6036.96) |  | 616223.10(552626.44,689564.27) | 4812.06(4315.43,5384.78) |  | 101.53(92.77,110.96) | -10.97(-14.84,-6.80) | -0.37(-0.40,-0.33) |
| Burundi | 185884.83(166956.22,208015.75) | 5891.51(5291.58,6592.94) |  | 340118.72(305024.29,381961.20) | 4675.47(4193.04,5250.66) |  | 82.97(75.14,91.00) | -20.64(-24.04,-17.16) | -0.74(-0.82,-0.66) |
| Cabo Verde | 11625.41(10388.97,13166.72) | 5977.95(5342.16,6770.52) |  | 9178.18(8070.43,10479.74) | 4761.81(4187.09,5437.08) |  | -21.05(-24.30,-17.72) | -20.34(-23.62,-16.98) | -0.72(-0.77,-0.68) |
| Cambodia | 295443.94(262705.25,330164.59) | 5189.22(4614.19,5799.06) |  | 293410.39(262023.68,330982.01) | 4424.76(3951.43,4991.35) |  | -0.69(-5.34,4.28) | -14.73(-18.72,-10.47) | -0.44(-0.66,-0.22) |
| Cameroon | 294200.39(262652.89,331313.01) | 4955.32(4423.95,5580.42) |  | 756514.79(676462.45,846299.34) | 4475.68(4002.08,5006.86) |  | 157.14(146.02,168.66) | -9.68(-13.59,-5.63) | -0.29(-0.38,-0.20) |
| Canada | 754762.03(643922.07,881969.85) | 9847.25(8401.14,11506.90) |  | 659361.90(560897.65,775837.10) | 8012.79(6816.22,9428.23) |  | -12.64(-15.99,-9.23) | -18.63(-21.75,-15.45) | -0.65(-0.68,-0.62) |
| Central African Republic | 74737.17(67307.73,83133.20) | 4991.99(4495.75,5552.79) |  | 123799.27(111508.63,137551.21) | 4277.78(3853.09,4752.97) |  | 65.65(59.39,72.18) | -14.31(-17.54,-10.93) | -0.48(-0.54,-0.42) |
| Chad | 197733.95(176816.77,220909.34) | 5609.02(5015.68,6266.43) |  | 566065.78(509402.56,626543.32) | 5169.76(4652.26,5722.08) |  | 186.28(173.60,200.27) | -7.83(-11.91,-3.33) | -0.26(-0.31,-0.20) |
| Chile | 1011509.38(842839.84,1190910.83) | 19177.18(15979.38,22578.44) |  | 1158657.56(986727.20,1343236.80) | 23676.33(20163.05,27448.07) |  | 14.55(6.04,23.41) | 23.46(14.29,33.01) | 0.91(0.80,1.02) |
| China | 19514869.42(16717335.23,22461720.62) | 4384.95(3756.35,5047.11) |  | 12762269.99(10798355.15,14814970.51) | 3817.57(3230.10,4431.59) |  | -34.60(-37.69,-31.76) | -12.94(-17.05,-9.16) | -1.10(-1.59,-0.62) |
| Colombia | 2376650.20(2059162.77,2731946.53) | 15788.73(13679.58,18149.06) |  | 1621069.09(1362232.22,1891921.29) | 11134.17(9356.37,12994.49) |  | -31.79(-35.41,-27.61) | -29.48(-33.22,-25.15) | -1.06(-1.12,-1.00) |
| Comoros | 14158.84(12562.94,16137.97) | 5356.75(4752.97,6105.52) |  | 14275.12(12642.55,16353.10) | 4546.97(4026.96,5208.86) |  | 0.82(-2.66,4.82) | -15.12(-18.04,-11.75) | -0.56(-0.66,-0.47) |
| Congo | 60401.55(53854.44,68094.18) | 4552.82(4059.33,5132.66) |  | 92236.26(81401.84,105422.38) | 3708.97(3273.30,4239.21) |  | 52.71(46.67,58.57) | -18.53(-21.75,-15.41) | -0.72(-0.80,-0.64) |
| Cook Islands | 381.46(334.02,440.89) | 4430.22(3879.18,5120.43) |  | 183.04(152.18,219.75) | 3531.76(2936.16,4239.98) |  | -52.01(-55.52,-47.60) | -20.28(-26.10,-12.94) | -0.52(-1.58,0.57) |
| Costa Rica | 194405.54(161214.73,229578.95) | 13708.38(11367.96,16188.62) |  | 163146.11(133997.03,194359.44) | 11917.39(9788.12,14197.44) |  | -16.08(-18.95,-12.80) | -13.06(-16.04,-9.67) | -0.51(-0.59,-0.42) |
| Croatia | 371800.17(330631.68,418580.59) | 5374.00(4778.95,6050.16) |  | 688104.98(616395.48,767230.51) | 4783.61(4285.09,5333.68) |  | -54.05(-57.40,-50.63) | -24.35(-29.86,-18.72) | -0.36(-0.41,-0.30) |
| Cuba | 305031.83(254541.98,356717.43) | 22996.32(19189.89,26892.89) |  | 140171.82(117691.43,162776.18) | 17396.87(14606.80,20202.32) |  | -25.35(-29.33,-21.09) | 13.54(7.49,20.02) | -0.72(-0.82,-0.62) |
| Cyprus | 351544.26(305273.09,400124.15) | 9626.02(8359.02,10956.24) |  | 262431.16(221393.01,307353.12) | 10929.19(9220.12,12800.01) |  | 5.32(0.44,10.57) | -4.29(-8.73,0.47) | 0.52(0.28,0.76) |
| Czechia | 41003.52(34479.83,48301.11) | 15827.71(13309.51,18644.64) |  | 43186.44(36150.44,51479.26) | 15148.49(12680.47,18057.36) |  | -47.70(-50.33,-44.67) | -28.31(-31.92,-24.16) | -0.04(-0.23,0.14) |
| C么te d'Ivoire | 879267.29(717894.61,1049587.59) | 28886.32(23584.79,34481.80) |  | 459865.32(368391.96,555200.90) | 20709.82(16590.36,25003.21) |  | 85.07(77.83,92.59) | -10.99(-14.47,-7.37) | -0.58(-0.94,-0.22) |
| Democratic People's Republic of Korea | 199575.07(178187.82,223817.65) | 2562.15(2287.58,2873.38) |  | 120353.11(107160.71,135735.51) | 1822.90(1623.09,2055.89) |  | -39.70(-43.89,-35.84) | -28.85(-33.80,-24.30) | -0.89(-1.10,-0.69) |
| Democratic Republic of the Congo | 1081627.35(967906.29,1227925.36) | 4995.93(4470.66,5671.66) |  | 1909274.51(1689149.27,2162368.37) | 3985.38(3525.89,4513.68) |  | 76.52(68.32,84.79) | -20.23(-23.93,-16.49) | -0.74(-0.86,-0.62) |
| Denmark | 198115.00(167235.65,235969.49) | 15845.69(13375.89,18873.38) |  | 201445.31(166035.48,240493.63) | 15565.16(12829.14,18582.33) |  | 1.68(-3.09,7.34) | -1.77(-6.38,3.70) | 0.06(-0.08,0.21) |
| Djibouti | 11733.01(10446.01,13375.16) | 5231.57(4657.71,5963.78) |  | 24895.29(22294.52,28227.13) | 4711.70(4219.48,5342.29) |  | 112.18(103.40,120.53) | -9.94(-13.66,-6.39) | -0.54(-0.84,-0.24) |
| Dominica | 2454.17(2100.89,2844.30) | 7488.47(6410.51,8678.90) |  | 1487.04(1282.81,1722.54) | 7654.94(6603.64,8867.29) |  | -39.41(-42.42,-36.10) | 2.22(-2.85,7.80) | 0.51(0.04,0.99) |
| Dominican Republic | 260106.28(225386.67,298051.31) | 7427.06(6435.68,8510.54) |  | 298689.47(256142.81,347153.71) | 7677.04(6583.49,8922.69) |  | 14.83(9.72,20.10) | 3.37(-1.23,8.10) | 0.11(-0.11,0.34) |
| Ecuador | 432351.78(383614.15,488575.37) | 8721.12(7738.02,9855.23) |  | 522228.43(455942.71,595302.56) | 7893.10(6891.24,8997.56) |  | 20.79(15.47,25.93) | -9.49(-13.48,-5.64) | -0.39(-0.52,-0.25) |
| Egypt | 1900830.53(1688242.52,2141471.95) | 6807.46(6046.12,7669.27) |  | 2271707.81(1967865.89,2588342.07) | 4880.12(4227.40,5560.31) |  | 19.51(12.66,26.89) | -28.31(-32.42,-23.89) | -1.20(-1.30,-1.10) |
| El Salvador | 367210.70(315861.50,428224.33) | 13326.97(11463.38,15541.30) |  | 251635.31(210124.30,296576.75) | 10563.69(8821.05,12450.34) |  | -31.47(-34.91,-27.88) | -20.73(-24.71,-16.57) | -0.86(-1.03,-0.69) |
| Equatorial Guinea | 12877.41(11654.82,14487.62) | 5366.03(4856.58,6037.00) |  | 28163.39(24751.71,32281.44) | 3656.97(3213.97,4191.69) |  | 118.70(107.01,133.53) | -31.85(-35.50,-27.23) | -1.35(-1.40,-1.31) |
| Eritrea | 113418.50(101294.63,128076.07) | 5766.68(5150.25,6511.94) |  | 155264.04(139583.92,174103.33) | 4826.89(4339.42,5412.57) |  | 36.89(31.51,42.79) | -16.30(-19.59,-12.69) | -0.59(-0.66,-0.52) |
| Estonia | 89151.84(77051.66,102412.63) | 19409.42(16775.06,22296.45) |  | 37304.54(31301.98,44302.96) | 13308.69(11167.23,15805.43) |  | -58.16(-61.16,-55.26) | -31.43(-36.35,-26.68) | -1.52(-1.64,-1.41) |
| Eswatini | 22219.14(19700.94,25212.76) | 4656.86(4129.08,5284.29) |  | 23125.43(20819.21,26047.62) | 4322.46(3891.39,4868.66) |  | 4.08(-0.47,8.92) | -7.18(-11.24,-2.86) | -0.20(-0.30,-0.11) |
| Ethiopia | 2010389.62(1800404.86,2267075.77) | 6822.46(6109.85,7693.55) |  | 2596428.34(2326390.58,2966997.04) | 4539.37(4067.26,5187.24) |  | 29.15(25.03,33.58) | -33.46(-35.59,-31.18) | -1.36(-1.46,-1.27) |
| Fiji | 12012.28(10409.29,13795.36) | 3348.78(2901.90,3845.87) |  | 10900.74(9576.76,12504.08) | 3108.91(2731.31,3566.18) |  | -9.25(-13.63,-5.11) | -7.16(-11.64,-2.92) | -0.42(-0.72,-0.12) |
| Finland | 279666.54(234672.14,334192.43) | 22042.31(18496.02,26339.85) |  | 235836.24(195953.50,288197.16) | 20533.97(17061.43,25092.97) |  | -15.67(-19.35,-12.32) | -6.84(-10.90,-3.14) | -0.31(-0.93,0.31) |
| France | 2875933.27(2463332.43,3327570.76) | 17816.56(15260.48,20614.48) |  | 2545546.33(2143758.23,3030344.90) | 16160.51(13609.74,19238.27) |  | -11.49(-15.44,-7.24) | -9.30(-13.34,-4.94) | -0.16(-0.28,-0.05) |
| Gabon | 25114.70(22209.85,28412.47) | 4942.08(4370.46,5591.01) |  | 32995.71(29320.07,37647.34) | 3989.57(3545.14,4552.01) |  | 31.38(26.45,36.77) | -19.27(-22.30,-15.96) | -0.63(-0.69,-0.57) |
| Gambia | 31174.60(27674.91,35376.28) | 5549.14(4926.19,6297.05) |  | 58278.72(51795.58,65374.57) | 4601.13(4089.28,5161.35) |  | 86.94(79.78,95.23) | -17.08(-20.26,-13.41) | -0.72(-0.85,-0.58) |
| Georgia | 272693.92(234425.72,310971.35) | 15176.49(13046.71,17306.78) |  | 128791.29(114170.90,144523.95) | 13859.01(12285.74,15551.98) |  | -52.77(-55.95,-49.54) | -8.68(-14.82,-2.43) | -0.24(-0.30,-0.17) |
| Germany | 2726061.62(2323260.00,3208201.15) | 15729.92(13405.67,18511.96) |  | 2415632.17(2024261.90,2879419.76) | 15147.92(12693.72,18056.23) |  | -11.39(-15.29,-7.50) | -3.70(-7.94,0.53) | -0.09(-0.20,0.03) |
| Ghana | 403198.30(357767.04,457076.02) | 4881.57(4331.53,5533.88) |  | 723770.10(640094.94,821140.01) | 4436.49(3923.59,5033.34) |  | 79.51(72.59,86.89) | -9.12(-12.62,-5.38) | -0.29(-0.38,-0.21) |
| Greece | 474543.55(404663.85,563170.55) | 16884.32(14397.99,20037.68) |  | 276040.17(230532.01,328239.13) | 14488.02(12099.52,17227.70) |  | -41.83(-44.29,-39.30) | -14.19(-17.82,-10.46) | -0.47(-0.59,-0.36) |
| Greenland | 1990.14(1754.72,2266.68) | 11123.16(9807.35,12668.81) |  | 1241.49(1092.21,1424.02) | 8077.02(7105.88,9264.60) |  | -37.62(-41.28,-34.11) | -27.39(-31.65,-23.30) | -1.26(-1.39,-1.14) |
| Grenada | 3670.74(3179.49,4175.48) | 8706.88(7541.64,9904.08) |  | 2827.40(2442.93,3261.44) | 9391.92(8114.79,10833.68) |  | -22.97(-27.62,-17.55) | 7.87(1.36,15.46) | 0.36(0.13,0.59) |
| Guam | 1973.66(1694.30,2306.06) | 3645.16(3129.20,4259.06) |  | 1516.37(1296.60,1785.34) | 3127.49(2674.22,3682.23) |  | -23.17(-26.80,-19.05) | -14.20(-18.26,-9.60) | -0.52(-0.68,-0.36) |
| Guatemala | 753278.99(655943.94,868697.84) | 15339.33(13357.26,17689.65) |  | 861221.57(749247.68,1001342.16) | 13019.34(11326.60,15137.59) |  | 14.33(8.22,20.58) | -15.12(-19.66,-10.48) | -0.54(-0.58,-0.49) |
| Guinea | 204563.30(182894.11,229779.79) | 6223.06(5563.86,6990.17) |  | 379285.16(340886.83,419900.09) | 5081.22(4566.80,5625.33) |  | 85.41(75.97,93.27) | -18.35(-22.51,-14.89) | -0.62(-0.67,-0.58) |
| Guinea-Bissau | 36087.02(32326.70,40280.20) | 6136.25(5496.84,6849.26) |  | 50174.29(45164.88,55783.42) | 4485.76(4037.90,4987.23) |  | 39.04(33.55,45.01) | -26.90(-29.78,-23.76) | -1.08(-1.16,-1.00) |
| Guyana | 31788.51(28172.84,36122.12) | 8330.61(7383.07,9466.29) |  | 24541.93(21873.32,27662.12) | 8742.72(7792.06,9854.24) |  | -22.80(-26.23,-19.54) | 4.95(0.28,9.37) | 0.15(-0.02,0.31) |
| Haiti | 325988.34(291205.63,365555.45) | 9741.39(8701.99,10923.76) |  | 639808.13(575916.31,718152.05) | 11409.36(10270.01,12806.42) |  | 96.27(83.53,109.67) | 17.12(9.52,25.12) | 0.20(-1.30,1.72) |
| Honduras | 390491.92(337041.45,452098.89) | 14390.20(12420.47,16660.51) |  | 476909.52(406506.25,559101.29) | 10962.20(9343.92,12851.46) |  | 22.13(16.18,28.47) | -23.82(-27.53,-19.87) | -1.20(-1.82,-0.59) |
| Hungary | 623371.72(499274.39,753990.03) | 21479.14(17203.19,25979.77) |  | 357484.58(287143.29,428810.85) | 19061.99(15311.21,22865.29) |  | -42.65(-45.29,-40.11) | -11.25(-15.33,-7.32) | -0.32(-0.44,-0.21) |
| Iceland | 14652.65(12498.31,17300.15) | 17337.91(14788.77,20470.60) |  | 14022.77(11724.05,16905.16) | 15737.39(13157.61,18972.23) |  | -4.30(-9.10,0.35) | -9.23(-13.79,-4.82) | -0.31(-0.46,-0.16) |
| India | 29488901.96(25843240.55,33513219.05) | 7185.12(6296.84,8165.67) |  | 24115574.05(21137413.69,27296090.99) | 4817.60(4222.65,5452.98) |  | -18.22(-22.19,-14.31) | -32.95(-36.20,-29.75) | -1.49(-1.60,-1.37) |
| Indonesia | 4828404.22(4219280.13,5516595.87) | 5520.49(4824.05,6307.32) |  | 3651812.62(3207190.02,4171146.61) | 4047.77(3554.94,4623.42) |  | -24.37(-26.54,-22.31) | -26.68(-28.79,-24.68) | -0.86(-1.11,-0.60) |
| Iran (Islamic Republic of) | 3121666.74(2789722.24,3472662.53) | 9907.05(8853.58,11020.99) |  | 1434535.46(1253083.16,1648801.42) | 5548.48(4846.66,6377.21) |  | -54.05(-56.80,-51.32) | -43.99(-47.36,-40.67) | -1.45(-1.74,-1.17) |
| Iraq | 1178369.73(1040783.57,1324688.50) | 11442.11(10106.13,12862.88) |  | 1365578.19(1194919.08,1561350.09) | 7742.72(6775.10,8852.73) |  | 15.89(7.45,24.52) | -32.33(-37.26,-27.29) | -1.42(-1.68,-1.16) |
| Ireland | 238123.76(202910.25,277272.67) | 17919.99(15270.00,20866.14) |  | 218146.82(182025.30,261864.85) | 16506.32(13773.14,19814.29) |  | -8.39(-13.68,-3.06) | -7.89(-13.20,-2.53) | -0.19(-0.31,-0.08) |
| Israel | 355633.03(304505.93,414960.14) | 17725.72(15177.40,20682.74) |  | 503300.90(423811.13,595247.91) | 14919.59(12563.24,17645.22) |  | 41.52(32.90,48.71) | -15.83(-20.96,-11.55) | -0.41(-0.52,-0.31) |
| Italy | 2916769.55(2491828.52,3458823.12) | 21415.83(18295.78,25395.76) |  | 1783724.87(1520468.13,2102543.39) | 17020.92(14508.83,20063.20) |  | -38.85(-43.49,-33.50) | -20.52(-26.56,-13.57) | -0.88(-1.06,-0.70) |
| Jamaica | 108873.81(92505.15,126992.83) | 9965.47(8467.21,11623.95) |  | 69877.70(59196.54,81742.34) | 8570.09(7260.11,10025.22) |  | -35.82(-38.63,-32.64) | -14.00(-17.77,-9.75) | -0.49(-0.61,-0.38) |
| Japan | 5158959.21(4389000.76,6078196.83) | 15462.83(13155.05,18218.04) |  | 2616284.19(2198442.58,3096747.69) | 12321.66(10353.79,14584.45) |  | -49.29(-50.27,-48.26) | -20.31(-21.86,-18.70) | -0.76(-0.89,-0.62) |
| Jordan | 143444.58(126215.99,161750.09) | 6841.66(6019.94,7714.75) |  | 283086.71(245395.65,324743.31) | 5747.85(4982.56,6593.66) |  | 97.35(87.64,108.09) | -15.99(-20.12,-11.42) | -0.58(-0.66,-0.49) |
| Kazakhstan | 1016030.91(887841.92,1162238.01) | 15294.52(13364.86,17495.40) |  | 753435.34(651170.51,861127.28) | 11205.04(9684.17,12806.63) |  | -25.85(-29.85,-21.52) | -26.74(-30.70,-22.47) | -1.18(-1.28,-1.08) |
| Kenya | 723556.80(632854.84,831372.56) | 5246.27(4588.62,6028.01) |  | 1020128.30(906221.30,1171148.17) | 4146.79(3683.76,4760.68) |  | 40.99(36.93,45.58) | -20.96(-23.23,-18.38) | -0.96(-1.15,-0.77) |
| Kiribati | 897.24(775.56,1045.02) | 2447.93(2115.95,2851.10) |  | 1215.91(1051.84,1407.05) | 2275.06(1968.07,2632.70) |  | 35.52(29.30,41.00) | -7.06(-11.33,-3.30) | -0.21(-0.41,-0.01) |
| Kuwait | 44158.33(37787.44,50773.33) | 6437.49(5508.73,7401.84) |  | 67843.60(57164.51,78989.92) | 6193.14(5218.29,7210.64) |  | 53.64(47.10,60.30) | -3.80(-7.89,0.37) | -0.13(-0.25,-0.02) |
| Kyrgyzstan | 271897.65(238488.28,305549.87) | 12877.68(11295.34,14471.52) |  | 253212.24(213243.67,295346.06) | 8970.16(7554.26,10462.77) |  | -6.87(-12.24,-1.66) | -30.34(-34.36,-26.44) | -1.38(-1.47,-1.29) |
| Lao People's Democratic Republic | 108564.86(97224.71,122034.80) | 4785.80(4285.90,5379.58) |  | 105126.84(92830.53,118590.23) | 3514.16(3103.12,3964.21) |  | -3.17(-7.39,1.12) | -26.57(-29.77,-23.32) | -0.93(-1.03,-0.84) |
| Latvia | 142407.61(123915.46,163498.62) | 18926.68(16468.98,21729.78) |  | 49820.59(42017.51,59298.76) | 12870.75(10854.89,15319.36) |  | -65.02(-67.34,-62.63) | -32.00(-36.52,-27.36) | -1.53(-1.66,-1.40) |
| Lebanon | 80191.22(68456.02,92556.79) | 5988.29(5111.96,6911.69) |  | 97684.50(83384.86,113324.41) | 5862.58(5004.38,6801.22) |  | 21.81(16.10,28.34) | -2.10(-6.69,3.15) | -0.16(-0.31,-0.00) |
| Lesotho | 38827.15(34587.32,44173.13) | 4637.07(4130.71,5275.53) |  | 35793.04(32244.22,40035.42) | 4267.05(3843.98,4772.81) |  | -7.81(-12.00,-3.04) | -7.98(-12.16,-3.22) | -0.15(-0.21,-0.08) |
| Liberia | 79247.66(70558.80,89578.95) | 5800.74(5164.73,6556.96) |  | 117578.47(104207.96,133922.87) | 4214.23(3735.00,4800.04) |  | 48.37(40.72,55.69) | -27.35(-31.09,-23.77) | -1.07(-1.11,-1.03) |
| Libya | 147682.97(128340.54,167294.83) | 6397.94(5559.99,7247.57) |  | 114637.42(99947.26,131923.75) | 5491.54(4787.83,6319.62) |  | -22.38(-26.50,-18.07) | -14.17(-18.73,-9.40) | -0.46(-0.55,-0.37) |
| Lithuania | 184375.45(156920.69,214538.94) | 16645.84(14167.16,19369.07) |  | 68207.96(56465.70,80488.09) | 12730.30(10538.73,15022.26) |  | -63.01(-64.81,-61.16) | -23.52(-27.25,-19.71) | -0.96(-1.12,-0.80) |
| Luxembourg | 17060.25(14881.33,19623.61) | 19335.56(16866.05,22240.80) |  | 21606.49(18377.91,25475.02) | 16014.41(13621.44,18881.72) |  | 26.65(19.19,34.37) | -17.18(-22.05,-12.13) | -0.58(-0.66,-0.50) |
| Madagascar | 401219.73(359025.36,452392.92) | 5965.22(5337.89,6726.05) |  | 654256.15(581345.55,739976.38) | 4378.12(3890.22,4951.74) |  | 63.07(56.48,69.45) | -26.61(-29.57,-23.73) | -0.94(-1.03,-0.85) |
| Malawi | 321037.28(288746.12,362067.47) | 5760.28(5180.89,6496.48) |  | 467835.52(416012.68,533444.24) | 4417.35(3928.04,5036.84) |  | 45.73(39.67,52.84) | -23.31(-26.50,-19.57) | -0.89(-0.96,-0.81) |
| Malaysia | 342109.06(292925.77,399867.56) | 4100.56(3511.05,4792.87) |  | 382170.60(330418.94,442403.61) | 3726.60(3221.96,4313.94) |  | 11.71(6.79,16.92) | -9.12(-13.12,-4.88) | -0.38(-0.47,-0.29) |
| Maldives | 7261.58(6383.95,8254.17) | 5657.92(4974.11,6431.31) |  | 5631.78(4849.21,6526.67) | 4321.96(3721.40,5008.72) |  | -22.44(-27.63,-17.52) | -23.61(-28.72,-18.76) | -0.84(-1.56,-0.11) |
| Mali | 299978.93(268121.65,333552.88) | 6065.25(5421.13,6744.08) |  | 759557.41(688548.88,843816.64) | 5336.56(4837.67,5928.56) |  | 153.20(143.07,165.04) | -12.01(-15.53,-7.90) | -0.36(-0.40,-0.32) |
| Malta | 20989.11(17806.69,24787.12) | 18211.76(15450.44,21507.20) |  | 14212.02(12082.97,16828.16) | 16927.88(14391.99,20043.97) |  | -32.29(-35.19,-29.22) | -7.05(-11.03,-2.83) | -0.11(-0.33,0.11) |
| Marshall Islands | 857.71(738.07,998.66) | 3202.55(2755.85,3728.84) |  | 667.17(582.33,762.07) | 2891.67(2523.98,3303.03) |  | -22.22(-25.81,-18.01) | -9.71(-13.88,-4.83) | -0.41(-0.52,-0.29) |
| Mauritania | 57828.75(51509.10,65258.33) | 5099.89(4542.57,5755.10) |  | 99168.53(87706.18,112858.56) | 4252.20(3760.71,4839.20) |  | 71.49(64.78,78.39) | -16.62(-19.88,-13.27) | -0.62(-0.71,-0.53) |
| Mauritius | 19281.08(16735.57,22244.71) | 4475.50(3884.64,5163.41) |  | 11202.38(9606.59,13081.15) | 3773.06(3235.58,4405.85) |  | -41.90(-44.29,-39.42) | -15.70(-19.17,-12.10) | -0.41(-0.49,-0.33) |
| Mexico | 7431635.51(6262976.65,8618164.13) | 17136.55(14441.75,19872.56) |  | 4630173.70(3869171.61,5432900.63) | 10730.21(8966.62,12590.49) |  | -37.70(-40.02,-35.36) | -37.38(-39.72,-35.04) | -0.01(-0.77,0.76) |
| Micronesia (Federated States of) | 1863.09(1622.58,2145.26) | 3240.29(2821.99,3731.03) |  | 1232.82(1071.64,1441.36) | 2974.64(2585.72,3477.82) |  | -33.83(-36.86,-30.46) | -8.20(-12.40,-3.52) | -0.34(-1.17,0.50) |
| Monaco | 773.98(651.32,921.29) | 15975.80(13443.95,19016.57) |  | 1022.78(846.99,1232.00) | 15107.75(12511.20,18198.27) |  | 32.15(25.72,37.88) | -5.43(-10.03,-1.33) | -0.14(-0.25,-0.02) |
| Mongolia | 141637.14(124834.31,160921.55) | 12477.69(10997.43,14176.58) |  | 154239.81(136637.95,176313.27) | 11749.82(10408.93,13431.36) |  | 8.90(3.55,14.47) | -5.83(-10.46,-1.01) | -0.03(-0.18,0.12) |
| Montenegro | 47763.72(39133.38,56091.04) | 22265.58(18242.45,26147.44) |  | 29017.16(23411.56,34592.84) | 19294.64(15567.25,23002.12) |  | -39.25(-41.55,-36.49) | -13.34(-16.63,-9.40) | -0.48(-0.60,-0.36) |
| Morocco | 988591.10(882907.79,1108415.30) | 7921.50(7074.67,8881.65) |  | 806636.36(710757.46,905741.64) | 6245.43(5503.08,7012.76) |  | -18.41(-21.97,-14.56) | -21.16(-24.60,-17.44) | -0.92(-1.05,-0.80) |
| Mozambique | 430684.19(383413.74,483888.23) | 5688.83(5064.44,6391.59) |  | 809267.04(719277.69,913998.41) | 4558.19(4051.32,5148.08) |  | 87.90(81.08,94.53) | -19.87(-22.79,-17.05) | -0.79(-0.91,-0.66) |
| Myanmar | 1266666.89(1140613.57,1408456.86) | 6627.86(5968.28,7369.78) |  | 1060973.60(953806.02,1177560.99) | 5108.66(4592.64,5670.03) |  | -16.24(-20.45,-12.02) | -22.92(-26.80,-19.04) | -0.80(-2.12,0.53) |
| Namibia | 33827.80(29943.51,38354.66) | 4441.21(3931.25,5035.54) |  | 43720.13(38970.13,49884.16) | 4080.71(3637.36,4656.04) |  | 29.24(24.47,34.56) | -8.12(-11.51,-4.34) | -0.23(-0.38,-0.07) |
| Nauru | 169.14(145.81,193.89) | 3246.17(2798.46,3721.17) |  | 163.38(142.29,186.00) | 3185.29(2774.09,3626.28) |  | -3.40(-7.65,2.01) | -1.88(-6.19,3.63) | -0.16(-0.29,-0.02) |
| Nepal | 657101.03(586255.67,739468.20) | 6349.53(5664.95,7145.44) |  | 730883.95(649020.89,825723.23) | 5868.42(5211.13,6629.91) |  | 11.23(5.58,16.97) | -7.58(-12.27,-2.81) | -0.11(-0.78,0.56) |
| Netherlands | 497601.94(421678.45,589214.81) | 12981.81(11001.06,15371.88) |  | 455790.87(383182.41,543274.53) | 12348.49(10381.35,14718.64) |  | -8.40(-11.78,-5.13) | -4.88(-8.38,-1.49) | -0.10(-0.35,0.16) |
| New Zealand | 442663.78(366243.35,530388.91) | 40202.38(33261.93,48169.51) |  | 495250.93(423034.87,565610.99) | 37994.60(32454.34,43392.47) |  | 11.88(3.13,20.93) | -5.49(-12.88,2.15) | -0.29(-0.54,-0.04) |
| Nicaragua | 302469.64(256394.60,352976.70) | 13441.51(11393.97,15686.00) |  | 289482.23(240615.78,337676.38) | 11137.10(9257.09,12991.25) |  | -4.29(-8.94,0.06) | -17.14(-21.17,-13.38) | -0.75(-1.09,-0.40) |
| Niger | 293985.70(262387.11,329797.48) | 6059.57(5408.27,6797.71) |  | 787563.42(708791.72,875146.77) | 5052.77(4547.39,5614.68) |  | 167.89(156.07,180.24) | -16.62(-20.29,-12.77) | -0.62(-0.69,-0.55) |
| Nigeria | 2746791.24(2438254.92,3112443.11) | 5661.27(5025.36,6414.90) |  | 5509615.48(4901095.16,6236146.62) | 4312.06(3835.80,4880.67) |  | 100.58(94.48,106.79) | -23.83(-26.15,-21.48) | -0.95(-1.09,-0.82) |
| Niue | 35.05(30.02,40.94) | 3428.69(2936.18,4004.99) |  | 18.09(15.72,20.68) | 3425.49(2976.40,3915.80) |  | -48.40(-50.93,-45.49) | -0.09(-5.00,5.53) | -0.14(-0.94,0.66) |
| North Macedonia | 158044.87(131206.48,186478.44) | 22677.12(18826.21,26756.92) |  | 85348.77(69385.37,103671.81) | 19112.60(15537.83,23215.77) |  | -46.00(-49.04,-42.46) | -15.72(-20.47,-10.20) | -0.62(-0.76,-0.48) |
| Northern Mariana Islands | 686.46(591.38,799.06) | 4233.12(3646.80,4927.50) |  | 599.65(516.03,695.07) | 3998.49(3440.96,4634.80) |  | -12.65(-17.09,-8.05) | -5.54(-10.35,-0.57) | -0.01(-0.23,0.20) |
| Norway | 197443.79(169135.31,231467.68) | 17747.06(15202.57,20805.26) |  | 186173.13(157737.69,222017.59) | 14996.37(12705.88,17883.66) |  | -5.71(-9.29,-1.91) | -15.50(-18.71,-12.10) | -0.45(-0.62,-0.29) |
| Oman | 69654.06(60684.37,80086.70) | 6949.45(6054.54,7990.33) |  | 90932.92(78564.32,104183.01) | 6095.38(5266.30,6983.56) |  | 30.55(24.46,36.09) | -12.29(-16.38,-8.57) | -0.52(-0.64,-0.40) |
| Pakistan | 2500317.96(2186303.23,2857560.41) | 4101.35(3586.26,4687.34) |  | 3712446.71(3281872.49,4229586.90) | 3398.94(3004.72,3872.41) |  | 48.48(42.65,53.92) | -17.13(-20.38,-14.09) | -0.67(-0.98,-0.35) |
| Palau | 294.10(258.90,335.36) | 4770.36(4199.38,5439.61) |  | 198.41(174.42,225.39) | 4480.58(3938.85,5089.95) |  | -32.54(-35.93,-29.35) | -6.07(-10.80,-1.64) | -0.15(-0.19,-0.10) |
| Palestine | 83659.76(74061.68,94082.80) | 7009.16(6205.02,7882.42) |  | 147937.12(128590.69,168493.89) | 6143.97(5340.49,6997.71) |  | 76.83(68.67,84.86) | -12.34(-16.39,-8.37) | -0.32(-0.43,-0.21) |
| Panama | 148817.08(126806.41,172596.06) | 13690.21(11665.37,15877.72) |  | 177857.84(149775.60,208877.64) | 11800.30(9937.13,13858.36) |  | 19.51(14.58,25.27) | -13.80(-17.36,-9.65) | -0.52(-0.62,-0.43) |
| Papua New Guinea | 68281.74(60022.88,77404.86) | 3207.76(2819.77,3636.35) |  | 167167.96(149193.70,186231.95) | 3412.25(3045.36,3801.39) |  | 144.82(130.06,158.82) | 6.37(-0.04,12.46) | -0.51(-1.57,0.55) |
| Paraguay | 199340.61(171132.60,232044.35) | 9634.18(8270.88,11214.76) |  | 212952.31(183959.14,248033.21) | 7973.75(6888.13,9287.31) |  | 6.83(1.71,11.98) | -17.23(-21.20,-13.25) | -0.65(-0.78,-0.53) |
| Peru | 969236.15(870598.18,1083353.13) | 9101.06(8174.85,10172.61) |  | 951211.52(832995.45,1084982.02) | 7603.30(6658.37,8672.56) |  | -1.86(-6.58,4.00) | -16.46(-20.47,-11.47) | -0.55(-0.60,-0.51) |
| Philippines | 1780247.34(1567323.27,2027141.67) | 5554.14(4889.85,6324.42) |  | 1644122.67(1430997.84,1880243.94) | 3671.74(3195.78,4199.06) |  | -7.65(-11.14,-4.41) | -33.89(-36.40,-31.57) | -0.81(-1.29,-0.33) |
| Poland | 2679338.29(2191225.42,3187304.25) | 21521.65(17600.91,25601.86) |  | 1248554.76(1007762.05,1499791.86) | 16217.93(13090.19,19481.34) |  | -53.40(-54.87,-52.12) | -24.64(-27.01,-22.57) | -0.98(-1.14,-0.82) |
| Portugal | 528789.28(458536.51,601852.25) | 17738.89(15382.18,20189.88) |  | 215081.98(183827.60,252269.01) | 11221.19(9590.60,13161.30) |  | -59.33(-61.89,-56.60) | -36.74(-40.74,-32.50) | -1.59(-1.70,-1.48) |
| Puerto Rico | 123619.38(104323.74,145176.68) | 9280.14(7831.61,10898.45) |  | 61542.38(51439.45,72436.84) | 9417.60(7871.58,11084.74) |  | -50.22(-52.25,-47.66) | 1.48(-2.67,6.68) | 0.18(-0.06,0.42) |
| Qatar | 9953.79(8649.84,11294.52) | 6502.38(5650.56,7378.22) |  | 35577.27(30470.97,41254.86) | 5958.42(5103.22,6909.29) |  | 257.42(239.81,275.55) | -8.37(-12.88,-3.72) | -0.21(-0.35,-0.07) |
| Republic of Korea | 2933383.66(2561167.36,3346864.27) | 18417.57(16080.57,21013.65) |  | 1066573.79(897394.25,1261861.18) | 12722.66(10704.60,15052.15) |  | -63.64(-66.05,-61.23) | -30.92(-35.50,-26.34) | -1.46(-1.57,-1.35) |
| Republic of Moldova | 284704.64(251083.19,322888.95) | 18004.70(15878.49,20419.48) |  | 84731.81(72256.56,98774.07) | 12169.92(10378.11,14186.79) |  | -70.24(-72.25,-68.46) | -32.41(-36.96,-28.38) | -1.55(-1.68,-1.43) |
| Romania | 1929446.36(1644780.57,2233399.34) | 25716.13(21922.03,29767.29) |  | 766687.83(626824.73,917309.14) | 18973.33(15512.12,22700.77) |  | -60.26(-62.56,-57.82) | -26.22(-30.48,-21.67) | -1.20(-1.28,-1.13) |
| Russian Federation | 9354373.02(7957307.18,10914691.24) | 20702.38(17610.50,24155.55) |  | 4150279.64(3550930.85,4828971.73) | 12282.68(10508.92,14291.26) |  | -55.63(-57.47,-53.73) | -40.67(-43.13,-38.12) | -2.26(-2.54,-1.97) |
| Rwanda | 228079.70(203963.74,255340.20) | 5510.93(4928.24,6169.61) |  | 278137.05(247349.27,315491.64) | 4320.18(3841.97,4900.39) |  | 21.95(17.16,27.35) | -21.61(-24.69,-18.14) | -0.83(-0.92,-0.75) |
| Saint Kitts and Nevis | 1689.99(1466.81,1946.30) | 9199.18(7984.32,10594.37) |  | 1532.66(1335.49,1793.52) | 10899.27(9497.12,12754.32) |  | -9.31(-16.26,-2.21) | 18.48(9.41,27.76) | 0.81(0.61,1.02) |
| Saint Lucia | 5315.26(4552.64,6179.59) | 7959.76(6817.73,9254.12) |  | 3550.42(3058.20,4111.40) | 8461.81(7288.67,9798.80) |  | -33.20(-36.42,-29.36) | 6.31(1.18,12.42) | 0.23(0.05,0.42) |
| Saint Vincent and the Grenadines | 4514.20(3903.32,5180.42) | 8398.06(7261.60,9637.48) |  | 2896.38(2522.48,3376.99) | 8593.51(7484.16,10019.49) |  | -35.84(-39.10,-32.16) | 2.33(-2.87,8.19) | 0.07(-0.11,0.25) |
| Samoa | 3901.77(3448.30,4412.31) | 4259.83(3764.75,4817.23) |  | 2963.83(2560.21,3459.59) | 2936.17(2536.32,3427.31) |  | -24.04(-29.11,-18.81) | -31.07(-35.67,-26.33) | -0.46(-1.82,0.93) |
| San Marino | 986.91(832.82,1175.30) | 16496.58(13920.89,19645.58) |  | 1001.71(835.35,1201.36) | 16055.55(13389.11,19255.50) |  | 1.50(-2.96,5.68) | -2.67(-6.95,1.34) | -0.04(-0.14,0.06) |
| Sao Tome and Principe | 4911.40(4337.20,5521.45) | 7027.92(6206.28,7900.87) |  | 6411.96(5729.61,7152.21) | 6298.39(5628.12,7025.53) |  | 30.55(24.72,37.20) | -10.38(-14.38,-5.82) | -0.57(-0.70,-0.44) |
| Saudi Arabia | 984672.19(877340.15,1103244.40) | 11991.52(10684.41,13435.51) |  | 985303.80(873847.25,1117590.12) | 9723.35(8623.46,11028.81) |  | 0.06(-7.21,8.15) | -18.91(-24.81,-12.36) | -0.58(-0.90,-0.25) |
| Senegal | 258636.85(228357.55,290200.64) | 5816.06(5135.16,6525.85) |  | 370610.41(330336.39,418858.28) | 4566.81(4070.54,5161.34) |  | 43.29(36.68,50.47) | -21.48(-25.10,-17.55) | -0.80(-0.88,-0.73) |
| Serbia | 639004.63(525650.04,751624.07) | 22026.30(18119.00,25908.26) |  | 360452.45(287249.59,435333.32) | 19235.74(15329.23,23231.80) |  | -43.59(-46.84,-39.99) | -12.67(-17.70,-7.09) | -0.38(-0.45,-0.30) |
| Seychelles | 1403.63(1205.18,1625.59) | 4492.80(3857.59,5203.27) |  | 1115.74(962.45,1294.91) | 3654.84(3152.71,4241.74) |  | -20.51(-23.91,-17.28) | -18.65(-22.13,-15.35) | -0.71(-0.94,-0.48) |
| Sierra Leone | 130064.93(116387.68,145248.70) | 5896.75(5276.66,6585.14) |  | 218033.49(196159.20,243858.06) | 4791.25(4310.56,5358.74) |  | 67.63(59.53,75.37) | -18.75(-22.68,-15.00) | -0.72(-1.02,-0.41) |
| Singapore | 139622.56(119314.80,162979.88) | 15160.37(12955.33,17696.54) |  | 143797.01(121390.47,168684.38) | 13781.60(11634.14,16166.82) |  | 2.99(-2.26,8.47) | -9.09(-13.73,-4.26) | -0.30(-0.39,-0.20) |
| Slovakia | 410780.46(338592.20,490856.54) | 23403.49(19290.69,27965.68) |  | 224701.04(183495.68,267188.06) | 20042.88(16367.44,23832.64) |  | -45.30(-47.80,-42.82) | -14.36(-18.28,-10.47) | -0.56(-0.66,-0.47) |
| Slovenia | 156301.47(127549.75,187381.48) | 28072.73(22908.74,33654.90) |  | 95305.53(77063.23,113249.64) | 23542.55(19036.31,27975.14) |  | -39.02(-42.22,-35.84) | -16.14(-20.53,-11.75) | -0.18(-0.61,0.25) |
| Solomon Islands | 6855.93(6125.65,7733.43) | 3522.78(3147.54,3973.66) |  | 12053.25(10819.08,13473.01) | 3644.30(3271.15,4073.56) |  | 75.81(68.03,84.64) | 3.45(-1.13,8.65) | 0.48(-0.12,1.09) |
| Somalia | 275594.40(246247.93,308457.87) | 5823.64(5203.51,6518.08) |  | 672831.78(606886.10,749101.81) | 5262.91(4747.08,5859.50) |  | 144.14(133.40,156.46) | -9.63(-13.60,-5.07) | -0.42(-0.67,-0.17) |
| South Africa | 911399.88(800786.56,1045550.53) | 5172.03(4544.32,5933.31) |  | 720790.91(631748.25,832074.32) | 3618.79(3171.74,4177.50) |  | -20.91(-23.25,-18.28) | -30.03(-32.10,-27.71) | -1.25(-1.47,-1.02) |
| South Sudan | 188851.13(168175.89,214190.48) | 5747.03(5117.85,6518.14) |  | 269929.24(242439.74,304579.45) | 4919.12(4418.16,5550.58) |  | 42.93(36.90,49.52) | -14.41(-18.02,-10.46) | -0.42(-0.52,-0.32) |
| Spain | 1747318.67(1506047.60,2035262.80) | 15675.19(13510.74,18258.33) |  | 1316560.01(1108273.33,1560593.97) | 14948.40(12583.48,17719.19) |  | -24.65(-30.10,-18.12) | -4.64(-11.53,3.63) | -0.06(-0.17,0.04) |
| Sri Lanka | 393830.42(346374.24,443973.54) | 5439.56(4784.10,6132.13) |  | 323954.35(280869.42,372001.35) | 4695.04(4070.61,5391.38) |  | -17.74(-22.33,-13.31) | -13.69(-18.50,-9.04) | -0.76(-1.93,0.42) |
| Sudan | 736621.24(656545.46,823866.74) | 6668.59(5943.67,7458.42) |  | 1178253.02(1053317.52,1317825.14) | 5512.28(4927.79,6165.24) |  | 59.95(50.41,69.66) | -17.34(-22.27,-12.33) | -0.65(-0.72,-0.58) |
| Suriname | 12726.86(10982.91,14651.29) | 7443.01(6423.10,8568.47) |  | 14552.89(12616.65,16806.33) | 7665.34(6645.48,8852.28) |  | 14.35(9.50,20.10) | 2.99(-1.38,8.17) | 0.14(0.02,0.27) |
| Sweden | 394669.49(334816.20,466599.76) | 18731.12(15890.46,22144.95) |  | 379103.49(319260.33,447626.11) | 15695.83(13218.18,18532.84) |  | -3.94(-7.27,-0.87) | -16.20(-19.10,-13.52) | -0.55(-0.66,-0.43) |
| Switzerland | 382410.99(328603.35,442649.78) | 24246.64(20834.98,28066.06) |  | 326278.38(272481.61,386175.08) | 18511.28(15459.14,21909.50) |  | -14.68(-20.91,-8.26) | -23.65(-29.23,-17.91) | -1.11(-1.41,-0.80) |
| Syrian Arab Republic | 483286.60(424023.41,553012.66) | 6573.60(5767.51,7522.01) |  | 366058.40(311960.68,424176.42) | 6728.89(5734.47,7797.22) |  | -24.26(-28.93,-19.42) | 2.36(-3.95,8.90) | 0.06(-0.06,0.18) |
| Taiwan (Province of China) | 237823.11(208503.57,271640.43) | 3253.86(2852.72,3716.55) |  | 81693.19(70487.97,96399.97) | 2019.38(1742.40,2382.92) |  | -65.65(-67.92,-63.42) | -37.94(-42.04,-33.91) | -1.96(-2.49,-1.43) |
| Tajikistan | 339404.83(295150.80,384484.03) | 11807.52(10267.97,13375.78) |  | 391783.55(335134.66,452851.46) | 8793.98(7522.44,10164.71) |  | 15.43(10.53,20.33) | -25.52(-28.69,-22.36) | -1.22(-1.40,-1.04) |
| Thailand | 1200157.50(1053703.49,1364619.49) | 5276.15(4632.31,5999.16) |  | 566457.25(491471.76,648648.35) | 4150.42(3601.00,4752.63) |  | -52.80(-55.28,-50.01) | -21.34(-25.46,-16.69) | -0.83(-1.10,-0.55) |
| Timor-Leste | 18250.48(16154.97,20634.41) | 4487.85(3972.56,5074.07) |  | 27009.97(24245.93,30490.08) | 3940.59(3537.33,4448.31) |  | 48.00(40.15,56.88) | -12.19(-16.85,-6.92) | -0.83(-0.97,-0.70) |
| Togo | 119922.89(107189.10,135316.70) | 5570.75(4979.23,6285.84) |  | 183257.81(164346.85,207014.80) | 4388.98(3936.07,4957.95) |  | 52.81(47.18,59.21) | -21.21(-24.12,-17.91) | -0.81(-0.88,-0.75) |
| Tokelau | 25.80(21.88,30.03) | 3401.41(2884.52,3960.09) |  | 15.78(13.42,18.62) | 3083.84(2624.15,3639.97) |  | -38.84(-42.31,-35.46) | -9.34(-14.48,-4.31) | -0.33(-0.47,-0.19) |
| Tonga | 1839.30(1592.10,2137.85) | 3446.82(2983.58,4006.30) |  | 1384.09(1185.80,1606.22) | 2791.48(2391.57,3239.49) |  | -24.75(-28.21,-21.03) | -19.01(-22.74,-15.01) | -0.44(-0.94,0.06) |
| Trinidad and Tobago | 41082.64(35202.51,47971.76) | 7908.51(6776.57,9234.69) |  | 27557.08(23307.40,32217.47) | 7570.96(6403.42,8851.35) |  | -32.92(-35.52,-30.23) | -4.27(-7.98,-0.43) | -0.09(-0.20,0.02) |
| Tunisia | 346382.51(304359.51,395310.31) | 8681.15(7627.95,9907.39) |  | 238322.59(207236.84,271200.23) | 6646.74(5779.76,7563.68) |  | -31.20(-35.99,-26.31) | -23.43(-28.77,-17.99) | -0.97(-1.06,-0.89) |
| Turkey | 1966890.39(1732408.42,2226060.32) | 7371.71(6492.89,8343.05) |  | 1484745.39(1278115.03,1730921.24) | 6008.15(5172.00,7004.32) |  | -24.51(-29.40,-19.29) | -18.50(-23.78,-12.86) | -0.73(-0.91,-0.56) |
| Turkmenistan | 243232.80(212370.10,276282.24) | 12902.00(11264.92,14655.07) |  | 201888.95(172901.03,234065.82) | 10297.55(8818.99,11938.76) |  | -17.00(-21.66,-11.82) | -20.19(-24.67,-15.20) | -0.72(-0.77,-0.67) |
| Tuvalu | 135.23(119.52,153.79) | 3164.72(2796.92,3598.97) |  | 134.63(115.50,157.72) | 2740.32(2351.08,3210.38) |  | -0.45(-5.68,5.20) | -13.41(-17.96,-8.50) | -1.61(-2.14,-1.07) |
| Uganda | 542217.02(483611.36,614688.90) | 5265.37(4696.27,5969.14) |  | 1097131.65(981144.19,1252638.60) | 4398.46(3933.46,5021.90) |  | 102.34(95.10,110.73) | -16.46(-19.46,-13.00) | -0.58(-0.68,-0.48) |
| Ukraine | 2661703.62(2281554.96,3083397.34) | 17683.75(15158.13,20485.39) |  | 1165252.17(1005226.97,1346817.39) | 13797.77(11902.91,15947.68) |  | -56.22(-58.63,-53.88) | -21.97(-26.26,-17.81) | -1.20(-1.33,-1.07) |
| United Arab Emirates | 51204.56(44982.95,58200.88) | 7242.32(6362.34,8231.87) |  | 101085.73(87581.49,117186.75) | 5963.47(5166.80,6913.34) |  | 97.42(87.46,107.62) | -17.66(-21.81,-13.40) | -0.84(-0.99,-0.69) |
| United Kingdom | 2472002.55(2117939.29,2893771.93) | 16664.49(14277.65,19507.76) |  | 2170357.41(1835533.13,2577510.77) | 13863.95(11725.14,16464.79) |  | -12.20(-14.61,-9.82) | -16.81(-19.08,-14.55) | -0.57(-0.72,-0.41) |
| United Republic of Tanzania | 866191.33(769522.87,981913.64) | 5804.32(5156.55,6579.77) |  | 1494558.48(1324657.82,1689085.82) | 4843.30(4292.72,5473.69) |  | 72.54(66.75,78.75) | -16.56(-19.36,-13.55) | -0.53(-0.59,-0.47) |
| United States of America | 9682904.15(8249805.67,11290062.83) | 13076.99(11141.56,15247.49) |  | 6363103.48(5421438.96,7433922.66) | 7825.51(6667.42,9142.43) |  | -34.29(-37.17,-30.36) | -40.16(-42.78,-36.59) | -1.92(-2.68,-1.15) |
| United States Virgin Islands | 3628.82(3098.37,4221.29) | 8699.08(7427.47,10119.37) |  | 1476.41(1220.16,1767.83) | 8103.47(6696.99,9702.98) |  | -59.31(-61.10,-56.87) | -6.85(-10.93,-1.24) | -0.33(-0.55,-0.11) |
| Uruguay | 294441.73(249055.88,354902.59) | 27338.42(23124.42,32952.11) |  | 196006.06(165641.05,231844.74) | 21698.20(18336.74,25665.60) |  | -33.43(-38.73,-28.11) | -20.63(-26.95,-14.28) | -0.89(-0.94,-0.83) |
| Uzbekistan | 1295805.41(1128579.78,1476794.81) | 12137.77(10571.37,13833.09) |  | 1158774.47(997314.49,1348094.94) | 9189.23(7908.83,10690.57) |  | -10.57(-14.26,-6.41) | -24.29(-27.41,-20.77) | -0.99(-1.17,-0.81) |
| Vanuatu | 2574.23(2227.32,2983.12) | 3084.82(2669.10,3574.82) |  | 3977.18(3474.82,4562.44) | 2697.52(2356.79,3094.46) |  | 54.50(46.15,62.45) | -12.56(-17.28,-8.05) | -0.60(-1.29,0.10) |
| Venezuela (Bolivarian Republic of) | 1552575.14(1332159.50,1819814.30) | 17106.24(14677.70,20050.67) |  | 1264274.25(1067029.81,1486689.06) | 14445.88(12192.13,16987.25) |  | -18.57(-22.18,-14.79) | -15.55(-19.29,-11.63) | -0.71(-1.09,-0.33) |
| Viet Nam | 1730311.19(1516350.47,1980291.14) | 5139.50(4503.98,5882.01) |  | 1454349.19(1272226.51,1658934.96) | 4599.62(4023.63,5246.65) |  | -15.95(-19.87,-11.59) | -10.50(-14.68,-5.86) | -0.32(-0.45,-0.19) |
| Yemen | 488290.28(429923.69,552232.10) | 5807.39(5113.21,6567.87) |  | 841974.75(746104.28,950895.30) | 4818.96(4270.26,5442.36) |  | 72.43(61.37,84.80) | -17.02(-22.34,-11.07) | -0.64(-0.71,-0.56) |
| Zambia | 251695.92(224861.29,283672.00) | 5354.67(4783.78,6034.94) |  | 450150.13(401378.33,508886.51) | 4305.91(3839.39,4867.76) |  | 78.85(72.16,86.14) | -19.59(-22.59,-16.31) | -0.75(-0.84,-0.65) |
| Zimbabwe | 255963.04(224428.87,293555.53) | 4250.21(3726.59,4874.42) |  | 306242.84(275039.39,348605.54) | 3835.59(3444.78,4366.17) |  | 19.64(13.78,25.86) | -9.76(-14.18,-5.07) | -0.27(-0.36,-0.18) |
