# Supplementary material for: Global patterns and health impact of unintentional injuries among children and adolescents, 1990–2021
Source: Front Public Health. 2025 Sep 24;13:1626739. doi: 10.3389/fpubh.2025.1626739 (PMC12504300; doi:10.3389/fpubh.2025.1626739)
Supplement: Supplementary file 4 [file Table_3.DOCX]

Table S3. Mortality of Unintentional Injuries at the National Level.

| location | 1990 | |  | 2021 | |  | 1990-2021 | | |
| --- | --- | --- | --- | --- | --- | --- | --- | --- | --- |
|  | Death cases | Death rate |  | Death cases | Death rate |  | Cases change | Rate Change | EAPC |
| Afghanistan | 3641.76(2543.52,4910.55) | 65.35(45.64,88.11) |  | 5294.90(3942.64,7090.64) | 29.79(22.18,39.89) |  | 45.39(3.63,106.98) | -54.42(-67.51,-35.11) | -2.86(-3.41,-2.31) |
| Albania | 264.41(223.28,306.04) | 18.29(15.44,21.17) |  | 29.46(23.38,36.37) | 4.77(3.79,5.89) |  | -88.86(-91.20,-85.92) | -73.89(-79.38,-67.01) | -4.32(-4.66,-3.98) |
| Algeria | 3138.74(2577.41,3745.59) | 23.22(19.07,27.71) |  | 1059.14(856.00,1295.50) | 6.44(5.20,7.88) |  | -66.26(-72.87,-58.71) | -72.27(-77.71,-66.07) | -3.80(-4.16,-3.44) |
| American Samoa | 2.82(2.38,3.25) | 11.77(9.93,13.58) |  | 1.70(1.37,2.10) | 8.88(7.16,10.97) |  | -39.64(-53.37,-22.63) | -24.50(-41.67,-3.22) | -0.61(-2.10,0.91) |
| Andorra | 0.14(0.09,0.20) | 1.03(0.69,1.48) |  | 0.05(0.04,0.07) | 0.36(0.26,0.49) |  | -62.41(-78.18,-34.37) | -64.82(-79.57,-38.56) | -3.30(-3.45,-3.15) |
| Angola | 4764.89(3697.87,6117.22) | 82.64(64.13,106.09) |  | 3438.41(2291.02,4842.31) | 18.41(12.27,25.93) |  | -27.84(-54.84,17.39) | -77.72(-86.06,-63.76) | -4.71(-5.07,-4.35) |
| Antigua and Barbuda | 2.97(2.53,3.43) | 12.43(10.60,14.38) |  | 1.92(1.74,2.14) | 8.22(7.44,9.14) |  | -35.31(-45.71,-22.40) | -33.85(-44.49,-20.65) | -2.13(-3.21,-1.03) |
| Argentina | 2594.50(2494.60,2705.31) | 19.92(19.15,20.77) |  | 686.14(614.35,757.60) | 5.00(4.48,5.53) |  | -73.55(-76.33,-70.56) | -74.87(-77.51,-72.03) | -4.25(-4.42,-4.08) |
| Armenia | 378.85(339.24,424.27) | 28.53(25.55,31.95) |  | 50.15(40.97,62.58) | 6.57(5.37,8.20) |  | -86.76(-89.57,-83.20) | -76.97(-81.85,-70.78) | -4.64(-5.06,-4.22) |
| Australia | 284.21(272.47,296.71) | 5.50(5.27,5.74) |  | 100.99(89.61,114.09) | 1.62(1.44,1.83) |  | -64.47(-68.92,-59.68) | -70.54(-74.23,-66.57) | -3.28(-3.52,-3.04) |
| Austria | 94.32(89.30,99.03) | 5.04(4.77,5.29) |  | 27.17(24.51,29.64) | 1.55(1.40,1.69) |  | -71.20(-74.04,-68.26) | -69.26(-72.29,-66.12) | -3.37(-3.64,-3.10) |
| Azerbaijan | 903.50(767.64,1040.83) | 28.86(24.52,33.25) |  | 290.83(236.26,361.58) | 9.55(7.75,11.87) |  | -67.81(-75.46,-58.06) | -66.93(-74.78,-56.92) | -3.72(-3.94,-3.51) |
| Bahamas | 19.38(16.94,22.31) | 18.00(15.74,20.73) |  | 8.24(6.53,10.67) | 7.15(5.66,9.26) |  | -57.46(-66.83,-44.69) | -60.27(-69.02,-48.34) | -1.27(-3.06,0.55) |
| Bahrain | 17.84(15.98,19.65) | 8.92(7.99,9.82) |  | 11.13(9.46,12.82) | 2.76(2.34,3.18) |  | -37.61(-47.88,-26.27) | -69.06(-74.15,-63.44) | -3.56(-3.78,-3.33) |
| Bangladesh | 50459.43(41650.50,59628.99) | 83.60(69.00,98.79) |  | 9379.13(7625.02,11457.41) | 15.38(12.50,18.79) |  | -81.41(-85.66,-75.55) | -81.60(-85.81,-75.80) | -5.72(-6.40,-5.03) |
| Barbados | 8.37(7.39,9.37) | 9.86(8.71,11.04) |  | 2.82(2.00,3.82) | 4.26(3.01,5.76) |  | -66.26(-75.92,-53.02) | -56.85(-69.20,-39.90) | -2.34(-2.72,-1.95) |
| Belarus | 673.62(601.92,748.91) | 21.47(19.18,23.87) |  | 108.58(91.16,128.59) | 5.36(4.50,6.35) |  | -83.88(-86.46,-80.97) | -75.02(-79.02,-70.52) | -4.82(-5.32,-4.32) |
| Belgium | 148.71(141.56,156.01) | 6.02(5.73,6.31) |  | 58.37(51.89,65.74) | 2.29(2.04,2.58) |  | -60.75(-65.27,-55.19) | -61.87(-66.26,-56.47) | -2.91(-3.33,-2.50) |
| Belize | 34.65(31.19,38.56) | 33.78(30.40,37.59) |  | 14.96(12.92,17.18) | 8.85(7.64,10.16) |  | -56.82(-64.33,-48.75) | -73.80(-78.35,-68.90) | -3.98(-4.40,-3.56) |
| Benin | 1398.42(1026.48,1726.90) | 48.73(35.77,60.18) |  | 1955.67(1120.16,2700.47) | 26.02(14.90,35.93) |  | 39.85(-19.84,119.24) | -46.61(-69.40,-16.30) | -1.71(-1.92,-1.50) |
| Bermuda | 1.20(1.04,1.38) | 7.52(6.53,8.66) |  | 0.36(0.29,0.44) | 3.15(2.50,3.83) |  | -69.74(-76.21,-61.01) | -58.15(-67.10,-46.07) | -2.72(-3.38,-2.05) |
| Bhutan | 94.17(46.00,123.62) | 27.65(13.50,36.29) |  | 31.19(23.93,41.20) | 12.23(9.38,16.16) |  | -66.88(-76.82,-34.56) | -55.75(-69.04,-12.58) | -3.49(-4.22,-2.76) |
| Bolivia (Plurinational State of) | 2080.96(1701.42,2555.86) | 62.14(50.81,76.32) |  | 768.98(624.82,973.38) | 16.91(13.74,21.41) |  | -63.05(-70.95,-52.62) | -72.78(-78.60,-65.10) | -4.16(-4.24,-4.08) |
| Bosnia and Herzegovina | 110.77(81.43,131.31) | 7.48(5.50,8.86) |  | 10.15(8.52,12.22) | 1.53(1.28,1.84) |  | -90.84(-92.94,-86.90) | -79.59(-84.27,-70.82) | -4.25(-4.74,-3.77) |
| Botswana | 137.68(109.83,168.40) | 18.59(14.83,22.74) |  | 133.94(102.02,173.20) | 14.61(11.13,18.89) |  | -2.72(-25.49,24.24) | -21.45(-39.83,0.32) | -0.22(-0.52,0.09) |
| Brazil | 10850.75(9906.85,11794.27) | 16.15(14.74,17.55) |  | 3604.58(3081.92,4138.30) | 5.64(4.82,6.47) |  | -66.78(-71.66,-61.19) | -65.08(-70.21,-59.20) | -2.86(-3.10,-2.61) |
| Brunei Darussalam | 11.78(10.31,13.33) | 10.24(8.96,11.59) |  | 5.26(4.37,6.47) | 4.07(3.39,5.01) |  | -55.37(-64.06,-43.18) | -60.24(-67.99,-49.39) | -2.33(-2.64,-2.01) |
| Bulgaria | 368.22(340.66,388.18) | 15.58(14.41,16.43) |  | 54.48(46.71,62.15) | 4.21(3.61,4.80) |  | -85.20(-87.34,-82.91) | -72.98(-76.88,-68.80) | -4.44(-4.72,-4.16) |
| Burkina Faso | 3125.62(2326.12,3880.46) | 55.25(41.12,68.59) |  | 3963.00(2463.62,5841.77) | 30.95(19.24,45.62) |  | 26.79(-16.03,89.69) | -43.99(-62.90,-16.20) | -1.45(-1.63,-1.26) |
| Burundi | 1488.71(1111.38,1832.70) | 47.18(35.22,58.09) |  | 1159.94(696.73,1855.68) | 15.95(9.58,25.51) |  | -22.08(-46.86,21.04) | -66.21(-76.95,-47.50) | -2.78(-3.15,-2.42) |
| Cabo Verde | 28.85(20.52,37.80) | 14.84(10.55,19.44) |  | 21.01(16.98,24.99) | 10.90(8.81,12.97) |  | -27.20(-48.91,7.14) | -26.55(-48.46,8.10) | -1.95(-2.61,-1.28) |
| Cambodia | 2538.83(1997.28,3123.30) | 44.59(35.08,54.86) |  | 1011.48(786.70,1264.80) | 15.25(11.86,19.07) |  | -60.16(-70.13,-45.67) | -65.79(-74.35,-53.36) | -3.73(-3.94,-3.53) |
| Cameroon | 1863.58(1347.62,2288.79) | 31.39(22.70,38.55) |  | 3047.07(1741.31,4094.77) | 18.03(10.30,24.23) |  | 63.51(3.00,127.21) | -42.57(-63.82,-20.19) | -1.30(-1.63,-0.97) |
| Canada | 447.64(432.77,463.53) | 5.84(5.65,6.05) |  | 175.64(158.10,194.46) | 2.13(1.92,2.36) |  | -60.76(-64.93,-55.86) | -63.45(-67.33,-58.89) | -2.70(-2.89,-2.51) |
| Central African Republic | 1073.96(831.15,1327.79) | 71.73(55.52,88.69) |  | 1103.21(788.15,1502.95) | 38.12(27.23,51.93) |  | 2.72(-28.75,46.71) | -46.86(-63.14,-24.11) | -1.71(-1.87,-1.54) |
| Chad | 1580.53(1115.06,2078.89) | 44.83(31.63,58.97) |  | 3987.20(2708.64,5201.70) | 36.41(24.74,47.51) |  | 152.27(71.46,256.72) | -18.78(-44.80,14.85) | -0.50(-0.72,-0.28) |
| Chile | 1166.59(1112.41,1226.04) | 22.12(21.09,23.24) |  | 162.08(148.73,175.65) | 3.31(3.04,3.59) |  | -86.11(-87.43,-84.76) | -85.03(-86.45,-83.57) | -5.54(-6.18,-4.89) |
| China | 225740.19(197101.71,259544.22) | 50.72(44.29,58.32) |  | 30017.00(25973.16,34548.09) | 8.98(7.77,10.33) |  | -86.70(-89.03,-84.22) | -82.30(-85.40,-78.99) | -5.39(-5.65,-5.12) |
| Colombia | 2388.79(2164.34,2597.25) | 15.87(14.38,17.25) |  | 645.45(525.92,790.27) | 4.43(3.61,5.43) |  | -72.98(-78.45,-66.64) | -72.06(-77.72,-65.51) | -3.39(-3.80,-2.98) |
| Comoros | 85.71(58.94,112.62) | 32.43(22.30,42.61) |  | 46.08(36.35,58.77) | 14.68(11.58,18.72) |  | -46.24(-60.52,-20.11) | -54.74(-66.76,-32.74) | -2.58(-2.85,-2.30) |
| Congo | 458.34(363.95,575.30) | 34.55(27.43,43.36) |  | 302.15(213.49,421.41) | 12.15(8.58,16.95) |  | -34.08(-52.04,-6.99) | -64.83(-74.41,-50.38) | -3.49(-3.89,-3.10) |
| Cook Islands | 1.08(0.95,1.23) | 12.59(11.07,14.27) |  | 0.13(0.11,0.16) | 2.54(2.11,3.05) |  | -87.88(-90.15,-84.70) | -79.86(-83.64,-74.59) | -5.73(-7.77,-3.65) |
| Costa Rica | 148.50(141.91,156.02) | 10.47(10.01,11.00) |  | 48.94(43.71,54.09) | 3.57(3.19,3.95) |  | -67.04(-70.71,-63.22) | -65.86(-69.66,-61.90) | -3.34(-3.60,-3.09) |
| Croatia | 2247.96(1733.28,2776.62) | 32.49(25.05,40.13) |  | 2721.21(1677.93,3809.41) | 18.92(11.66,26.48) |  | -87.09(-88.96,-84.93) | -78.75(-81.83,-75.19) | -1.25(-1.51,-0.98) |
| Cuba | 112.29(105.22,119.45) | 8.47(7.93,9.01) |  | 14.49(12.55,16.62) | 1.80(1.56,2.06) |  | -76.54(-79.67,-73.24) | -64.31(-69.09,-59.29) | -5.09(-5.36,-4.82) |
| Cyprus | 427.22(408.60,446.68) | 11.70(11.19,12.23) |  | 100.24(87.35,114.14) | 4.17(3.64,4.75) |  | -75.96(-80.17,-70.79) | -78.16(-81.98,-73.45) | -3.19(-3.63,-2.76) |
| Czechia | 20.30(17.53,23.27) | 7.83(6.77,8.98) |  | 4.88(4.13,5.80) | 1.71(1.45,2.03) |  | -85.46(-87.54,-83.24) | -80.07(-82.91,-77.03) | -4.40(-4.80,-3.99) |
| C么te d'Ivoire | 288.99(274.47,305.19) | 9.49(9.02,10.03) |  | 42.01(36.59,48.04) | 1.89(1.65,2.16) |  | 21.05(-17.29,65.07) | -41.78(-60.22,-20.61) | -5.21(-5.45,-4.97) |
| Democratic People's Republic of Korea | 1906.32(1372.75,2556.01) | 24.47(17.62,32.81) |  | 741.40(575.77,976.36) | 11.23(8.72,14.79) |  | -61.11(-71.83,-43.96) | -54.12(-66.76,-33.88) | -2.13(-2.45,-1.81) |
| Democratic Republic of the Congo | 11830.52(9010.76,14734.99) | 54.64(41.62,68.06) |  | 7842.21(5133.80,11552.52) | 16.37(10.72,24.11) |  | -33.71(-51.65,-6.71) | -70.04(-78.15,-57.84) | -3.31(-3.66,-2.95) |
| Denmark | 45.49(43.02,48.22) | 3.64(3.44,3.86) |  | 14.14(12.47,15.92) | 1.09(0.96,1.23) |  | -68.91(-72.48,-64.49) | -69.97(-73.41,-65.69) | -4.09(-4.42,-3.75) |
| Djibouti | 59.28(45.29,75.17) | 26.43(20.19,33.52) |  | 66.18(45.66,95.67) | 12.53(8.64,18.11) |  | 11.65(-22.63,60.13) | -52.61(-67.16,-32.03) | -2.51(-3.25,-1.76) |
| Dominica | 4.62(3.78,5.47) | 14.11(11.54,16.68) |  | 2.20(1.81,2.67) | 11.30(9.30,13.75) |  | -52.51(-63.33,-36.91) | -19.89(-38.14,6.44) | 0.90(-0.89,2.73) |
| Dominican Republic | 960.13(796.86,1106.10) | 27.42(22.75,31.58) |  | 359.15(279.55,448.93) | 9.23(7.19,11.54) |  | -62.59(-71.20,-52.40) | -66.33(-74.08,-57.15) | -3.30(-3.76,-2.83) |
| Ecuador | 1160.37(1080.79,1247.86) | 23.41(21.80,25.17) |  | 582.81(473.77,718.57) | 8.81(7.16,10.86) |  | -49.77(-59.20,-38.83) | -62.37(-69.43,-54.16) | -3.02(-3.34,-2.69) |
| Egypt | 6891.04(5731.04,8023.34) | 24.68(20.52,28.73) |  | 2938.14(2359.29,3609.46) | 6.31(5.07,7.75) |  | -57.36(-67.11,-45.22) | -74.42(-80.27,-67.14) | -3.61(-3.89,-3.32) |
| El Salvador | 611.86(494.67,710.03) | 22.21(17.95,25.77) |  | 139.11(110.92,175.95) | 5.84(4.66,7.39) |  | -77.26(-82.67,-68.74) | -73.70(-79.95,-63.84) | -4.18(-4.98,-3.37) |
| Equatorial Guinea | 138.29(105.30,177.46) | 57.63(43.88,73.95) |  | 103.95(63.45,159.71) | 13.50(8.24,20.74) |  | -24.83(-52.83,22.82) | -76.58(-85.30,-61.73) | -6.28(-6.75,-5.80) |
| Eritrea | 786.59(585.82,1019.87) | 39.99(29.79,51.85) |  | 658.01(465.37,928.68) | 20.46(14.47,28.87) |  | -16.35(-45.66,35.82) | -48.85(-66.77,-16.95) | -1.99(-2.16,-1.82) |
| Estonia | 125.76(119.40,132.18) | 27.38(26.00,28.78) |  | 9.41(8.28,10.59) | 3.36(2.95,3.78) |  | -92.51(-93.50,-91.42) | -87.73(-89.35,-85.93) | -6.93(-7.26,-6.60) |
| Eswatini | 112.20(86.40,137.38) | 23.52(18.11,28.79) |  | 93.58(70.90,116.98) | 17.49(13.25,21.87) |  | -16.59(-37.28,13.35) | -25.62(-44.06,1.09) | -0.40(-0.60,-0.20) |
| Ethiopia | 16613.80(12191.81,21277.95) | 56.38(41.37,72.21) |  | 9169.69(6643.95,13280.42) | 16.03(11.62,23.22) |  | -44.81(-61.93,-14.19) | -71.57(-80.39,-55.79) | -4.27(-4.41,-4.14) |
| Fiji | 48.98(39.64,60.36) | 13.65(11.05,16.83) |  | 47.27(36.64,60.32) | 13.48(10.45,17.20) |  | -3.49(-31.09,34.78) | -1.26(-29.50,37.89) | -0.15(-0.60,0.31) |
| Finland | 74.15(70.13,78.29) | 5.84(5.53,6.17) |  | 28.08(25.00,30.82) | 2.45(2.18,2.68) |  | -62.13(-66.75,-57.51) | -58.16(-63.26,-53.06) | -2.92(-3.18,-2.66) |
| France | 1153.60(1114.76,1195.69) | 7.15(6.91,7.41) |  | 380.61(347.56,413.22) | 2.42(2.21,2.62) |  | -67.01(-70.24,-63.90) | -66.19(-69.50,-63.00) | -3.23(-3.59,-2.87) |
| Gabon | 121.20(92.52,154.31) | 23.85(18.21,30.37) |  | 87.75(53.29,133.86) | 10.61(6.44,16.19) |  | -27.60(-52.83,7.00) | -55.51(-71.01,-34.25) | -1.97(-2.25,-1.69) |
| Gambia | 184.71(134.75,235.98) | 32.88(23.99,42.01) |  | 197.17(139.27,273.89) | 15.57(11.00,21.62) |  | 6.75(-19.89,46.15) | -52.65(-64.47,-35.18) | -2.69(-3.04,-2.34) |
| Georgia | 419.43(376.91,464.76) | 23.34(20.98,25.87) |  | 57.50(48.11,68.43) | 6.19(5.18,7.36) |  | -86.29(-88.43,-83.48) | -73.49(-77.62,-68.07) | -3.93(-4.52,-3.33) |
| Germany | 903.55(870.71,939.20) | 5.21(5.02,5.42) |  | 205.64(193.32,218.20) | 1.29(1.21,1.37) |  | -77.24(-78.85,-75.53) | -75.27(-77.01,-73.40) | -4.19(-4.58,-3.80) |
| Ghana | 1839.08(1438.58,2274.40) | 22.27(17.42,27.54) |  | 1788.32(1130.00,2635.55) | 10.96(6.93,16.16) |  | -2.76(-35.88,40.87) | -50.77(-67.54,-28.68) | -1.60(-1.85,-1.35) |
| Greece | 134.44(127.16,141.22) | 4.78(4.52,5.02) |  | 39.09(34.87,42.92) | 2.05(1.83,2.25) |  | -70.92(-74.14,-67.58) | -57.10(-61.85,-52.18) | -2.34(-2.61,-2.06) |
| Greenland | 5.53(4.67,6.54) | 30.93(26.11,36.55) |  | 1.52(1.24,1.84) | 9.87(8.08,11.96) |  | -72.59(-78.40,-65.77) | -68.09(-74.85,-60.15) | -3.80(-4.15,-3.44) |
| Grenada | 9.24(7.84,10.88) | 21.91(18.60,25.81) |  | 2.64(2.33,2.97) | 8.77(7.73,9.86) |  | -71.41(-75.88,-66.40) | -59.97(-66.22,-52.94) | -2.32(-2.95,-1.70) |
| Guam | 3.70(3.22,4.27) | 6.83(5.95,7.88) |  | 1.90(1.52,2.34) | 3.92(3.14,4.83) |  | -48.56(-59.95,-34.92) | -42.56(-55.28,-27.33) | -1.16(-1.63,-0.69) |
| Guatemala | 1650.23(1530.73,1792.81) | 33.60(31.17,36.51) |  | 652.99(538.87,776.85) | 9.87(8.15,11.74) |  | -60.43(-67.39,-51.98) | -70.62(-75.79,-64.35) | -3.12(-3.65,-2.59) |
| Guinea | 2039.60(1477.41,2614.77) | 62.05(44.94,79.54) |  | 2004.11(1244.37,2701.46) | 26.85(16.67,36.19) |  | -1.74(-39.70,48.83) | -56.73(-73.45,-34.46) | -2.07(-2.33,-1.82) |
| Guinea-Bissau | 340.55(249.13,456.62) | 57.91(42.36,77.64) |  | 232.26(153.65,325.33) | 20.76(13.74,29.09) |  | -31.80(-52.37,2.12) | -64.14(-74.96,-46.31) | -2.90(-3.32,-2.47) |
| Guyana | 107.01(90.10,124.07) | 28.04(23.61,32.51) |  | 37.15(29.28,46.28) | 13.23(10.43,16.49) |  | -65.28(-74.06,-54.12) | -52.81(-64.74,-37.63) | -1.10(-1.56,-0.64) |
| Haiti | 3241.35(2415.01,4015.33) | 96.86(72.17,119.99) |  | 3469.06(2852.06,4111.61) | 61.86(50.86,73.32) |  | 7.03(-12.59,33.62) | -36.13(-47.84,-20.26) | -1.71(-4.49,1.15) |
| Honduras | 788.16(656.39,937.48) | 29.04(24.19,34.55) |  | 314.36(226.00,461.50) | 7.23(5.19,10.61) |  | -60.11(-70.90,-43.11) | -75.12(-81.85,-64.51) | -5.20(-6.95,-3.42) |
| Hungary | 256.41(243.90,269.67) | 8.83(8.40,9.29) |  | 39.90(33.72,46.12) | 2.13(1.80,2.46) |  | -84.44(-86.88,-81.96) | -75.92(-79.69,-72.08) | -4.15(-4.34,-3.96) |
| Iceland | 3.67(3.37,3.97) | 4.35(3.99,4.70) |  | 1.26(1.08,1.45) | 1.41(1.21,1.63) |  | -65.82(-70.86,-60.03) | -67.58(-72.36,-62.09) | -3.97(-5.39,-2.53) |
| India | 133181.79(107866.17,160062.24) | 32.45(26.28,39.00) |  | 51952.47(38458.49,65677.49) | 10.38(7.68,13.12) |  | -60.99(-70.14,-48.12) | -68.02(-75.52,-57.46) | -3.56(-3.84,-3.28) |
| Indonesia | 19867.32(16479.24,23108.94) | 22.72(18.84,26.42) |  | 8333.77(6586.06,10246.98) | 9.24(7.30,11.36) |  | -58.05(-67.53,-46.89) | -59.33(-68.52,-48.51) | -2.75(-4.07,-1.41) |
| Iran (Islamic Republic of) | 35873.78(33108.71,38946.85) | 113.85(105.08,123.60) |  | 1388.47(1253.63,1559.79) | 5.37(4.85,6.03) |  | -96.13(-96.64,-95.54) | -95.28(-95.90,-94.57) | -5.78(-6.93,-4.63) |
| Iraq | 3845.27(3145.03,4609.85) | 37.34(30.54,44.76) |  | 2053.48(1698.47,2490.09) | 11.64(9.63,14.12) |  | -46.60(-57.75,-30.43) | -68.82(-75.33,-59.38) | -3.77(-4.03,-3.50) |
| Ireland | 60.07(56.67,63.42) | 4.52(4.26,4.77) |  | 14.08(12.42,15.76) | 1.07(0.94,1.19) |  | -76.55(-79.48,-73.61) | -76.43(-79.37,-73.46) | -4.28(-4.72,-3.83) |
| Israel | 121.67(114.99,128.00) | 6.06(5.73,6.38) |  | 49.48(44.28,55.25) | 1.47(1.31,1.64) |  | -59.33(-64.37,-54.18) | -75.81(-78.81,-72.75) | -3.63(-3.94,-3.32) |
| Italy | 527.13(514.64,539.02) | 3.87(3.78,3.96) |  | 105.37(95.76,114.58) | 1.01(0.91,1.09) |  | -80.01(-81.75,-78.30) | -74.02(-76.28,-71.80) | -4.34(-4.69,-3.99) |
| Jamaica | 84.89(76.43,95.35) | 7.77(7.00,8.73) |  | 20.55(16.26,26.49) | 2.52(1.99,3.25) |  | -75.79(-80.97,-69.30) | -67.56(-74.50,-58.87) | -3.70(-4.10,-3.31) |
| Japan | 1493.13(1457.72,1531.82) | 4.48(4.37,4.59) |  | 368.29(340.03,394.86) | 1.73(1.60,1.86) |  | -75.33(-77.37,-73.33) | -61.24(-64.45,-58.10) | -3.01(-4.08,-1.92) |
| Jordan | 294.88(259.51,333.47) | 14.06(12.38,15.91) |  | 233.72(197.33,277.79) | 4.75(4.01,5.64) |  | -20.74(-35.08,-1.46) | -66.26(-72.36,-58.05) | -3.76(-4.04,-3.47) |
| Kazakhstan | 2248.23(2039.27,2491.55) | 33.84(30.70,37.51) |  | 706.80(600.18,836.46) | 10.51(8.93,12.44) |  | -68.56(-73.92,-62.62) | -68.94(-74.24,-63.07) | -3.56(-3.93,-3.19) |
| Kenya | 2512.66(2081.94,3025.30) | 18.22(15.10,21.94) |  | 1952.19(1534.10,2531.63) | 7.94(6.24,10.29) |  | -22.31(-38.67,-0.91) | -56.44(-65.62,-44.44) | -1.60(-1.96,-1.24) |
| Kiribati | 4.04(3.15,4.99) | 11.03(8.60,13.61) |  | 3.22(2.37,4.15) | 6.02(4.43,7.76) |  | -20.49(-40.62,5.65) | -45.47(-59.28,-27.54) | -1.79(-1.91,-1.68) |
| Kuwait | 68.95(63.57,75.35) | 10.05(9.27,10.98) |  | 33.85(28.17,40.14) | 3.09(2.57,3.66) |  | -50.91(-59.67,-40.64) | -69.26(-74.75,-62.83) | -3.36(-3.69,-3.02) |
| Kyrgyzstan | 706.88(637.26,785.42) | 33.48(30.18,37.20) |  | 234.85(209.85,259.40) | 8.32(7.43,9.19) |  | -66.78(-70.84,-61.60) | -75.15(-78.19,-71.28) | -4.57(-4.73,-4.41) |
| Lao People's Democratic Republic | 1112.61(808.29,1461.24) | 49.05(35.63,64.41) |  | 427.24(315.92,551.57) | 14.28(10.56,18.44) |  | -61.60(-72.33,-44.06) | -70.88(-79.02,-57.58) | -3.99(-4.10,-3.87) |
| Latvia | 215.51(205.51,224.98) | 28.64(27.31,29.90) |  | 12.59(11.31,13.99) | 3.25(2.92,3.61) |  | -94.16(-94.78,-93.42) | -88.64(-89.85,-87.21) | -6.44(-6.83,-6.05) |
| Lebanon | 201.60(167.86,246.66) | 15.05(12.53,18.42) |  | 73.35(62.00,88.33) | 4.40(3.72,5.30) |  | -63.62(-70.79,-55.52) | -70.76(-76.53,-64.25) | -3.76(-3.92,-3.59) |
| Lesotho | 157.59(126.73,192.12) | 18.82(15.14,22.94) |  | 168.10(133.19,209.10) | 20.04(15.88,24.93) |  | 6.67(-20.78,46.55) | 6.48(-20.92,46.29) | 1.33(0.95,1.71) |
| Liberia | 805.79(606.95,1010.72) | 58.98(44.43,73.98) |  | 494.08(285.89,738.68) | 17.71(10.25,26.48) |  | -38.68(-63.89,-1.64) | -69.98(-82.32,-51.84) | -3.76(-4.19,-3.32) |
| Libya | 332.55(266.55,402.38) | 14.41(11.55,17.43) |  | 200.18(155.25,257.67) | 9.59(7.44,12.34) |  | -39.80(-54.05,-17.67) | -33.44(-49.20,-8.97) | -0.83(-1.14,-0.52) |
| Lithuania | 258.31(245.74,270.01) | 23.32(22.19,24.38) |  | 22.25(19.82,24.60) | 4.15(3.70,4.59) |  | -91.39(-92.36,-90.37) | -82.20(-84.21,-80.09) | -5.36(-5.77,-4.94) |
| Luxembourg | 5.99(5.54,6.49) | 6.79(6.28,7.36) |  | 2.33(1.99,2.76) | 1.73(1.47,2.05) |  | -61.05(-67.94,-53.18) | -74.53(-79.03,-69.38) | -4.46(-5.14,-3.77) |
| Madagascar | 2308.85(1807.89,2825.12) | 34.33(26.88,42.00) |  | 2108.11(1554.80,2703.99) | 14.11(10.40,18.09) |  | -8.69(-33.35,26.30) | -58.90(-70.00,-43.15) | -2.41(-2.60,-2.21) |
| Malawi | 3251.46(2570.27,3955.79) | 58.34(46.12,70.98) |  | 1535.48(963.26,2266.09) | 14.50(9.10,21.40) |  | -52.78(-67.32,-30.90) | -75.15(-82.80,-63.64) | -4.38(-4.58,-4.18) |
| Malaysia | 790.61(654.56,916.50) | 9.48(7.85,10.99) |  | 442.19(384.28,504.27) | 4.31(3.75,4.92) |  | -44.07(-53.06,-30.84) | -54.50(-61.81,-43.74) | -2.56(-2.94,-2.19) |
| Maldives | 46.51(37.74,57.13) | 36.24(29.40,44.52) |  | 8.79(7.23,10.97) | 6.74(5.55,8.42) |  | -81.10(-85.38,-75.01) | -81.39(-85.60,-75.39) | -4.86(-5.75,-3.96) |
| Mali | 3040.41(2134.77,4035.67) | 61.47(43.16,81.60) |  | 3856.77(2466.44,5088.01) | 27.10(17.33,35.75) |  | 26.85(-15.81,86.86) | -55.92(-70.74,-35.07) | -2.34(-2.59,-2.10) |
| Malta | 4.69(3.93,5.21) | 4.07(3.41,4.52) |  | 2.32(1.92,2.74) | 2.76(2.28,3.27) |  | -50.58(-59.39,-39.92) | -32.16(-44.25,-17.52) | -2.05(-2.45,-1.65) |
| Marshall Islands | 2.65(2.10,3.28) | 9.88(7.83,12.23) |  | 2.55(1.87,3.34) | 11.07(8.11,14.50) |  | -3.49(-31.74,36.60) | 12.03(-20.77,58.56) | 0.13(-0.38,0.65) |
| Mauritania | 255.38(195.72,314.52) | 22.52(17.26,27.74) |  | 238.26(166.38,313.44) | 10.22(7.13,13.44) |  | -6.71(-36.48,34.36) | -54.64(-69.11,-34.67) | -2.50(-2.93,-2.08) |
| Mauritius | 42.60(40.22,45.29) | 9.89(9.34,10.51) |  | 13.88(12.26,15.24) | 4.67(4.13,5.13) |  | -67.42(-71.24,-63.44) | -52.72(-58.26,-46.94) | -2.55(-2.94,-2.17) |
| Mexico | 9426.74(8749.00,10310.78) | 21.74(20.17,23.78) |  | 2441.58(2020.36,2978.31) | 5.66(4.68,6.90) |  | -74.10(-79.12,-68.30) | -73.97(-79.01,-68.14) | -3.93(-4.23,-3.63) |
| Micronesia (Federated States of) | 8.80(6.82,11.09) | 15.30(11.86,19.29) |  | 3.78(2.91,4.73) | 9.11(7.01,11.41) |  | -57.07(-67.72,-41.22) | -40.44(-55.21,-18.45) | -1.70(-2.81,-0.58) |
| Monaco | 0.30(0.24,0.39) | 6.30(4.91,8.05) |  | 0.25(0.22,0.30) | 3.72(3.21,4.43) |  | -17.31(-38.24,10.57) | -40.83(-55.80,-20.88) | -2.70(-3.15,-2.25) |
| Mongolia | 600.59(495.63,700.68) | 52.91(43.66,61.73) |  | 178.56(146.56,216.73) | 13.60(11.16,16.51) |  | -70.27(-76.97,-60.41) | -74.29(-80.09,-65.77) | -3.67(-4.12,-3.23) |
| Montenegro | 13.09(11.20,15.12) | 6.10(5.22,7.05) |  | 3.37(2.78,3.98) | 2.24(1.85,2.65) |  | -74.27(-79.23,-67.93) | -63.30(-70.38,-54.26) | -3.22(-3.76,-2.67) |
| Morocco | 3669.20(2746.55,4611.56) | 29.40(22.01,36.95) |  | 1004.20(757.66,1303.96) | 7.78(5.87,10.10) |  | -72.63(-80.76,-61.65) | -73.56(-81.41,-62.94) | -3.94(-4.18,-3.69) |
| Mozambique | 4035.90(2984.22,5034.74) | 53.31(39.42,66.50) |  | 2920.04(1772.13,4626.14) | 16.45(9.98,26.06) |  | -27.65(-52.50,14.96) | -69.15(-79.75,-50.98) | -3.33(-3.57,-3.09) |
| Myanmar | 12949.99(9292.19,16890.99) | 67.76(48.62,88.38) |  | 4299.02(3404.45,5469.84) | 20.70(16.39,26.34) |  | -66.80(-75.22,-52.21) | -69.45(-77.20,-56.02) | -3.75(-5.31,-2.17) |
| Namibia | 131.13(106.34,162.89) | 17.22(13.96,21.39) |  | 134.09(102.14,183.71) | 12.52(9.53,17.15) |  | 2.26(-25.13,43.25) | -27.30(-46.78,1.84) | -0.38(-0.85,0.10) |
| Nauru | 0.88(0.70,1.09) | 16.89(13.34,21.01) |  | 0.80(0.60,1.08) | 15.59(11.65,21.12) |  | -9.16(-33.88,25.26) | -7.72(-32.83,27.25) | -0.32(-1.04,0.42) |
| Nepal | 4626.72(3451.59,5947.41) | 44.71(33.35,57.47) |  | 1446.72(1167.45,1804.15) | 11.62(9.37,14.49) |  | -68.73(-77.04,-55.66) | -74.02(-80.92,-63.15) | -3.89(-4.71,-3.05) |
| Netherlands | 127.57(121.84,133.50) | 3.33(3.18,3.48) |  | 43.16(38.68,47.41) | 1.17(1.05,1.28) |  | -66.16(-70.21,-62.43) | -64.86(-69.07,-60.98) | -3.21(-3.57,-2.84) |
| New Zealand | 80.52(75.94,84.92) | 7.31(6.90,7.71) |  | 48.80(43.73,53.86) | 3.74(3.35,4.13) |  | -39.40(-46.09,-32.43) | -48.81(-54.46,-42.92) | -1.16(-1.74,-0.58) |
| Nicaragua | 551.16(457.46,643.17) | 24.49(20.33,28.58) |  | 119.96(97.37,148.96) | 4.62(3.75,5.73) |  | -78.24(-83.13,-71.90) | -81.16(-85.40,-75.67) | -5.38(-6.65,-4.09) |
| Niger | 3960.47(2999.58,5102.46) | 81.63(61.83,105.17) |  | 4525.23(2652.63,6776.68) | 29.03(17.02,43.48) |  | 14.26(-29.16,76.76) | -64.43(-77.95,-44.98) | -3.45(-3.75,-3.15) |
| Nigeria | 19473.13(16078.25,22759.48) | 40.14(33.14,46.91) |  | 28710.93(15404.55,37999.55) | 22.47(12.06,29.74) |  | 47.44(-18.30,103.89) | -44.01(-68.98,-22.58) | -1.59(-1.85,-1.33) |
| Niue | 0.12(0.09,0.15) | 11.55(9.05,14.32) |  | 0.16(0.15,0.18) | 30.86(28.00,34.24) |  | 38.01(8.35,76.81) | 167.18(109.77,242.30) | 0.72(-0.69,2.14) |
| North Macedonia | 95.72(75.81,107.62) | 13.73(10.88,15.44) |  | 12.33(10.36,14.78) | 2.76(2.32,3.31) |  | -87.11(-89.71,-82.87) | -79.89(-83.94,-73.27) | -5.38(-5.90,-4.86) |
| Northern Mariana Islands | 1.10(0.86,1.40) | 6.77(5.30,8.63) |  | 0.93(0.79,1.10) | 6.21(5.24,7.31) |  | -15.19(-33.71,9.58) | -8.29(-28.32,18.50) | 0.31(-0.60,1.22) |
| Norway | 48.98(46.88,51.21) | 4.40(4.21,4.60) |  | 14.89(13.68,16.10) | 1.20(1.10,1.30) |  | -69.61(-72.36,-66.92) | -72.76(-75.23,-70.36) | -3.66(-3.95,-3.37) |
| Oman | 137.25(104.31,176.25) | 13.69(10.41,17.58) |  | 62.40(55.39,69.68) | 4.18(3.71,4.67) |  | -54.53(-65.52,-39.39) | -69.45(-76.84,-59.28) | -2.71(-3.25,-2.17) |
| Pakistan | 16636.80(13012.45,20631.93) | 27.29(21.34,33.84) |  | 18855.18(14950.32,22952.56) | 17.26(13.69,21.01) |  | 13.33(-10.82,48.62) | -36.74(-50.22,-17.05) | -0.95(-1.81,-0.08) |
| Palau | 1.48(1.14,1.85) | 24.03(18.57,29.94) |  | 0.74(0.62,0.89) | 16.78(13.99,20.21) |  | -49.85(-60.38,-35.26) | -30.18(-44.84,-9.86) | -0.84(-0.99,-0.68) |
| Palestine | 153.80(122.29,186.67) | 12.89(10.25,15.64) |  | 103.57(85.69,122.88) | 4.30(3.56,5.10) |  | -32.66(-47.11,-11.00) | -66.62(-73.78,-55.88) | -2.96(-3.25,-2.67) |
| Panama | 195.26(172.32,219.67) | 17.96(15.85,20.21) |  | 98.11(80.57,120.35) | 6.51(5.35,7.98) |  | -49.75(-59.84,-36.93) | -63.76(-71.03,-54.52) | -3.10(-3.36,-2.85) |
| Papua New Guinea | 561.01(409.41,728.54) | 26.36(19.23,34.23) |  | 1077.03(810.98,1410.01) | 21.98(16.55,28.78) |  | 91.98(47.88,152.49) | -16.58(-35.74,9.71) | -0.84(-1.61,-0.06) |
| Paraguay | 312.31(257.94,369.15) | 15.09(12.47,17.84) |  | 209.45(161.63,266.85) | 7.84(6.05,9.99) |  | -32.94(-51.16,-12.15) | -48.04(-62.16,-31.94) | -1.97(-2.22,-1.71) |
| Peru | 6211.61(4703.36,7148.97) | 58.33(44.16,67.13) |  | 1457.79(1076.25,1899.72) | 11.65(8.60,15.19) |  | -76.53(-82.83,-67.74) | -80.02(-85.38,-72.54) | -5.23(-5.64,-4.82) |
| Philippines | 8230.89(7372.85,9183.34) | 25.68(23.00,28.65) |  | 4129.55(3564.08,4718.81) | 9.22(7.96,10.54) |  | -49.83(-57.34,-40.52) | -64.09(-69.46,-57.42) | -1.99(-2.85,-1.13) |
| Poland | 1260.78(1231.70,1295.19) | 10.13(9.89,10.40) |  | 149.27(134.55,162.30) | 1.94(1.75,2.11) |  | -88.16(-89.32,-87.05) | -80.85(-82.73,-79.06) | -4.99(-5.27,-4.71) |
| Portugal | 281.21(266.29,297.04) | 9.43(8.93,9.96) |  | 34.86(30.68,38.86) | 1.82(1.60,2.03) |  | -87.60(-89.18,-86.02) | -80.72(-83.17,-78.26) | -5.61(-6.09,-5.13) |
| Puerto Rico | 83.90(79.86,88.18) | 6.30(6.00,6.62) |  | 10.02(8.32,11.83) | 1.53(1.27,1.81) |  | -88.06(-90.12,-85.72) | -75.65(-79.86,-70.88) | -3.52(-5.63,-1.37) |
| Qatar | 19.68(16.08,24.00) | 12.85(10.50,15.68) |  | 21.75(17.71,26.93) | 3.64(2.97,4.51) |  | 10.55(-14.83,42.55) | -71.66(-78.17,-63.45) | -3.24(-3.72,-2.76) |
| Republic of Korea | 2619.86(2289.87,2936.75) | 16.45(14.38,18.44) |  | 147.41(127.23,184.05) | 1.76(1.52,2.20) |  | -94.37(-95.46,-92.61) | -89.31(-91.37,-85.96) | -7.21(-7.59,-6.83) |
| Republic of Moldova | 513.76(463.87,568.62) | 32.49(29.33,35.96) |  | 58.38(48.28,70.85) | 8.39(6.93,10.18) |  | -88.64(-90.86,-85.99) | -74.19(-79.25,-68.17) | -4.29(-4.47,-4.11) |
| Romania | 1841.13(1741.33,1928.03) | 24.54(23.21,25.70) |  | 192.49(173.17,211.80) | 4.76(4.29,5.24) |  | -89.55(-90.77,-88.31) | -80.59(-82.87,-78.30) | -5.30(-5.54,-5.05) |
| Russian Federation | 10262.00(10067.51,10434.54) | 22.71(22.28,23.09) |  | 2144.05(2021.17,2234.11) | 6.35(5.98,6.61) |  | -79.11(-80.24,-78.12) | -72.06(-73.58,-70.75) | -4.49(-5.07,-3.91) |
| Rwanda | 1970.47(1543.36,2387.66) | 47.61(37.29,57.69) |  | 844.23(586.83,1144.57) | 13.11(9.11,17.78) |  | -57.16(-68.80,-37.88) | -72.46(-79.94,-60.07) | -4.70(-5.10,-4.30) |
| Saint Kitts and Nevis | 2.77(2.48,3.05) | 15.05(13.50,16.59) |  | 1.10(0.92,1.30) | 7.83(6.54,9.22) |  | -60.18(-67.37,-52.33) | -47.97(-57.38,-37.72) | -2.07(-2.55,-1.60) |
| Saint Lucia | 10.14(8.70,11.67) | 15.18(13.03,17.47) |  | 3.33(2.74,4.06) | 7.94(6.54,9.69) |  | -67.13(-74.26,-58.04) | -47.69(-59.04,-33.22) | -2.14(-2.69,-1.59) |
| Saint Vincent and the Grenadines | 10.13(8.72,11.72) | 18.84(16.22,21.80) |  | 3.22(2.72,3.84) | 9.56(8.08,11.39) |  | -68.19(-74.93,-60.10) | -49.27(-60.01,-36.37) | -1.70(-2.28,-1.11) |
| Samoa | 15.40(12.85,18.24) | 16.81(14.03,19.91) |  | 6.97(5.33,8.93) | 6.90(5.28,8.85) |  | -54.73(-64.73,-43.03) | -58.92(-68.00,-48.31) | -1.56(-3.34,0.25) |
| San Marino | 0.18(0.15,0.22) | 3.05(2.51,3.68) |  | 0.06(0.04,0.07) | 0.90(0.68,1.18) |  | -69.16(-77.47,-56.76) | -70.43(-78.39,-58.54) | -3.78(-3.93,-3.64) |
| Sao Tome and Principe | 17.69(13.65,22.67) | 25.31(19.54,32.43) |  | 9.67(7.72,12.54) | 9.50(7.58,12.32) |  | -45.34(-58.74,-27.41) | -62.48(-71.68,-50.17) | -4.51(-5.04,-3.98) |
| Saudi Arabia | 2161.82(1704.99,2740.77) | 26.33(20.76,33.38) |  | 426.27(336.94,535.52) | 4.21(3.33,5.28) |  | -80.28(-85.67,-74.11) | -84.02(-88.39,-79.02) | -5.32(-5.58,-5.06) |
| Senegal | 1885.29(1503.39,2343.59) | 42.40(33.81,52.70) |  | 1202.65(837.18,1622.97) | 14.82(10.32,20.00) |  | -36.21(-53.01,-12.08) | -65.04(-74.25,-51.82) | -3.07(-3.43,-2.71) |
| Serbia | 256.81(229.69,288.52) | 8.85(7.92,9.95) |  | 26.48(22.35,30.94) | 1.41(1.19,1.65) |  | -89.69(-91.39,-87.62) | -84.03(-86.68,-80.83) | -6.10(-6.53,-5.66) |
| Seychelles | 3.63(3.15,4.10) | 11.63(10.07,13.13) |  | 0.82(0.66,1.00) | 2.68(2.16,3.28) |  | -77.45(-82.16,-71.36) | -76.93(-81.74,-70.69) | -1.52(-2.50,-0.54) |
| Sierra Leone | 1390.41(1063.37,1730.44) | 63.04(48.21,78.45) |  | 1239.49(767.19,1707.66) | 27.24(16.86,37.53) |  | -10.85(-42.99,36.04) | -56.79(-72.37,-34.06) | -2.52(-2.91,-2.12) |
| Singapore | 30.40(28.44,32.51) | 3.30(3.09,3.53) |  | 9.43(8.27,10.59) | 0.90(0.79,1.01) |  | -68.99(-72.78,-64.62) | -72.63(-75.97,-68.77) | -3.93(-4.13,-3.72) |
| Slovakia | 147.22(134.23,158.53) | 8.39(7.65,9.03) |  | 41.18(33.97,48.04) | 3.67(3.03,4.29) |  | -72.03(-77.06,-67.20) | -56.21(-64.08,-48.65) | -2.39(-2.56,-2.23) |
| Slovenia | 34.56(32.31,36.89) | 6.21(5.80,6.63) |  | 5.62(4.85,6.41) | 1.39(1.20,1.58) |  | -83.73(-86.03,-81.16) | -77.62(-80.78,-74.09) | -4.83(-5.22,-4.44) |
| Solomon Islands | 30.98(22.04,40.19) | 15.92(11.32,20.65) |  | 39.04(30.32,48.15) | 11.81(9.17,14.56) |  | 26.02(-1.32,65.03) | -25.85(-41.94,-2.89) | -0.29(-0.95,0.37) |
| Somalia | 2222.28(1436.80,3101.56) | 46.96(30.36,65.54) |  | 3245.17(1908.44,5250.84) | 25.38(14.93,41.07) |  | 46.03(-2.33,119.98) | -45.95(-63.85,-18.57) | -1.67(-2.18,-1.16) |
| South Africa | 4041.85(3443.28,4626.77) | 22.94(19.54,26.26) |  | 2543.22(2125.04,2998.44) | 12.77(10.67,15.05) |  | -37.08(-47.61,-24.48) | -44.33(-53.65,-33.18) | -1.59(-1.84,-1.34) |
| South Sudan | 1527.12(1142.59,1947.11) | 46.47(34.77,59.25) |  | 1981.13(1434.40,2593.15) | 36.10(26.14,47.26) |  | 29.73(-4.84,85.01) | -22.31(-43.01,10.79) | -0.44(-1.01,0.12) |
| Spain | 632.56(608.96,656.50) | 5.67(5.46,5.89) |  | 137.14(125.35,148.88) | 1.56(1.42,1.69) |  | -78.32(-80.39,-76.32) | -72.56(-75.19,-70.03) | -4.08(-4.33,-3.83) |
| Sri Lanka | 1298.00(1135.90,1452.97) | 17.93(15.69,20.07) |  | 369.88(282.39,475.42) | 5.36(4.09,6.89) |  | -71.50(-78.25,-62.79) | -70.10(-77.18,-60.96) | -4.16(-6.21,-2.07) |
| Sudan | 8302.41(5827.91,11056.76) | 75.16(52.76,100.10) |  | 3529.48(2608.21,4654.88) | 16.51(12.20,21.78) |  | -57.49(-69.16,-36.37) | -78.03(-84.06,-67.12) | -4.60(-4.80,-4.40) |
| Suriname | 41.79(26.56,49.69) | 24.44(15.53,29.06) |  | 21.09(16.60,26.26) | 11.11(8.74,13.83) |  | -49.52(-62.82,-21.13) | -54.54(-66.51,-28.96) | -2.26(-2.64,-1.88) |
| Sweden | 62.27(59.08,65.40) | 2.96(2.80,3.10) |  | 23.03(20.63,25.36) | 0.95(0.85,1.05) |  | -63.02(-67.20,-58.80) | -67.74(-71.39,-64.05) | -3.13(-3.61,-2.65) |
| Switzerland | 82.98(79.24,86.71) | 5.26(5.02,5.50) |  | 25.74(23.40,28.49) | 1.46(1.33,1.62) |  | -68.98(-72.13,-65.46) | -72.25(-75.06,-69.09) | -4.05(-4.25,-3.85) |
| Syrian Arab Republic | 1197.53(949.95,1458.46) | 16.29(12.92,19.84) |  | 297.37(232.98,368.98) | 5.47(4.28,6.78) |  | -75.17(-81.73,-66.03) | -66.44(-75.31,-54.09) | -3.39(-3.85,-2.93) |
| Taiwan (Province of China) | 1302.17(1260.00,1349.33) | 17.82(17.24,18.46) |  | 156.87(141.14,171.87) | 3.88(3.49,4.25) |  | -87.95(-89.18,-86.76) | -78.23(-80.46,-76.08) | -4.99(-5.69,-4.28) |
| Tajikistan | 1103.28(926.04,1273.23) | 38.38(32.22,44.29) |  | 760.00(588.07,978.45) | 17.06(13.20,21.96) |  | -31.11(-47.30,-6.65) | -55.55(-66.00,-39.77) | -3.17(-3.65,-2.69) |
| Thailand | 5613.89(4689.78,6501.83) | 24.68(20.62,28.58) |  | 1213.02(1035.84,1403.08) | 8.89(7.59,10.28) |  | -78.39(-81.92,-73.74) | -63.99(-69.86,-56.24) | -3.45(-3.98,-2.92) |
| Timor-Leste | 188.61(141.38,247.13) | 46.38(34.77,60.77) |  | 118.45(97.72,143.38) | 17.28(14.26,20.92) |  | -37.20(-52.22,-17.42) | -62.74(-71.65,-51.01) | -3.96(-4.49,-3.42) |
| Togo | 686.11(535.08,842.67) | 31.87(24.86,39.14) |  | 659.13(380.90,926.05) | 15.79(9.12,22.18) |  | -3.93(-36.60,38.65) | -50.47(-67.31,-28.52) | -1.97(-2.22,-1.71) |
| Tokelau | 0.07(0.05,0.09) | 9.16(6.79,12.03) |  | 0.12(0.10,0.15) | 24.28(19.42,29.20) |  | 78.77(41.81,139.40) | 165.02(110.24,254.91) | -0.48(-1.92,0.98) |
| Tonga | 6.66(5.37,8.14) | 12.48(10.06,15.26) |  | 4.15(3.22,5.37) | 8.38(6.50,10.82) |  | -37.62(-53.80,-14.71) | -32.86(-50.28,-8.21) | -0.77(-1.32,-0.22) |
| Trinidad and Tobago | 75.28(67.80,83.08) | 14.49(13.05,15.99) |  | 20.77(16.45,26.24) | 5.71(4.52,7.21) |  | -72.41(-78.55,-64.55) | -60.62(-69.38,-49.41) | -2.62(-2.96,-2.28) |
| Tunisia | 907.06(734.55,1098.30) | 22.73(18.41,27.53) |  | 190.36(144.27,246.54) | 5.31(4.02,6.88) |  | -79.01(-84.50,-71.74) | -76.65(-82.75,-68.55) | -4.27(-4.39,-4.14) |
| Turkey | 5182.53(4257.87,6119.47) | 19.42(15.96,22.94) |  | 1010.99(853.46,1171.46) | 4.09(3.45,4.74) |  | -80.49(-84.61,-75.45) | -78.94(-83.38,-73.49) | -5.18(-5.99,-4.37) |
| Turkmenistan | 802.81(697.93,904.81) | 42.58(37.02,47.99) |  | 192.70(162.93,229.12) | 9.83(8.31,11.69) |  | -76.00(-80.40,-70.56) | -76.92(-81.15,-71.69) | -5.31(-5.80,-4.82) |
| Tuvalu | 1.05(0.78,1.32) | 24.47(18.32,30.92) |  | 0.44(0.34,0.56) | 8.94(6.98,11.40) |  | -58.00(-69.03,-39.94) | -63.47(-73.06,-47.76) | -3.31(-4.80,-1.79) |
| Uganda | 3194.12(2398.90,4035.28) | 31.02(23.30,39.19) |  | 3519.98(2200.19,5048.16) | 14.11(8.82,20.24) |  | 10.20(-22.63,63.50) | -54.50(-68.06,-32.50) | -2.32(-2.51,-2.12) |
| Ukraine | 3160.96(2844.13,3510.68) | 21.00(18.90,23.32) |  | 495.38(424.61,567.62) | 5.87(5.03,6.72) |  | -84.33(-86.86,-81.38) | -72.07(-76.57,-66.81) | -4.67(-5.05,-4.29) |
| United Arab Emirates | 107.57(87.88,132.55) | 15.21(12.43,18.75) |  | 53.74(44.92,63.88) | 3.17(2.65,3.77) |  | -50.04(-59.37,-38.75) | -79.16(-83.05,-74.45) | -4.31(-5.04,-3.57) |
| United Kingdom | 586.50(573.31,600.12) | 3.95(3.86,4.05) |  | 180.67(170.20,190.76) | 1.15(1.09,1.22) |  | -69.20(-70.98,-67.50) | -70.81(-72.50,-69.21) | -3.29(-3.44,-3.14) |
| United Republic of Tanzania | 5824.32(4664.50,6873.83) | 39.03(31.26,46.06) |  | 4557.03(3086.40,6780.85) | 14.77(10.00,21.97) |  | -21.76(-43.97,12.05) | -62.16(-72.90,-45.81) | -2.60(-2.79,-2.41) |
| United States of America | 5729.47(5643.96,5814.40) | 7.74(7.62,7.85) |  | 3303.12(3024.10,3608.39) | 4.06(3.72,4.44) |  | -42.35(-47.33,-37.24) | -47.50(-52.04,-42.85) | -1.72(-1.93,-1.50) |
| United States Virgin Islands | 5.44(4.55,6.34) | 13.05(10.91,15.20) |  | 0.91(0.66,1.23) | 4.98(3.64,6.74) |  | -83.32(-88.28,-76.68) | -61.81(-73.17,-46.60) | -2.58(-3.27,-1.89) |
| Uruguay | 284.71(270.51,298.62) | 26.43(25.12,27.73) |  | 52.39(46.31,58.57) | 5.80(5.13,6.48) |  | -81.60(-83.90,-79.28) | -78.06(-80.81,-75.30) | -5.17(-5.48,-4.85) |
| Uzbekistan | 3773.50(3317.97,4262.06) | 35.35(31.08,39.92) |  | 1726.48(1453.50,2073.32) | 13.69(11.53,16.44) |  | -54.25(-62.71,-43.92) | -61.27(-68.43,-52.52) | -3.24(-3.56,-2.93) |
| Vanuatu | 9.72(7.23,12.45) | 11.64(8.66,14.92) |  | 14.31(11.14,17.76) | 9.71(7.56,12.05) |  | 47.26(17.04,94.55) | -16.65(-33.76,10.11) | -0.67(-1.61,0.27) |
| Venezuela (Bolivarian Republic of) | 1771.69(1694.29,1845.65) | 19.52(18.67,20.34) |  | 1172.28(896.35,1506.26) | 13.39(10.24,17.21) |  | -33.83(-49.58,-15.39) | -31.38(-47.71,-12.26) | -1.34(-3.07,0.42) |
| Viet Nam | 13740.98(11150.89,16390.37) | 40.81(33.12,48.68) |  | 4906.07(3900.89,6073.00) | 15.52(12.34,19.21) |  | -64.30(-72.13,-54.44) | -61.98(-70.32,-51.49) | -2.78(-3.00,-2.56) |
| Yemen | 3696.63(2642.38,4929.88) | 43.97(31.43,58.63) |  | 2374.15(1705.23,3130.42) | 13.59(9.76,17.92) |  | -35.78(-54.26,-5.05) | -69.09(-77.99,-54.31) | -3.74(-3.89,-3.60) |
| Zambia | 2048.24(1658.68,2475.49) | 43.58(35.29,52.66) |  | 1365.73(920.20,1945.94) | 13.06(8.80,18.61) |  | -33.32(-54.68,-3.63) | -70.02(-79.62,-56.67) | -3.68(-3.97,-3.39) |
| Zimbabwe | 828.84(594.73,1041.91) | 13.76(9.88,17.30) |  | 1485.82(1165.14,1854.87) | 18.61(14.59,23.23) |  | 79.26(35.94,140.63) | 35.22(2.54,81.51) | 2.41(1.79,3.03) |
