# Supplementary material for: Global patterns and health impact of unintentional injuries among children and adolescents, 1990–2021
Source: Front Public Health. 2025 Sep 24;13:1626739. doi: 10.3389/fpubh.2025.1626739 (PMC12504300; doi:10.3389/fpubh.2025.1626739)
Supplement: Supplementary file 5 [file Table_4.DOCX]

Table S4. DALYs of Unintentional Injuries at the National Level

| location | 1990 | |  | 2021 | |  | 1990-2021 | | |
| --- | --- | --- | --- | --- | --- | --- | --- | --- | --- |
|  | DALYs cases | DALYs rate |  | DALYs cases | DALYs rate |  | Cases change | Rate Change | EAPC |
| Afghanistan | 314552.20(221088.50,423467.22) | 5644.26(3967.16,7598.60) |  | 456669.08(342207.89,611167.43) | 2568.92(1925.03,3438.02) |  | 45.18(3.67,106.59) | -54.49(-67.50,-35.23) | -2.87(-3.42,-2.32) |
| Albania | 28598.91(24721.22,32842.32) | 1977.91(1709.73,2271.39) |  | 4574.36(3771.22,5687.16) | 741.24(611.10,921.56) |  | -84.01(-86.59,-81.05) | -62.52(-68.57,-55.61) | -3.33(-3.55,-3.11) |
| Algeria | 280460.65(231985.32,332064.37) | 2074.94(1716.30,2456.72) |  | 101392.75(84023.09,123358.87) | 616.41(510.81,749.96) |  | -63.85(-70.31,-56.91) | -70.29(-75.61,-64.59) | -3.60(-3.93,-3.28) |
| American Samoa | 251.21(214.98,288.88) | 1049.91(898.48,1207.35) |  | 160.94(134.12,193.14) | 841.37(701.13,1009.69) |  | -35.93(-48.55,-19.93) | -19.86(-35.64,0.16) | -0.30(-1.74,1.15) |
| Andorra | 47.82(35.58,64.35) | 355.30(264.35,478.15) |  | 40.54(28.51,57.62) | 281.94(198.29,400.73) |  | -15.23(-26.58,-6.13) | -20.65(-31.28,-12.13) | -0.74(-0.81,-0.67) |
| Angola | 418811.41(325246.19,534469.58) | 7263.44(5640.74,9269.30) |  | 307271.54(209025.38,428425.93) | 1645.30(1119.24,2294.03) |  | -26.63(-52.78,17.63) | -77.35(-85.42,-63.68) | -4.67(-5.03,-4.30) |
| Antigua and Barbuda | 301.80(261.71,346.65) | 1263.29(1095.50,1451.01) |  | 206.57(186.15,232.99) | 884.13(796.73,997.20) |  | -31.56(-40.45,-21.49) | -30.01(-39.11,-19.72) | -1.81(-2.79,-0.82) |
| Argentina | 259967.05(244870.49,278931.15) | 1995.63(1879.74,2141.21) |  | 92985.28(80646.08,110443.40) | 678.16(588.16,805.48) |  | -64.23(-67.91,-59.64) | -66.02(-69.51,-61.66) | -3.33(-3.48,-3.17) |
| Armenia | 41356.69(37191.38,46257.36) | 3114.63(2800.94,3483.71) |  | 5512.04(4634.00,6702.17) | 722.30(607.24,878.26) |  | -86.67(-89.00,-83.68) | -76.81(-80.87,-71.60) | -4.73(-5.03,-4.43) |
| Australia | 44991.07(37751.02,54956.62) | 869.89(729.90,1062.57) |  | 31558.28(23996.88,42201.84) | 505.88(384.67,676.50) |  | -29.86(-37.48,-22.84) | -41.85(-48.16,-36.03) | -1.48(-1.62,-1.33) |
| Austria | 12932.82(11201.14,15380.00) | 690.41(597.97,821.06) |  | 6379.91(4980.42,8332.94) | 363.55(283.80,474.84) |  | -50.67(-56.31,-45.37) | -47.34(-53.37,-41.69) | -1.76(-1.92,-1.60) |
| Azerbaijan | 85726.52(72929.67,97649.19) | 2738.78(2329.95,3119.68) |  | 30289.96(25523.54,36464.25) | 994.16(837.72,1196.81) |  | -64.67(-71.99,-55.57) | -63.70(-71.22,-54.36) | -3.40(-3.56,-3.24) |
| Bahamas | 1890.19(1649.98,2149.33) | 1755.70(1532.58,1996.39) |  | 923.76(769.02,1129.55) | 801.41(667.16,979.95) |  | -51.13(-60.04,-40.81) | -54.35(-62.68,-44.72) | -1.10(-2.72,0.54) |
| Bahrain | 1675.32(1511.07,1850.54) | 837.26(755.17,924.83) |  | 1270.93(1090.20,1460.73) | 314.96(270.18,362.00) |  | -24.14(-34.08,-12.80) | -62.38(-67.31,-56.76) | -2.98(-3.15,-2.80) |
| Bangladesh | 4386192.23(3633957.58,5165350.46) | 7266.81(6020.55,8557.67) |  | 824234.69(674283.99,1002577.38) | 1351.45(1105.58,1643.86) |  | -81.21(-85.40,-75.67) | -81.40(-85.55,-75.92) | -5.76(-6.42,-5.08) |
| Barbados | 860.20(767.58,963.35) | 1013.14(904.06,1134.63) |  | 343.31(273.84,434.51) | 517.19(412.53,654.58) |  | -60.09(-67.97,-50.20) | -48.95(-59.03,-36.30) | -1.85(-2.12,-1.58) |
| Belarus | 64955.31(58397.59,72395.74) | 2069.95(1860.97,2307.06) |  | 13091.72(11076.39,15578.19) | 646.42(546.91,769.19) |  | -79.85(-82.67,-76.77) | -68.77(-73.14,-64.01) | -4.14(-4.57,-3.70) |
| Belgium | 19359.60(17046.68,22450.06) | 783.25(689.67,908.28) |  | 11235.52(9093.37,14201.38) | 441.60(357.41,558.18) |  | -41.96(-47.82,-36.05) | -43.62(-49.30,-37.88) | -1.42(-1.77,-1.08) |
| Belize | 3204.91(2902.51,3562.25) | 3124.41(2829.61,3472.78) |  | 1527.12(1346.37,1746.26) | 903.36(796.44,1032.99) |  | -52.35(-59.74,-44.31) | -71.09(-75.57,-66.21) | -3.71(-4.09,-3.33) |
| Benin | 124454.64(92089.16,153698.88) | 4336.85(3209.02,5355.92) |  | 175396.89(103984.79,239623.03) | 2333.55(1383.46,3188.04) |  | 40.93(-16.82,116.84) | -46.19(-68.24,-17.21) | -1.70(-1.90,-1.49) |
| Bermuda | 129.65(112.57,147.98) | 815.35(707.95,930.60) |  | 48.51(39.96,58.62) | 421.93(347.56,509.87) |  | -62.58(-68.44,-55.24) | -48.25(-56.36,-38.09) | -2.03(-2.54,-1.51) |
| Bhutan | 8270.26(4218.05,10766.41) | 2428.05(1238.37,3160.88) |  | 2831.07(2234.18,3620.56) | 1110.33(876.23,1419.97) |  | -65.77(-75.73,-34.92) | -54.27(-67.57,-13.06) | -3.32(-4.02,-2.61) |
| Bolivia (Plurinational State of) | 189035.08(155118.37,230219.41) | 5644.76(4631.98,6874.56) |  | 73252.83(60799.66,90390.56) | 1611.09(1337.20,1988.01) |  | -61.25(-68.82,-51.13) | -71.46(-77.04,-64.01) | -4.02(-4.09,-3.95) |
| Bosnia and Herzegovina | 15680.98(12695.12,18807.40) | 1058.27(856.76,1269.26) |  | 2905.97(2194.10,3803.46) | 436.94(329.90,571.88) |  | -81.47(-84.85,-77.49) | -58.71(-66.24,-49.86) | -2.59(-2.86,-2.33) |
| Botswana | 12209.96(9860.65,14842.75) | 1648.94(1331.67,2004.50) |  | 12150.97(9499.05,15504.20) | 1325.07(1035.88,1690.74) |  | -0.48(-21.73,25.70) | -19.64(-36.79,1.50) | -0.16(-0.45,0.14) |
| Brazil | 1082984.87(997330.83,1183574.58) | 1611.76(1484.29,1761.47) |  | 396943.18(346403.04,452949.02) | 621.03(541.96,708.65) |  | -63.35(-67.57,-58.39) | -61.47(-65.91,-56.25) | -2.73(-2.99,-2.48) |
| Brunei Darussalam | 1186.83(1041.45,1328.34) | 1031.79(905.41,1154.82) |  | 666.55(568.96,808.65) | 516.15(440.58,626.19) |  | -43.84(-51.99,-32.55) | -49.98(-57.23,-39.92) | -1.74(-1.96,-1.52) |
| Bulgaria | 41383.62(37488.90,46106.36) | 1751.10(1586.30,1950.94) |  | 8581.31(7142.65,10486.40) | 663.01(551.86,810.21) |  | -79.26(-81.51,-76.75) | -62.14(-66.25,-57.54) | -3.41(-3.61,-3.22) |
| Burkina Faso | 277407.23(209251.67,343906.15) | 4903.60(3698.84,6079.07) |  | 355235.88(223106.39,522141.87) | 2774.02(1742.23,4077.38) |  | 28.06(-13.91,89.73) | -43.43(-61.97,-16.18) | -1.43(-1.61,-1.25) |
| Burundi | 131947.81(99654.63,161832.78) | 4182.01(3158.50,5129.20) |  | 105236.15(65727.89,164305.92) | 1446.64(903.53,2258.65) |  | -20.24(-44.34,21.88) | -65.41(-75.86,-47.14) | -2.72(-3.08,-2.37) |
| Cabo Verde | 2723.92(1992.38,3489.87) | 1400.68(1024.51,1794.54) |  | 1883.16(1559.42,2207.19) | 977.02(809.05,1145.13) |  | -30.87(-49.85,-1.23) | -30.25(-49.40,-0.35) | -2.04(-2.65,-1.42) |
| Cambodia | 220302.70(173520.42,271092.08) | 3869.43(3047.74,4761.50) |  | 88525.70(70425.92,108473.04) | 1335.01(1062.05,1635.82) |  | -59.82(-69.46,-45.81) | -65.50(-73.78,-53.47) | -3.69(-3.89,-3.50) |
| Cameroon | 167206.95(122857.24,204341.11) | 2816.32(2069.33,3441.79) |  | 274718.60(163674.47,363608.76) | 1625.29(968.33,2151.18) |  | 64.30(7.09,124.51) | -42.29(-62.39,-21.14) | -1.30(-1.62,-0.99) |
| Canada | 45555.76(42364.08,49620.51) | 594.36(552.72,647.39) |  | 22277.79(19498.38,25969.60) | 270.73(236.95,315.59) |  | -51.10(-55.32,-46.41) | -54.45(-58.38,-50.08) | -2.11(-2.26,-1.96) |
| Central African Republic | 93948.00(72798.47,115425.16) | 6275.15(4862.50,7709.70) |  | 96363.36(70096.69,130417.30) | 3329.76(2422.13,4506.46) |  | 2.57(-28.23,45.73) | -46.94(-62.87,-24.61) | -1.71(-1.88,-1.54) |
| Chad | 140469.24(99986.34,183406.62) | 3984.62(2836.26,5202.61) |  | 352386.26(242200.94,457174.37) | 3218.27(2211.97,4175.27) |  | 150.86(71.41,250.10) | -19.23(-44.81,12.72) | -0.52(-0.74,-0.30) |
| Chile | 115319.04(108509.94,123267.95) | 2186.33(2057.24,2337.03) |  | 28851.13(23795.12,35818.83) | 589.55(486.24,731.93) |  | -74.98(-78.74,-70.22) | -73.03(-77.09,-67.90) | -3.75(-4.28,-3.22) |
| China | 19598018.69(17123768.61,22512267.03) | 4403.64(3847.68,5058.46) |  | 2704449.09(2353559.85,3094067.99) | 808.98(704.02,925.53) |  | -86.20(-88.53,-83.62) | -81.63(-84.73,-78.19) | -5.31(-5.56,-5.06) |
| Colombia | 285294.81(255223.47,318457.67) | 1895.29(1695.52,2115.60) |  | 88854.47(74288.31,106590.24) | 610.29(510.24,732.11) |  | -68.86(-73.12,-64.39) | -67.80(-72.21,-63.18) | -3.14(-3.41,-2.86) |
| Comoros | 7701.10(5419.51,10021.29) | 2913.58(2050.38,3791.38) |  | 4203.33(3343.15,5268.25) | 1338.86(1064.87,1678.06) |  | -45.42(-59.36,-21.19) | -54.05(-65.79,-33.65) | -2.52(-2.77,-2.27) |
| Congo | 40674.79(32599.37,50635.68) | 3065.90(2457.21,3816.71) |  | 27088.72(19445.06,37114.28) | 1089.28(781.92,1492.43) |  | -33.40(-50.35,-7.64) | -64.47(-73.51,-50.73) | -3.46(-3.85,-3.08) |
| Cook Islands | 98.71(87.46,111.26) | 1146.39(1015.78,1292.20) |  | 14.69(12.46,17.28) | 283.39(240.36,333.38) |  | -85.12(-87.63,-82.21) | -75.28(-79.45,-70.45) | -4.84(-6.74,-2.90) |
| Costa Rica | 16957.19(15468.23,18703.18) | 1195.73(1090.73,1318.84) |  | 7459.58(6322.84,8997.52) | 544.90(461.87,657.25) |  | -56.01(-60.74,-51.60) | -54.43(-59.33,-49.86) | -2.52(-2.68,-2.35) |
| Croatia | 201301.79(156900.67,247108.56) | 2909.62(2267.84,3571.71) |  | 245421.45(157341.72,338896.10) | 1706.14(1093.82,2355.96) |  | -75.20(-78.67,-72.07) | -59.17(-64.89,-54.02) | -1.24(-1.49,-0.99) |
| Cuba | 13871.79(12270.07,15847.62) | 1045.79(925.04,1194.75) |  | 3440.07(2715.31,4404.36) | 426.95(337.00,546.63) |  | -69.29(-72.65,-65.58) | -53.30(-58.40,-47.65) | -2.99(-3.15,-2.84) |
| Cyprus | 42619.16(39801.82,45750.73) | 1167.00(1089.86,1252.75) |  | 13086.72(11310.99,15496.35) | 545.01(471.06,645.36) |  | -55.17(-63.00,-47.51) | -59.26(-66.38,-52.30) | -2.27(-2.55,-1.99) |
| Czechia | 2295.60(1997.84,2672.10) | 886.12(771.18,1031.45) |  | 1029.14(819.44,1299.69) | 360.99(287.44,455.89) |  | -73.01(-76.30,-69.64) | -63.00(-67.51,-58.38) | -2.65(-2.88,-2.43) |
| C么te d'Ivoire | 38191.26(33534.61,44441.80) | 1254.69(1101.70,1460.03) |  | 10309.59(8061.45,13253.36) | 464.29(363.04,596.86) |  | 21.92(-14.59,63.50) | -41.36(-58.92,-21.36) | -3.06(-3.27,-2.85) |
| Democratic People's Republic of Korea | 165468.38(120595.57,220519.40) | 2124.29(1548.21,2831.04) |  | 62968.68(49047.94,82522.55) | 953.74(742.89,1249.91) |  | -61.95(-72.27,-45.84) | -55.10(-67.28,-36.11) | -2.21(-2.52,-1.89) |
| Democratic Republic of the Congo | 1045821.46(801438.90,1303295.39) | 4830.54(3701.77,6019.79) |  | 698631.73(464856.39,1022925.88) | 1458.31(970.33,2135.23) |  | -33.20(-50.75,-6.50) | -69.81(-77.74,-57.75) | -3.30(-3.65,-2.95) |
| Denmark | 6800.56(5778.79,8198.65) | 543.92(462.20,655.75) |  | 4272.03(3225.30,5706.89) | 330.09(249.21,440.96) |  | -37.18(-44.95,-29.62) | -39.31(-46.82,-32.01) | -1.47(-1.58,-1.35) |
| Djibouti | 5386.67(4207.79,6820.96) | 2401.83(1876.19,3041.36) |  | 6132.99(4408.87,8588.19) | 1160.73(834.43,1625.41) |  | 13.85(-17.84,58.07) | -51.67(-65.13,-32.90) | -2.46(-3.17,-1.75) |
| Dominica | 451.42(379.79,525.64) | 1377.43(1158.88,1603.91) |  | 229.41(194.98,270.00) | 1180.96(1003.69,1389.91) |  | -49.18(-59.10,-35.77) | -14.26(-31.00,8.36) | 0.95(-0.65,2.58) |
| Dominican Republic | 90874.87(76928.08,103834.72) | 2594.84(2196.60,2964.89) |  | 36803.63(30139.55,43737.19) | 945.94(774.66,1124.15) |  | -59.50(-67.57,-48.86) | -63.55(-70.81,-53.97) | -3.02(-3.41,-2.63) |
| Ecuador | 112100.62(104596.79,120174.04) | 2261.22(2109.86,2424.07) |  | 59659.70(49810.96,71009.86) | 901.71(752.86,1073.26) |  | -46.78(-55.48,-36.61) | -60.12(-66.64,-52.50) | -2.89(-3.16,-2.61) |
| Egypt | 609545.32(510334.24,705665.64) | 2182.97(1827.66,2527.21) |  | 274726.95(226077.44,328948.39) | 590.17(485.66,706.65) |  | -54.93(-64.18,-43.35) | -72.96(-78.51,-66.02) | -3.49(-3.76,-3.22) |
| El Salvador | 65026.83(54792.74,75025.43) | 2359.98(1988.56,2722.86) |  | 17027.11(14239.42,20795.87) | 714.80(597.77,873.01) |  | -73.82(-78.40,-67.37) | -69.71(-75.02,-62.26) | -3.75(-4.39,-3.11) |
| Equatorial Guinea | 12152.57(9336.63,15479.56) | 5063.99(3890.58,6450.34) |  | 9109.04(5768.46,13760.60) | 1182.79(749.02,1786.79) |  | -25.04(-51.81,20.07) | -76.64(-84.98,-62.58) | -6.17(-6.61,-5.72) |
| Eritrea | 69716.65(52434.85,90129.40) | 3544.69(2666.01,4582.57) |  | 58974.02(42652.13,81696.46) | 1833.40(1325.98,2539.80) |  | -15.41(-43.89,34.89) | -48.28(-65.69,-17.52) | -1.96(-2.12,-1.79) |
| Estonia | 11941.73(11241.51,12836.33) | 2599.86(2447.41,2794.62) |  | 1325.76(1108.85,1568.05) | 472.97(395.59,559.42) |  | -88.90(-90.48,-87.38) | -81.81(-84.41,-79.32) | -5.77(-6.00,-5.54) |
| Eswatini | 9899.53(7748.66,12020.76) | 2074.82(1624.02,2519.40) |  | 8161.60(6290.46,10083.12) | 1525.51(1175.77,1884.67) |  | -17.56(-36.81,10.59) | -26.47(-43.64,-1.38) | -0.46(-0.65,-0.27) |
| Ethiopia | 1467121.13(1085375.92,1866854.33) | 4978.82(3683.33,6335.36) |  | 824759.40(615623.25,1180605.70) | 1441.94(1076.30,2064.07) |  | -43.78(-60.49,-14.12) | -71.04(-79.65,-55.75) | -4.20(-4.33,-4.08) |
| Fiji | 4318.41(3523.86,5268.48) | 1203.89(982.38,1468.75) |  | 4193.42(3297.95,5289.06) | 1195.97(940.58,1508.45) |  | -2.89(-29.07,32.47) | -0.66(-27.44,35.52) | -0.12(-0.56,0.32) |
| Finland | 10280.07(8888.76,12347.22) | 810.24(700.58,973.16) |  | 5963.86(4685.90,7726.23) | 519.27(408.00,672.71) |  | -41.99(-48.33,-36.50) | -35.91(-42.92,-29.85) | -1.40(-1.75,-1.06) |
| France | 138361.53(124416.16,157051.81) | 857.16(770.76,972.94) |  | 70250.60(57510.51,87307.09) | 445.99(365.11,554.27) |  | -49.23(-54.21,-43.94) | -47.97(-53.08,-42.55) | -1.85(-2.06,-1.64) |
| Gabon | 10925.67(8421.19,13808.93) | 2149.96(1657.12,2717.32) |  | 7989.91(5083.96,11951.90) | 966.07(614.71,1445.13) |  | -26.87(-50.19,4.91) | -55.07(-69.39,-35.54) | -1.96(-2.23,-1.68) |
| Gambia | 16484.75(12204.59,20932.54) | 2934.32(2172.44,3726.03) |  | 17702.10(12928.21,24250.50) | 1397.59(1020.69,1914.59) |  | 7.38(-18.03,44.61) | -52.37(-63.64,-35.86) | -2.66(-2.99,-2.33) |
| Georgia | 40764.19(36741.61,45234.81) | 2268.69(2044.82,2517.49) |  | 6935.48(5880.06,8155.18) | 746.32(632.74,877.56) |  | -82.99(-85.47,-79.98) | -67.10(-71.90,-61.28) | -3.32(-3.77,-2.86) |
| Germany | 115949.36(102258.90,135425.88) | 669.05(590.05,781.43) |  | 52905.43(41390.45,69705.50) | 331.76(259.55,437.11) |  | -54.37(-60.40,-48.45) | -50.41(-56.97,-43.97) | -1.88(-2.07,-1.69) |
| Ghana | 166164.09(133343.36,203534.65) | 2011.77(1614.41,2464.22) |  | 166151.81(110096.27,238127.38) | 1018.46(674.86,1459.65) |  | -0.01(-31.11,41.65) | -49.37(-65.12,-28.28) | -1.55(-1.79,-1.32) |
| Greece | 18047.45(15633.17,21415.08) | 642.13(556.23,761.95) |  | 7306.57(5958.40,9198.67) | 383.49(312.73,482.79) |  | -59.51(-62.77,-56.17) | -40.28(-45.09,-35.34) | -1.47(-1.59,-1.35) |
| Greenland | 493.63(419.49,578.80) | 2758.95(2344.60,3235.00) |  | 141.29(117.40,167.05) | 919.22(763.80,1086.82) |  | -71.38(-76.86,-64.45) | -66.68(-73.07,-58.62) | -3.66(-4.00,-3.32) |
| Grenada | 878.98(755.97,1025.16) | 2084.92(1793.12,2431.65) |  | 277.23(248.38,313.72) | 920.89(825.07,1042.09) |  | -68.46(-73.09,-63.43) | -55.83(-62.31,-48.79) | -2.07(-2.62,-1.52) |
| Guam | 338.66(296.69,385.29) | 625.47(547.96,711.60) |  | 187.82(155.35,226.20) | 387.38(320.40,466.54) |  | -44.54(-55.13,-32.37) | -38.07(-49.90,-24.47) | -1.01(-1.39,-0.62) |
| Guatemala | 168099.21(154375.31,182916.71) | 3423.07(3143.61,3724.81) |  | 74738.84(63619.50,87890.18) | 1129.85(961.75,1328.66) |  | -55.54(-61.88,-48.68) | -66.99(-71.70,-61.90) | -2.96(-3.41,-2.52) |
| Guinea | 181115.70(131528.98,230113.39) | 5509.75(4001.27,7000.32) |  | 179342.21(115185.68,242809.09) | 2402.62(1543.12,3252.87) |  | -0.98(-37.74,48.40) | -56.39(-72.58,-34.65) | -2.07(-2.31,-1.82) |
| Guinea-Bissau | 29960.27(21990.19,39772.15) | 5094.45(3739.22,6762.87) |  | 20500.69(13897.19,28141.42) | 1832.83(1242.46,2515.94) |  | -31.57(-51.36,1.12) | -64.02(-74.43,-46.83) | -2.90(-3.32,-2.48) |
| Guyana | 10054.92(8577.89,11507.59) | 2635.03(2247.95,3015.72) |  | 3678.35(2983.96,4430.46) | 1310.36(1062.99,1578.29) |  | -63.42(-71.28,-53.39) | -50.27(-60.95,-36.64) | -1.12(-1.52,-0.72) |
| Haiti | 291692.74(218780.29,357681.56) | 8716.55(6537.73,10688.47) |  | 339306.36(281380.29,401666.36) | 6050.67(5017.71,7162.70) |  | 16.32(-5.36,44.77) | -30.58(-43.53,-13.61) | -1.00(-3.67,1.75) |
| Honduras | 78351.50(66940.64,92705.81) | 2887.37(2466.86,3416.35) |  | 39332.65(31121.45,52523.86) | 904.10(715.36,1207.31) |  | -49.80(-59.35,-36.12) | -68.69(-74.65,-60.16) | -4.39(-6.00,-2.75) |
| Hungary | 32063.25(28572.67,36556.03) | 1104.78(984.51,1259.59) |  | 8788.97(7068.34,11196.38) | 468.65(376.90,597.02) |  | -72.59(-76.09,-68.73) | -57.58(-62.99,-51.61) | -2.58(-2.69,-2.47) |
| Iceland | 508.21(434.42,602.51) | 601.34(514.03,712.93) |  | 311.00(240.97,408.57) | 349.03(270.43,458.53) |  | -38.80(-46.15,-31.27) | -41.96(-48.93,-34.82) | -2.09(-3.15,-1.03) |
| India | 11714194.66(9569124.99,13950903.62) | 2854.22(2331.57,3399.21) |  | 4713254.42(3642013.51,5937671.10) | 941.57(727.57,1186.18) |  | -59.76(-68.43,-47.11) | -67.01(-74.12,-56.63) | -3.48(-3.73,-3.22) |
| Indonesia | 1793793.90(1488564.82,2081529.08) | 2050.91(1701.93,2379.89) |  | 782574.17(634584.15,942527.47) | 867.43(703.39,1044.72) |  | -56.37(-65.52,-45.70) | -57.71(-66.57,-47.36) | -2.58(-3.84,-1.31) |
| Iran (Islamic Republic of) | 3038411.11(2808598.15,3294226.84) | 9642.83(8913.48,10454.69) |  | 131080.83(118622.04,147529.36) | 506.99(458.80,570.61) |  | -95.69(-96.20,-95.07) | -94.74(-95.37,-93.99) | -5.60(-6.70,-4.50) |
| Iraq | 335331.75(276288.47,399748.67) | 3256.11(2682.79,3881.61) |  | 184738.93(156050.07,220898.58) | 1047.46(884.79,1252.48) |  | -44.91(-55.62,-29.68) | -67.83(-74.08,-58.94) | -3.67(-3.94,-3.41) |
| Ireland | 8547.92(7334.97,10274.52) | 643.27(551.99,773.21) |  | 4499.37(3449.80,6056.49) | 340.45(261.03,458.27) |  | -47.36(-54.19,-40.62) | -47.08(-53.94,-40.29) | -1.98(-2.17,-1.78) |
| Israel | 15251.70(13598.62,17388.99) | 760.19(677.79,866.71) |  | 11201.77(8908.58,14440.94) | 332.06(264.08,428.08) |  | -26.55(-35.54,-16.54) | -56.32(-61.66,-50.36) | -2.16(-2.35,-1.97) |
| Italy | 85619.08(71942.46,105018.05) | 628.64(528.22,771.08) |  | 37188.36(27985.12,50524.88) | 354.86(267.04,482.13) |  | -56.57(-61.40,-51.95) | -43.55(-49.84,-37.55) | -1.93(-2.17,-1.69) |
| Jamaica | 9810.45(8783.10,11082.70) | 897.97(803.94,1014.43) |  | 3258.65(2684.34,4003.54) | 399.65(329.22,491.01) |  | -66.78(-71.16,-61.76) | -55.49(-61.35,-48.76) | -2.53(-2.81,-2.24) |
| Japan | 194554.90(171974.49,226108.91) | 583.13(515.46,677.71) |  | 63833.60(53458.84,78193.55) | 300.63(251.77,368.26) |  | -67.19(-69.59,-64.92) | -48.45(-52.22,-44.88) | -2.16(-2.92,-1.38) |
| Jordan | 27051.86(24153.83,30495.31) | 1290.25(1152.03,1454.49) |  | 23816.16(20514.79,28125.04) | 483.57(416.54,571.06) |  | -11.96(-26.57,5.10) | -62.52(-68.74,-55.26) | -3.42(-3.67,-3.17) |
| Kazakhstan | 210485.18(190476.39,233745.20) | 3168.48(2867.28,3518.61) |  | 72413.87(62940.21,85190.01) | 1076.93(936.04,1266.94) |  | -65.60(-70.42,-59.94) | -66.01(-70.78,-60.42) | -3.35(-3.69,-3.01) |
| Kenya | 229443.66(192235.73,273304.81) | 1663.62(1393.84,1981.64) |  | 182199.99(146675.13,232793.17) | 740.64(596.23,946.30) |  | -20.59(-35.52,-1.05) | -55.48(-63.85,-44.53) | -1.64(-1.97,-1.31) |
| Kiribati | 363.55(287.14,446.56) | 991.87(783.40,1218.34) |  | 294.07(223.48,368.46) | 550.23(418.15,689.42) |  | -19.11(-38.07,5.14) | -44.53(-57.53,-27.89) | -1.74(-1.86,-1.62) |
| Kuwait | 6425.57(5882.10,7028.88) | 936.73(857.50,1024.68) |  | 3829.53(3256.76,4541.60) | 349.58(297.29,414.58) |  | -40.40(-49.05,-30.68) | -62.68(-68.10,-56.60) | -2.81(-3.10,-2.53) |
| Kyrgyzstan | 66083.42(59565.95,73198.77) | 3129.86(2821.18,3466.86) |  | 24296.21(21820.22,27046.29) | 860.70(772.99,958.13) |  | -63.23(-67.34,-58.36) | -72.50(-75.57,-68.86) | -4.26(-4.39,-4.13) |
| Lao People's Democratic Republic | 96327.36(70887.39,126568.21) | 4246.34(3124.88,5579.43) |  | 37363.20(28213.69,47479.61) | 1248.97(943.12,1587.14) |  | -61.21(-71.59,-44.07) | -70.59(-78.45,-57.59) | -3.96(-4.08,-3.85) |
| Latvia | 20156.03(19020.24,21463.01) | 2678.84(2527.88,2852.54) |  | 1787.51(1528.62,2104.70) | 461.79(394.91,543.73) |  | -91.13(-92.24,-89.85) | -82.76(-84.92,-80.27) | -5.53(-5.81,-5.25) |
| Lebanon | 17852.10(15042.50,21418.36) | 1333.11(1123.30,1599.42) |  | 7375.95(6349.06,8730.65) | 442.67(381.04,523.97) |  | -58.68(-66.09,-50.10) | -66.79(-72.74,-59.90) | -3.40(-3.55,-3.25) |
| Lesotho | 13984.95(11385.03,16950.70) | 1670.20(1359.70,2024.40) |  | 14499.95(11736.71,17984.54) | 1728.61(1399.19,2144.02) |  | 3.68(-21.39,38.67) | 3.50(-21.53,38.42) | 1.17(0.81,1.52) |
| Liberia | 71625.03(53957.78,89320.44) | 5242.78(3949.58,6538.04) |  | 44062.68(26695.01,64557.02) | 1579.29(956.80,2313.84) |  | -38.48(-62.59,-2.13) | -69.88(-81.68,-52.08) | -3.76(-4.18,-3.33) |
| Libya | 30353.88(24743.84,36301.45) | 1314.99(1071.96,1572.66) |  | 18122.56(14422.94,22691.73) | 868.14(690.91,1087.02) |  | -40.30(-53.20,-20.87) | -33.98(-48.25,-12.50) | -0.88(-1.15,-0.61) |
| Lithuania | 24389.06(22883.07,25968.72) | 2201.90(2065.94,2344.52) |  | 2862.72(2480.53,3318.21) | 534.30(462.96,619.31) |  | -88.26(-89.61,-86.92) | -75.73(-78.52,-72.96) | -4.55(-4.87,-4.23) |
| Luxembourg | 725.02(645.57,826.99) | 821.71(731.67,937.29) |  | 507.26(401.64,657.39) | 375.97(297.69,487.25) |  | -30.04(-40.07,-19.89) | -54.25(-60.81,-47.61) | -2.33(-2.70,-1.97) |
| Madagascar | 208052.77(165872.45,251658.18) | 3093.27(2466.14,3741.58) |  | 191848.57(145460.01,246021.24) | 1283.80(973.38,1646.31) |  | -7.79(-32.06,25.65) | -58.50(-69.42,-43.45) | -2.40(-2.58,-2.21) |
| Malawi | 288901.38(229466.25,350693.00) | 5183.68(4117.25,6292.39) |  | 139527.99(91729.88,202082.08) | 1317.44(866.12,1908.08) |  | -51.70(-65.27,-30.69) | -74.58(-81.72,-63.53) | -4.33(-4.51,-4.14) |
| Malaysia | 71729.57(60354.12,82488.38) | 859.76(723.41,988.72) |  | 42353.39(37268.80,47934.48) | 412.99(363.41,467.42) |  | -40.95(-49.59,-27.53) | -51.96(-58.99,-41.04) | -2.37(-2.69,-2.05) |
| Maldives | 4078.01(3333.07,4999.15) | 3177.42(2596.99,3895.13) |  | 850.02(712.55,1030.27) | 652.33(546.83,790.65) |  | -79.16(-83.63,-72.50) | -79.47(-83.87,-72.91) | -4.54(-5.40,-3.67) |
| Mali | 269085.61(190716.14,354395.72) | 5440.62(3856.07,7165.50) |  | 345558.63(227595.27,453038.85) | 2427.86(1599.06,3183.00) |  | 28.42(-12.93,87.80) | -55.38(-69.75,-34.74) | -2.32(-2.56,-2.08) |
| Malta | 705.90(584.85,856.36) | 612.49(507.46,743.05) |  | 398.90(320.72,497.91) | 475.13(382.01,593.06) |  | -43.49(-49.21,-37.40) | -22.43(-30.28,-14.06) | -0.94(-1.18,-0.69) |
| Marshall Islands | 236.27(189.90,285.89) | 882.18(709.05,1067.46) |  | 219.61(165.23,283.06) | 951.83(716.15,1226.87) |  | -7.05(-32.20,27.84) | 7.90(-21.30,48.40) | 0.04(-0.46,0.54) |
| Mauritania | 23092.17(17879.12,28171.30) | 2036.49(1576.75,2484.42) |  | 22010.86(16039.25,28819.81) | 943.79(687.74,1235.75) |  | -4.68(-33.27,33.94) | -53.66(-67.56,-34.88) | -2.44(-2.86,-2.03) |
| Mauritius | 3973.64(3739.63,4250.94) | 922.36(868.04,986.72) |  | 1348.40(1205.46,1492.23) | 454.15(406.01,502.60) |  | -66.07(-69.46,-62.37) | -50.76(-55.69,-45.40) | -2.41(-2.74,-2.07) |
| Mexico | 1008115.93(920832.40,1113016.70) | 2324.61(2123.34,2566.50) |  | 307080.37(259072.77,366653.00) | 711.64(600.39,849.70) |  | -69.54(-73.65,-64.39) | -69.39(-73.52,-64.21) | -3.21(-3.60,-2.83) |
| Micronesia (Federated States of) | 766.81(602.16,948.62) | 1333.64(1047.28,1649.84) |  | 334.50(267.00,410.94) | 807.10(644.23,991.54) |  | -56.38(-66.19,-41.75) | -39.48(-53.09,-19.19) | -1.59(-2.68,-0.50) |
| Monaco | 37.15(30.04,45.77) | 766.88(620.13,944.82) |  | 36.26(30.20,43.41) | 535.63(446.14,641.27) |  | -2.40(-19.49,16.33) | -30.15(-42.39,-16.75) | -1.62(-1.86,-1.38) |
| Mongolia | 55074.83(45709.33,64448.75) | 4851.88(4026.82,5677.69) |  | 17909.42(14979.16,21171.46) | 1364.32(1141.10,1612.82) |  | -67.48(-74.30,-58.09) | -71.88(-77.78,-63.76) | -3.46(-3.86,-3.06) |
| Montenegro | 1871.35(1535.93,2270.43) | 872.35(715.99,1058.38) |  | 707.91(561.97,902.37) | 470.72(373.67,600.02) |  | -62.17(-66.75,-57.32) | -46.04(-52.57,-39.12) | -2.07(-2.39,-1.74) |
| Morocco | 328594.44(251504.30,415182.82) | 2633.00(2015.28,3326.83) |  | 95823.69(76170.32,121099.40) | 741.92(589.75,937.62) |  | -70.84(-78.98,-60.46) | -71.82(-79.69,-61.80) | -3.78(-4.01,-3.56) |
| Mozambique | 360003.49(268021.24,446782.14) | 4755.22(3540.24,5901.46) |  | 265967.47(170732.56,414435.98) | 1498.06(961.65,2334.31) |  | -26.12(-50.11,15.05) | -68.50(-78.73,-50.94) | -3.30(-3.52,-3.08) |
| Myanmar | 1124243.88(813124.11,1457331.36) | 5882.63(4254.69,7625.51) |  | 404636.06(326394.47,507293.52) | 1948.35(1571.61,2442.65) |  | -64.01(-73.17,-49.00) | -66.88(-75.31,-53.07) | -3.40(-4.91,-1.86) |
| Namibia | 11724.29(9487.92,14437.03) | 1539.27(1245.66,1895.42) |  | 12007.26(9421.62,16082.36) | 1120.72(879.39,1501.08) |  | 2.41(-23.34,39.42) | -27.19(-45.50,-0.88) | -0.38(-0.84,0.09) |
| Nauru | 76.93(61.73,94.85) | 1476.56(1184.76,1820.49) |  | 68.90(52.53,91.52) | 1343.31(1024.08,1784.34) |  | -10.44(-33.89,22.68) | -9.02(-32.84,24.62) | -0.36(-1.08,0.36) |
| Nepal | 407405.56(306347.81,521280.47) | 3936.74(2960.22,5037.10) |  | 136655.11(114377.09,165344.39) | 1097.23(918.36,1327.59) |  | -66.46(-74.74,-53.82) | -72.13(-79.01,-61.63) | -3.71(-4.50,-2.91) |
| Netherlands | 17909.94(15505.90,21162.58) | 467.25(404.53,552.11) |  | 10290.24(8185.75,13350.67) | 278.79(221.77,361.70) |  | -42.54(-48.70,-36.88) | -40.33(-46.72,-34.45) | -1.55(-1.85,-1.24) |
| New Zealand | 11595.64(9914.58,13729.99) | 1053.11(900.43,1246.95) |  | 9997.92(8118.06,12629.79) | 767.02(622.80,968.93) |  | -13.78(-21.24,-6.77) | -27.17(-33.47,-21.24) | -0.58(-0.94,-0.22) |
| Nicaragua | 55790.01(47324.37,63565.66) | 2479.26(2103.06,2824.81) |  | 16667.64(13750.82,20112.64) | 641.25(529.03,773.78) |  | -70.12(-75.89,-63.72) | -74.14(-79.12,-68.59) | -4.53(-5.66,-3.38) |
| Niger | 349039.06(264995.53,448667.45) | 7194.32(5462.03,9247.83) |  | 403871.78(244506.88,595671.09) | 2591.12(1568.68,3821.65) |  | 15.71(-26.57,76.94) | -63.98(-77.14,-44.93) | -3.42(-3.71,-3.13) |
| Nigeria | 1740479.47(1442047.86,2026349.54) | 3587.21(2972.13,4176.41) |  | 2573775.64(1444453.97,3357366.92) | 2014.35(1130.49,2627.62) |  | 47.88(-15.41,102.17) | -43.85(-67.88,-23.23) | -1.59(-1.84,-1.33) |
| Niue | 10.52(8.44,12.91) | 1028.95(825.48,1262.47) |  | 13.98(12.70,15.50) | 2648.38(2405.34,2934.91) |  | 32.95(5.99,68.65) | 157.39(105.19,226.51) | 0.76(-0.58,2.13) |
| North Macedonia | 10775.04(8821.07,12308.17) | 1546.06(1265.69,1766.04) |  | 2307.45(1850.34,2878.22) | 516.72(414.36,644.53) |  | -78.59(-82.10,-73.80) | -66.58(-72.07,-59.11) | -3.66(-3.93,-3.40) |
| Northern Mariana Islands | 102.20(83.14,125.04) | 630.23(512.68,771.08) |  | 86.84(74.77,99.62) | 579.04(498.57,664.25) |  | -15.03(-30.81,5.72) | -8.12(-25.19,14.32) | 0.33(-0.43,1.10) |
| Norway | 6895.88(5961.85,8125.28) | 619.83(535.88,730.33) |  | 4163.07(3274.26,5400.68) | 335.34(263.74,435.03) |  | -39.63(-46.22,-33.23) | -45.90(-51.81,-40.17) | -1.63(-1.83,-1.43) |
| Oman | 12549.92(9963.85,15892.08) | 1252.12(994.10,1585.57) |  | 6498.11(5780.82,7245.00) | 435.58(387.50,485.64) |  | -48.22(-60.05,-33.13) | -65.21(-73.16,-55.07) | -2.50(-2.95,-2.04) |
| Pakistan | 1465024.60(1150667.94,1809086.31) | 2403.12(1887.48,2967.50) |  | 1646024.12(1325803.09,1990676.33) | 1507.02(1213.84,1822.57) |  | 12.35(-10.85,46.78) | -37.29(-50.24,-18.08) | -0.95(-1.79,-0.11) |
| Palau | 128.06(100.01,157.25) | 2077.08(1622.22,2550.66) |  | 63.18(53.40,75.34) | 1426.74(1205.82,1701.32) |  | -50.66(-60.46,-37.45) | -31.31(-44.94,-12.92) | -0.90(-1.05,-0.74) |
| Palestine | 14315.12(11547.80,17067.22) | 1199.35(967.50,1429.92) |  | 10860.01(9181.31,12754.55) | 451.03(381.31,529.71) |  | -24.14(-39.29,-3.51) | -62.39(-69.90,-52.17) | -2.66(-2.90,-2.41) |
| Panama | 20198.37(17898.19,22876.84) | 1858.12(1646.52,2104.52) |  | 11913.64(9985.66,14254.85) | 790.43(662.52,945.76) |  | -41.02(-50.10,-30.10) | -57.46(-64.01,-49.59) | -2.66(-2.86,-2.47) |
| Papua New Guinea | 49383.43(36488.05,63776.70) | 2319.95(1714.15,2996.12) |  | 95818.09(73881.22,125431.94) | 1955.85(1508.07,2560.33) |  | 94.03(51.14,153.52) | -15.69(-34.33,10.16) | -0.81(-1.57,-0.04) |
| Paraguay | 30911.91(26118.76,36131.32) | 1493.98(1262.33,1746.23) |  | 21610.20(17493.95,26993.41) | 809.17(655.04,1010.74) |  | -30.09(-45.55,-12.65) | -45.84(-57.81,-32.33) | -1.85(-2.07,-1.64) |
| Peru | 564117.63(431259.16,643734.38) | 5297.02(4049.49,6044.62) |  | 140051.34(106918.81,180122.04) | 1119.47(854.63,1439.77) |  | -75.17(-81.13,-66.56) | -78.87(-83.94,-71.54) | -5.08(-5.46,-4.69) |
| Philippines | 723762.20(649701.77,802336.89) | 2258.04(2026.99,2503.19) |  | 377432.14(330485.06,430527.24) | 842.90(738.06,961.48) |  | -47.85(-55.31,-38.67) | -62.67(-68.01,-56.10) | -1.93(-2.75,-1.11) |
| Poland | 147840.98(133647.29,165314.81) | 1187.53(1073.52,1327.88) |  | 29655.55(24075.37,37477.76) | 385.21(312.72,486.81) |  | -79.94(-82.21,-77.23) | -67.56(-71.22,-63.19) | -3.52(-3.72,-3.33) |
| Portugal | 30967.28(28060.84,34570.45) | 1038.84(941.34,1159.71) |  | 6010.20(4956.73,7402.88) | 313.56(258.60,386.22) |  | -80.59(-82.86,-78.22) | -69.82(-73.34,-66.12) | -4.01(-4.38,-3.65) |
| Puerto Rico | 9921.41(9010.37,11035.31) | 744.80(676.41,828.42) |  | 2140.87(1712.84,2739.07) | 327.61(262.11,419.15) |  | -78.42(-81.40,-74.43) | -56.01(-62.09,-47.88) | -1.93(-3.54,-0.29) |
| Qatar | 1755.14(1470.59,2095.33) | 1146.56(960.67,1368.78) |  | 2255.53(1882.55,2700.06) | 377.75(315.29,452.20) |  | 28.51(2.04,56.39) | -67.05(-73.84,-59.91) | -2.94(-3.33,-2.55) |
| Republic of Korea | 255022.17(225184.03,284360.72) | 1601.18(1413.84,1785.39) |  | 27013.13(21717.34,34269.19) | 322.23(259.06,408.78) |  | -89.41(-91.45,-86.80) | -79.88(-83.76,-74.92) | -5.27(-5.60,-4.94) |
| Republic of Moldova | 48652.64(44058.48,53985.76) | 3076.79(2786.26,3414.06) |  | 6248.19(5230.93,7468.40) | 897.42(751.31,1072.68) |  | -87.16(-89.29,-84.74) | -70.83(-75.67,-65.35) | -3.97(-4.10,-3.83) |
| Romania | 192848.07(177828.90,210057.40) | 2570.33(2370.15,2799.70) |  | 27446.62(23092.93,32642.36) | 679.23(571.48,807.80) |  | -85.77(-87.45,-84.12) | -73.57(-76.69,-70.52) | -4.41(-4.58,-4.24) |
| Russian Federation | 994752.76(946485.25,1057569.18) | 2201.51(2094.69,2340.53) |  | 234801.61(215954.63,264481.86) | 694.89(639.11,782.73) |  | -76.40(-77.58,-74.99) | -68.44(-70.02,-66.56) | -4.14(-4.66,-3.62) |
| Rwanda | 173766.17(137213.17,209715.34) | 4198.59(3315.39,5067.21) |  | 77402.41(55902.53,102730.79) | 1202.26(868.31,1595.67) |  | -55.46(-66.76,-36.43) | -71.37(-78.63,-59.14) | -4.57(-4.96,-4.18) |
| Saint Kitts and Nevis | 295.10(265.89,323.86) | 1606.31(1447.35,1762.87) |  | 122.82(105.25,142.40) | 873.39(748.45,1012.68) |  | -58.38(-64.39,-51.99) | -45.63(-53.48,-37.28) | -1.88(-2.27,-1.49) |
| Saint Lucia | 1012.45(878.09,1141.09) | 1516.18(1314.96,1708.82) |  | 347.54(293.77,412.72) | 828.31(700.15,983.64) |  | -65.67(-71.71,-58.23) | -45.37(-54.97,-33.53) | -1.94(-2.41,-1.47) |
| Saint Vincent and the Grenadines | 983.45(855.91,1121.26) | 1829.58(1592.31,2085.96) |  | 326.65(281.80,382.22) | 969.18(836.10,1134.05) |  | -66.78(-72.55,-59.77) | -47.03(-56.22,-35.84) | -1.66(-2.15,-1.16) |
| Samoa | 1349.59(1126.82,1577.71) | 1473.44(1230.23,1722.50) |  | 687.24(541.62,852.66) | 680.83(536.56,844.70) |  | -49.08(-59.15,-37.35) | -53.79(-62.93,-43.15) | -1.10(-2.82,0.64) |
| San Marino | 30.55(25.02,38.04) | 510.67(418.30,635.87) |  | 20.00(14.57,27.27) | 320.62(233.54,437.04) |  | -34.52(-44.90,-25.31) | -37.22(-47.16,-28.38) | -1.48(-1.54,-1.43) |
| Sao Tome and Principe | 1622.87(1289.04,2046.13) | 2322.23(1844.55,2927.89) |  | 921.54(753.99,1164.62) | 905.22(740.63,1144.00) |  | -43.22(-56.12,-26.45) | -61.02(-69.88,-49.51) | -4.22(-4.70,-3.73) |
| Saudi Arabia | 196663.13(161197.11,244867.67) | 2395.00(1963.09,2982.04) |  | 48172.72(40164.68,57728.76) | 475.39(396.36,569.69) |  | -75.50(-81.49,-68.46) | -80.15(-85.00,-74.44) | -4.75(-4.96,-4.54) |
| Senegal | 167765.16(135386.89,207745.20) | 3772.60(3044.49,4671.64) |  | 108690.48(78578.75,145963.53) | 1339.33(968.28,1798.62) |  | -35.21(-51.26,-11.89) | -64.50(-73.29,-51.72) | -3.04(-3.39,-2.69) |
| Serbia | 32492.00(28258.98,37603.06) | 1119.99(974.08,1296.17) |  | 7884.15(5953.56,10329.23) | 420.74(317.71,551.22) |  | -75.74(-79.91,-71.57) | -62.43(-68.89,-55.99) | -3.33(-3.53,-3.13) |
| Seychelles | 322.67(282.69,363.77) | 1032.81(904.86,1164.37) |  | 92.98(77.95,111.97) | 304.57(255.33,366.78) |  | -71.18(-76.39,-64.47) | -70.51(-75.84,-63.64) | -1.43(-2.25,-0.61) |
| Sierra Leone | 123543.80(94707.67,152994.55) | 5601.10(4293.76,6936.31) |  | 111389.57(71259.75,151172.44) | 2447.77(1565.92,3321.99) |  | -9.84(-40.62,35.92) | -56.30(-71.22,-34.12) | -2.51(-2.89,-2.12) |
| Singapore | 4433.87(3811.06,5271.95) | 481.43(413.81,572.43) |  | 2566.18(2010.39,3363.31) | 245.94(192.68,322.34) |  | -42.12(-48.75,-35.64) | -48.91(-54.76,-43.19) | -1.95(-2.10,-1.80) |
| Slovakia | 18771.18(16633.43,21708.71) | 1069.45(947.66,1236.82) |  | 6691.23(5572.30,8163.27) | 596.84(497.04,728.15) |  | -64.35(-68.22,-60.65) | -44.19(-50.24,-38.39) | -1.79(-1.92,-1.66) |
| Slovenia | 5262.93(4467.33,6328.52) | 945.26(802.36,1136.64) |  | 1887.37(1440.38,2533.66) | 466.22(355.80,625.87) |  | -64.14(-68.78,-59.95) | -50.68(-57.06,-44.91) | -2.19(-2.50,-1.87) |
| Solomon Islands | 2770.69(2038.77,3528.61) | 1423.66(1047.58,1813.10) |  | 3516.42(2778.12,4293.40) | 1063.19(839.96,1298.11) |  | 26.91(1.80,63.02) | -25.32(-40.10,-4.07) | -0.30(-0.93,0.34) |
| Somalia | 196948.69(129341.73,272788.75) | 4161.76(2733.15,5764.35) |  | 288172.21(175715.20,460197.53) | 2254.09(1374.45,3599.68) |  | 46.32(-0.41,116.67) | -45.84(-63.14,-19.79) | -1.67(-2.16,-1.17) |
| South Africa | 362418.48(311591.14,412881.79) | 2056.66(1768.22,2343.03) |  | 223787.12(188330.23,262674.68) | 1123.54(945.53,1318.78) |  | -38.25(-48.34,-26.25) | -45.37(-54.29,-34.75) | -1.64(-1.88,-1.39) |
| South Sudan | 136668.87(102917.21,173598.75) | 4159.04(3131.93,5282.87) |  | 174716.14(127848.29,227433.37) | 3183.98(2329.88,4144.69) |  | 27.84(-5.13,79.66) | -23.44(-43.19,7.59) | -0.49(-1.03,0.05) |
| Spain | 75143.53(66680.09,85988.85) | 674.11(598.19,771.41) |  | 31165.30(24648.33,40474.69) | 353.85(279.86,459.56) |  | -58.53(-63.72,-52.90) | -47.51(-54.08,-40.39) | -1.95(-2.11,-1.79) |
| Sri Lanka | 112015.26(98133.38,124920.31) | 1547.15(1355.41,1725.39) |  | 37805.89(30669.77,46157.73) | 547.92(444.49,668.96) |  | -66.25(-72.51,-57.91) | -64.59(-71.15,-55.84) | -3.43(-5.41,-1.42) |
| Sudan | 724596.24(513839.62,961773.27) | 6559.73(4651.76,8706.88) |  | 313673.01(238389.47,409080.68) | 1467.47(1115.27,1913.82) |  | -56.71(-68.16,-35.97) | -77.63(-83.55,-66.91) | -4.55(-4.75,-4.36) |
| Suriname | 3960.65(2702.92,4651.28) | 2316.29(1580.74,2720.19) |  | 2115.92(1743.57,2568.47) | 1114.50(918.38,1352.87) |  | -46.58(-59.22,-21.45) | -51.88(-63.27,-29.25) | -2.10(-2.42,-1.78) |
| Sweden | 10870.84(8943.52,13381.05) | 515.93(424.46,635.07) |  | 7372.48(5603.47,9924.20) | 305.24(232.00,410.89) |  | -32.18(-38.43,-25.85) | -40.84(-46.29,-35.31) | -1.53(-1.78,-1.28) |
| Switzerland | 11710.08(10073.45,13961.35) | 742.47(638.70,885.21) |  | 6806.06(5278.26,8954.41) | 386.14(299.46,508.03) |  | -41.88(-47.89,-35.53) | -47.99(-53.37,-42.31) | -2.10(-2.27,-1.93) |
| Syrian Arab Republic | 108307.26(87261.89,130728.62) | 1473.18(1186.93,1778.15) |  | 30301.06(24940.76,36634.68) | 556.99(458.46,673.42) |  | -72.02(-78.30,-63.46) | -62.19(-70.67,-50.62) | -3.02(-3.42,-2.62) |
| Taiwan (Province of China) | 111541.55(107668.85,115941.68) | 1526.10(1473.11,1586.30) |  | 14660.19(13240.66,16122.92) | 362.39(327.30,398.54) |  | -86.86(-88.14,-85.60) | -76.25(-78.57,-73.99) | -4.66(-5.32,-4.01) |
| Tajikistan | 102617.56(87408.87,118013.38) | 3569.95(3040.86,4105.56) |  | 72556.53(57797.69,91638.56) | 1628.60(1297.33,2056.92) |  | -29.29(-44.69,-5.91) | -54.38(-64.31,-39.29) | -3.04(-3.48,-2.60) |
| Thailand | 482166.20(405333.41,555654.37) | 2119.71(1781.93,2442.78) |  | 107495.07(94098.43,122326.19) | 787.61(689.46,896.28) |  | -77.71(-81.23,-73.49) | -62.84(-68.72,-55.82) | -3.34(-3.86,-2.82) |
| Timor-Leste | 16488.34(12429.74,21579.62) | 4054.54(3056.51,5306.50) |  | 10303.80(8535.33,12455.01) | 1503.26(1245.25,1817.11) |  | -37.51(-52.16,-18.21) | -62.92(-71.62,-51.47) | -3.96(-4.47,-3.45) |
| Togo | 61378.92(48216.48,75053.87) | 2851.22(2239.79,3486.46) |  | 59308.83(35875.69,82052.18) | 1420.43(859.21,1965.13) |  | -3.37(-33.63,37.13) | -50.18(-65.78,-29.30) | -1.97(-2.21,-1.72) |
| Tokelau | 6.36(4.89,8.16) | 838.74(644.77,1075.96) |  | 10.61(8.54,12.73) | 2074.19(1669.99,2489.38) |  | 66.81(33.69,117.54) | 147.30(98.20,222.50) | -0.45(-1.80,0.91) |
| Tonga | 601.34(487.60,736.24) | 1126.90(913.75,1379.71) |  | 376.09(300.42,476.77) | 758.52(605.90,961.56) |  | -37.46(-52.30,-15.86) | -32.69(-48.66,-9.45) | -0.79(-1.32,-0.27) |
| Trinidad and Tobago | 7467.73(6733.83,8258.97) | 1437.56(1296.28,1589.87) |  | 2369.41(1983.59,2837.94) | 650.97(544.97,779.69) |  | -68.27(-73.86,-61.39) | -54.72(-62.70,-44.89) | -2.32(-2.57,-2.07) |
| Tunisia | 81978.09(67220.46,98751.14) | 2054.56(1684.70,2474.93) |  | 19018.77(15149.54,23743.47) | 530.43(422.52,662.20) |  | -76.80(-82.34,-70.14) | -74.18(-80.35,-66.77) | -4.00(-4.12,-3.88) |
| Turkey | 468799.54(385807.41,550338.88) | 1757.01(1445.97,2062.61) |  | 105572.04(92516.76,122066.31) | 427.21(374.38,493.95) |  | -77.48(-81.89,-72.23) | -75.69(-80.45,-70.02) | -4.75(-5.51,-3.99) |
| Turkmenistan | 74749.32(65862.05,83556.52) | 3964.99(3493.58,4432.16) |  | 19765.93(17083.93,22993.02) | 1008.18(871.38,1172.78) |  | -73.56(-77.86,-68.09) | -74.57(-78.71,-69.32) | -4.95(-5.38,-4.51) |
| Tuvalu | 91.21(68.89,115.35) | 2134.40(1612.19,2699.42) |  | 38.35(30.62,48.19) | 780.53(623.18,980.90) |  | -57.96(-68.59,-40.72) | -63.43(-72.68,-48.43) | -3.55(-4.98,-2.10) |
| Uganda | 288416.91(220365.03,360727.40) | 2800.77(2139.93,3502.96) |  | 321761.44(213089.37,450839.69) | 1289.96(854.29,1807.44) |  | 11.56(-19.78,61.38) | -53.94(-66.88,-33.37) | -2.28(-2.46,-2.09) |
| Ukraine | 307160.76(276921.51,341620.13) | 2040.71(1839.80,2269.65) |  | 58523.99(50309.77,67405.70) | 692.98(595.72,798.15) |  | -80.95(-83.50,-78.07) | -66.04(-70.60,-60.92) | -4.03(-4.35,-3.72) |
| United Arab Emirates | 9607.61(7920.04,11560.56) | 1358.89(1120.20,1635.11) |  | 5874.00(4976.20,6907.68) | 346.53(293.57,407.51) |  | -38.86(-49.45,-26.55) | -74.50(-78.92,-69.36) | -3.85(-4.45,-3.25) |
| United Kingdom | 83743.18(72342.37,99545.26) | 564.54(487.68,671.06) |  | 45313.86(35856.04,59183.03) | 289.46(229.04,378.05) |  | -45.89(-51.00,-40.72) | -48.73(-53.57,-43.83) | -1.82(-1.95,-1.70) |
| United Republic of Tanzania | 522328.31(421912.77,617065.19) | 3500.10(2827.22,4134.93) |  | 422402.91(296444.22,613851.25) | 1368.85(960.66,1989.26) |  | -19.13(-40.52,13.88) | -60.89(-71.24,-44.93) | -2.52(-2.70,-2.34) |
| United States of America | 589184.33(551761.91,642064.82) | 795.71(745.17,867.12) |  | 353509.01(322443.71,393007.63) | 434.75(396.55,483.33) |  | -40.00(-44.37,-35.55) | -45.36(-49.35,-41.31) | -1.71(-1.99,-1.42) |
| United States Virgin Islands | 529.46(455.21,612.12) | 1269.23(1091.25,1467.38) |  | 103.46(82.61,129.39) | 567.86(453.44,710.15) |  | -80.46(-84.61,-74.97) | -55.26(-64.76,-42.68) | -2.23(-2.79,-1.67) |
| Uruguay | 27309.48(25593.81,29163.03) | 2535.64(2376.34,2707.74) |  | 6510.92(5618.56,7650.64) | 720.77(621.98,846.94) |  | -76.16(-78.89,-72.97) | -71.57(-74.83,-67.77) | -4.33(-4.59,-4.07) |
| Uzbekistan | 352243.98(315118.56,394966.49) | 3299.46(2951.71,3699.64) |  | 167807.88(142241.80,198586.64) | 1330.74(1128.00,1574.82) |  | -52.36(-60.56,-42.96) | -59.67(-66.61,-51.71) | -3.10(-3.35,-2.85) |
| Vanuatu | 887.34(678.85,1105.80) | 1063.34(813.50,1325.13) |  | 1253.52(993.43,1530.84) | 850.20(673.79,1038.29) |  | 41.27(13.56,79.28) | -20.04(-35.73,1.47) | -0.78(-1.69,0.14) |
| Venezuela (Bolivarian Republic of) | 193229.34(178797.16,209267.37) | 2129.00(1969.98,2305.70) |  | 124440.17(101111.28,154093.97) | 1421.88(1155.32,1760.71) |  | -35.60(-47.18,-21.27) | -33.21(-45.22,-18.36) | -1.44(-2.93,0.08) |
| Viet Nam | 1191985.37(972699.71,1414645.51) | 3540.53(2889.19,4201.89) |  | 427752.60(342258.34,528289.19) | 1352.84(1082.45,1670.80) |  | -64.11(-71.84,-54.31) | -61.79(-70.01,-51.35) | -2.76(-2.99,-2.54) |
| Yemen | 326852.36(235184.24,434151.07) | 3887.36(2797.12,5163.49) |  | 213215.30(158352.04,271577.65) | 1220.32(906.31,1554.35) |  | -34.77(-52.64,-5.36) | -68.61(-77.21,-54.46) | -3.69(-3.83,-3.55) |
| Zambia | 183314.99(148679.34,220935.55) | 3899.91(3163.06,4700.26) |  | 125219.62(87917.95,173827.52) | 1197.79(840.98,1662.75) |  | -31.69(-52.37,-3.65) | -69.29(-78.58,-56.68) | -3.62(-3.90,-3.34) |
| Zimbabwe | 75143.74(56095.07,93692.92) | 1247.74(931.45,1555.75) |  | 129570.87(103194.43,160784.71) | 1622.83(1292.48,2013.78) |  | 72.43(33.33,125.06) | 30.06(0.57,69.76) | 2.19(1.61,2.77) |
